# Taxonomic synopsis of the subtribe Physoderina (Coleoptera, Carabidae, Lebiini), with species revisions of eight genera

**DOI:** 10.3897/zookeys.284.3983

**Published:** 2013-04-04

**Authors:** Hongliang Shi, Hongzhang Zhou, Hongbin Liang

**Affiliations:** 1 Key Laboratory of Zoological Systematics and Evolution, Institute of Zoology, Chinese Academy of Sciences, Beijing 100101, China; 2 Graduate School of Chinese Academy of Sciences, Beijing 100039, China; 3 Southwest Forestry University, Kunming 650224, Yunnan, China

**Keywords:** Oriental Region, Physoderina, key, new species, new combination, new synonym

## Abstract

Ten genera of Physoderina from the Oriental Region are diagnosed and described, and twenty six species representing eight genera (*Paraphaea* Bates, *Anchista* Nietner, *Metallanchista* **gen. n.**, *Diamella* **nom. n.**, *Allocota* Motschulsky, *Orionella* Jedlička, *Endynomena* Chaudoir and *Dasiosoma* Britton (Oriental species only)) are revised. Keys to genera and species are provided, along with distribution maps, habitus images, photographs of the name-bearing types, and illustrations of male and female genitalia of available species. The female internal reproductive system is illustrated for fourteen species. Two genera, *Anchista* and *Taicona*, previously placed in Calleidina, are moved into Physoderina. One new genus is described: *Metallanchista*, **gen. n.** (type species *Metallanchista laticollis*, sp. n.). Two new generic synonyms are proposed: *Taicona* Bates, 1873, **junior synonym** of *Allocota* Motschulsky, 1859; *Teradaia* Habu, 1979a, **junior synonym** of *Dasiosoma* Britton, 1937. A new generic replacement name is proposed: *Diamella*, **nom. n.** for *Diamella* Jedlička, 1952 (junior homonym of *Diamella* Gude, 1913). The status of *Paraphaea* Bates, 1873 is **resurrected** from synonym of *Anchista* Nietner, 1856. Five new species are described: *Paraphaea minor* Shi & Liang, **sp. n.** (Hoa-Binh, Tonkin, Vietnam), *Anchista pilosa* Shi & Liang, **sp. n.** (Chikkangalur, Bangalore, India), *Metallanchista laticollis* Shi & Liang, **sp. n.** (PhaTo env., Chumphon prov., Thailand), *Allocota* *bicolor* Shi & Liang, **sp. n.** (Dengga to Mafengshan, Ruili, Yunnan, China), *Dasiosoma quadraticolle* Shi & Liang, **sp. n.** (Menglun Botanical Garden, Yunnan, China). Fourteen new combinations are proposed: *Paraphaea binotata* (Dejean, 1825), **comb. n.** from *Anchista*; *Paraphaea formosana* (Jedlička, 1946), **comb. n.** from *Anchista*; *Paraphaea philippinensis* (Jedlička, 1935b), **comb. n.** from *Allocota*; *Metallanchista perlaeta* (Kirschenhofer, 1994), **comb. n.** from *Allocota*; *Physodera andrewesi* (Jedlička, 1934), **comb. n.** from *Allocota*; *Diamella cupreomicans* (Oberthür, 1883), **comb. n.** from *Physodera*; *Diamella arrowi* (Jedlička, 1935a), **comb. n.** from *Allocota*; *Allocota aurata* (Bates, 1873), **comb. n.** from *Taicona*; *Dasiosoma bellum* (Habu, 1979a), **comb. n.** from *Teradaia*; *Dasiosoma indicum* (Kirschenhofer, 2011), **comb. n.** from *Diamella*; *Dasiosoma maindroni* (Tian & Deuve, 2001), **comb. n.** from *Lachnoderma*; *Dasiosoma hirsutum* (Bates, 1873), **comb. n.** from *Lachnoderma*; *Orionella discoidalis* (Bates, 1892), **comb. n.** from *Anchista*; *Orionella kathmanduensis* (Kirschenhofer, 1994), **comb. n.** from *Lachnoderma*. Five names are newly placed as junior synonyms: *Paraphaea eurydera* (Chaudoir, 1877), **junior synonym** of *Paraphaea binotata* (Dejean, 1825); *Anchista glabra* Chaudoir, 1877, and *Anchista nepalensis* Kirschenhofer, 1994, **junior synonyms** of *Anchista fenestrata* (Schmidt-Göbel, 1846); *Allocota caerulea* Andrewes, 1933, **junior synonym** of *Allocota viridipennis* Motschulsky, 1859; *Allocota perroti* (Jedlička, 1963), **junior synonym** of *Allocota aurata* (Bates, 1873). One new replacement name is proposed: *Dasiosoma basilewskyi*, **nom. n.** for *Dasiosoma hirsutum* Basilewsky, 1949 (secondary junior homonym of *Dasiosoma hirsutum* (Bates, 1892)). One species is downgraded to subspecies rank: *Anchista fenestrata subpubescens* Chaudoir, 1877, **new rank**.

## Introduction

Physoderina is one of the subtribes of the tribe Lebiini, arboreal truncatipennes carabid beetles, distributed in the Oriental and Afrotropical Regions. The stem *Physoder-* was first used in a family-group named Physodérides by Chaudoir (1877: 213) when treating the genera *Physodera* Eschscholtz, 1829, *Allocota* Motschulsky, 1859, *Lachnoderma* W. J. Macleay, 1873, *Aspasiola* Chaudoir, 1877, and *Cryptobatis* Eschscholtz, 1829. Later, Bates (1892) included *Endynomena* Chaudoir, 1872, in the subfamily Physoderinae, and Britton (1937) erected a new genus *Dasiosoma*. Jedlička (1963) reviewed six genera of the subtribe under the group name Physoderi, including his two genera, *Diamella* Jedlička, 1952, and *Orionella* Jedlička, 1963, with the exclusion of New World genera (*Cryptobatis* group). Habu (1979b) clarified the definition of *Endynomena* and *Orionella*, and described a new genus *Teradaia* Habu (1979a). In addition to Jedlička’s monograph (1963) and Habu’s work (1979a, b), species-level revisions or keys for this subtribe were contributed by Heller (1923, *Physodera*), Basilewsky (1949, *Dasiosoma*), Kirschenhofer (1994, *Anchista*, *Lachnoderma*, *Allocota*; 1996, *Lachnoderma*, *Allocota*), Tian and Deuve (2001, *Lachnoderma*). Currently, seven genera are recognized within the subtribe: *Dasiosoma* Britton, 1937 (4 species) from Africa, and six Oriental genera (*Allocota* Motschulsky, 1859 (8 species), *Diamella* Jedlička, 1952 (1 species), *Endynomena* Chaudoir, 1872 (1 species), *Lachnoderma* W. J. Macleay, 1873 (17 species), *Orionella* Jedlička, 1963 (1 species), and *Physodera* Eschscholtz, 1829 (13 species)) (Löbl and Smetana 2003, Lorenz 2005).

We studied Lebiini type and non-type specimens from several collections. Morphological comparisons reveal that Jedlička (1963) didn’t properly define the genera in his group Physoderi, and several species were assigned to inappropriate genera. *Allocota* of Jedlička (1963) and later modified by Kirschenhofer (1994, 1996) contained species belonging to other genera. *Anchista* and *Taicona* were previously placed in Calleidina but are more closely related to genera within Physoderina. *Anchista* contained some distantly related species, and the genus status of *Paraphaea* Bates, 1873, has to be resurrected to accommodate some of these species. Several new species are described to accommodate specimens that differ markedly from any known species.

The main purposes of this paper are to: (1) redefine each genus of Physoderina and describe a new genus; (2) provide a key to genera; (3) appropriately arrange each species to genus; (4) describe new species and propose new synonyms; (5) provide keys to species of seven small genera (keys to the two large genera, *Physodera* and *Lachnoderma*, will be provided in future papers, and African species of genus *Dasiosoma* are also omitted from the present key).

## Materials and methods

### Materials

This work is based on the examination of 584 specimens, including 197 types. Many of the specimens in the course of this study were borrowed from or examined in the following collections:

CAS California Academy of Sciences, San Francisco, USA

CBW Collection of Wenxuan BI, Shanghai, China

CCA Collection of Achille Casale, Torino, Italy

CCCC Collection of Changchin CHEN, Tianjin, China

CDW Collection of David Wrase, Berlin, Germany

CMB Collection of Martin Baehr, München, Germany

CRS Collection of Riccardo Sciaky, Milano, Italy

HBUM Hebei University Museum, Baoding, China

IZAS Institute of Zoology, Chinese Academy of Science, Beijing, China

MNHN Muséum National d’Histoire Naturelle, Paris, France

MNHU Museum für Naturkunde der Humboldt-Universität zu Berlin, Berlin, Germany

MRAC Musée royal de l’Afrique centrale, Tervuren, Belgium

MSNG Museo Civico di Storia Naturale (Giacomo Doria), Genova, Italy

MTMB Magyar Természettudományi Múzeum, Budapest, Hungary

NHMB Naturhistorisches Museum, Basel, Switzerland

NHML The Natural History Museum, London, U. K.

NMPC Národní Muzeum Přírodovědecké Muzeum, Prague, Czech Republic

NNML Naturalis Nationaal Natuurhistorisch Museum, Leiden, Netherland

OMNH Osaka Museum Natural History, Osaka, Japan

SCAU South China Agricultural University, Guangzhou, China

SNSD Staatliches Museum für Tierkunde, Dresden, Germany

ZMUC Zoological Museum, University of Copenhagen, Copenhagen, Denmark

ZSM Zoologische Staatssammlungen, München, Germany

The following collections are cited in this paper, but we didn’t examine any specimens:

ANIC Australian National Insect Collection, CSIRO Division of Entomology, Canberra, Australia

BRIO Biosystematics Research Institute, Ottawa, Canada.

MAMU University of Sydney, Macleay Museum, Sydney, Australia

MZPW Museum and Institute of Zoology of the Polish Academy of Sciences, Warszawa, Poland

NHMW Naturhistorisches Museum, Wien, Austria

NIAES National Institute for Agro-Environmental Sciences, Japan.

ZMUM Moscow State University, Moscow, Russia

### Methods

This work was mainly based on the study of type material. If the type specimens were not available, a specimen from the type locality fitting the original description was used to represent the species. Two species were recognized as distinct based on differences in genitalia and external morphological characters in two populations, unless significant overlapping existed. When defining distinct genera, we emphasized differences in genitalia and secondary sexual characters (for example, setae number and emargination on terminal sternum, male adhesive hairs on tarsomeres, and shape of terminal labial palpomeres).

Body length was measured from apical margin of labrum to elytral apex; pronotum length (PL) was measured along its median line; pronotum width (PW) was the greatest width of pronotum; elytra length (EL) was measured from elytral base to apex; elytra width (EW) was the combined width of each elytron at its widest points. All measurements were made with the aid of an ocular micrometer in a Nikon SMZ-1500 or SMZ-1000 stereoscopic dissecting microscope.

Photographs of male and female genitalia were captured by a Nikon SMZ-1500 stereoscopic dissecting microscope fitted with a Canon 450D digital camera, or by a Nikon digital Sight DS-SM camera fitted to a Nikon SMZ-1500 stereoscopic dissecting microscope controlled by ACT-2U software. Photographs of habitus were captured by a Canon Macro 100 mm lens fitted with a Canon 450D digital camera or a Tamron SP 90 mm lens fitted with a Nikon D7000 digital camera. For each final image, several photographs were taken at different focal planes, combined with HELICON FOCUS software to get one synthesized photograph, and finally edited by Adobe PHOTOSHOP software. Distribution maps were created in Adobe PHOTOSHOP software based on examined materials and / or published records.

Male genitalia were dissected from the apex of the abdomen using forceps and put into 10% KOH solution at room temperature for 8–12 hours. The treated genitalia were transferred into glycerol for imaging and permanent storage. Female genitalia were prepared in a multi-step process: the apical one or two abdominal segments were dipped in 10% KOH solution at room temperature for 8–20 hours, then the genitalia were extracted from the abdominal segments and stained in Chlorozol Black E saturated solution in 70% ethanol for approximately ten seconds, and finally rinsed with 70% ethanol. The treated genitalia were kept in 70% ethanol for imaging, and then transferred into glycerol for permanent storage.

For each taxon, original and important taxonomic references are cited. Genus combination, information on name-bearing type, newly recorded localities, and other comments are listed in parentheses after each reference.

If syntypes were examined, a lectotype was assigned for the taxonomic purpose of fixing the species name to a single specimen and preventing further confusion. In this case, detailed information, including label data, body length, mounting method, and repository of the lectotype has been provided.

For type materials, specimens of very rare species, and important non-type specimens, full label data have been provided. Individual labels are separated by a semicolon and each line within one label is separated by a slash. All label text is cited in its original spelling, punctuation and language. Hand-written letters are cited in italic. Original italic or bold is ignored. If there is no special indication, it means these are white square labels with black writing, any other kind of label is indicated in square brackets. Red labels were added to the types of new species and lectotypes. For non-type material of common species, only the locality label is given in quotation marks.

We have provided detailed species descriptions, except for those with sufficient original descriptions or redescriptions. The male genitalia and female ovipositor have been described and illustrated for each species when available. Female internal reproductive systems have been studied for new species, representative species or species of unclear taxonomic status.

### Terminology

Most morphological terms in the present paper follow their general applications. When referring to the orientation of the median lobe of male genitalia, “left” or “right” was determined with the apex of the median lobe pointing posteriorly, and its base ventrally. A few more terms are introduced when describing internal sac structure of the aedeagus as follows: **Main flagellum** is the flagellum-like sclerite on the internal sac, nearly as long as the median lobe (Fig. 89). This is absent in *Anchista* Nietner and *Metallanchista* gen. n. (Figs 70–75). The base of the main flagellum, when expanded, forms a trumpet-like sclerite, and is called **trumpet-form expansion**. In some species, another fine flagellum-like sclerite is present near the apex of the median lobe; this is less than half the length of the main flagellum, and is called the **secondary flagellum**. **Apical bursa** is a small bursa-like area sometimes present on the apical orifice; it is finely scaled or sclerotized, and is visible outside the orifice (Fig. 89). In some species, the internal sac has an area distinctly spined or scaled which we call **spined area** or **scaled area**.

### Check list of Physoderina and Index

**Subtribe Physoderina Chaudoir**

**Genus *Paraphaea* Bates, 1873, status resurrected**

*Paraphaea binotata* (Dejean, 1825), comb. n.

*Calleida discophora* Chaudoir, 1852

*Paraphaea signifera* Bates, 1873

*Anchista eurydera* Chaudoir, 1877, syn. n.

*Paraphaea formosana* (Jedlička, 1946), comb. n.

*Paraphaea minor* Shi & Liang, sp. n.

*Paraphaea philippinensis* (Jedlička, 1935b), comb. n.

**Genus *Anchista* Nietner, 1856**

*Anchista brunnea* (Wiedemann, 1823)

*Anchista modesta* Nietner, 1856

*Anchista picea* Chaudoir, 1877

*Anchista fenestrata fenestrata* (Schmidt-Göbel, 1846)

*Anchista glabra* Chaudoir, 1877, syn. n.

*Anchista nepalensis* Kirschenhofer, 1994, syn. n.

*Anchista fenestrata subpubescens* Chaudoir, 1877, new rank

*Anchista nubila* Andrewes, 1931

*Anchista pilosa* Shi & Liang, sp. n.

**Genus *Metallanchista* Shi & Liang, gen. n.**

*Metallanchista laticollis* Shi & Liang, sp. n.

*Metallanchista perlaeta* (Kirschenhofer, 1994), comb. n.

**Genus *Physodera* Eschscholtz, 1829**

*Physodera amplicollis* van de Poll, 1889

*Physodera andrewesi* (Jedlička, 1934), comb. n.

*Physodera bacchusi* Darlington, 1971

*Physodera bifenestrata* Heller, 1923

*Physodera bousqueti* Mateu, 1990

*Physodera chalceres* Andrewes, 1930b

*Physodera cyanipennis* van de Poll, 1889

*Physodera dejeani* Eschscholtz, 1829

*Physodera diglena* Andrewes, 1930c

*Physodera eburata* Heller, 1923

*Physodera eschscholtzii eschscholtzii* Parry, 1849

*Physodera davidis* Fairmaire, 1887

*Physodera eschscholtzii sumatrensis* (Kirschenhofer, 1996)

*Physodera noctiluca* Mohnike, 1875

*Physodera parvicollis* van de Poll, 1889

**Genus *Diamella* Shi & Liang, nom. n.**

*Diamella* Jedlička, 1952 [homonym]

*Diamella kaszabi* (Jedlička, 1952)

*Diamella cupreomicans* (Oberthür, 1883), comb. n.

*Allocota aerata* Bates, 1892

*Diamella arrowi* (Jedlička, 1935a), comb. n.

**Genus *Allocota* Motschulsky, 1859**

*Taicona* Bates, 1873, syn. n.

*Allocota viridipennis* Motschulsky, 1859

*Allocota caerulea* Andrewes, 1933, syn. n.

*Allocota cyanipennis* Heller, 1923

*Allocota aurata* (Bates, 1873), comb. n.

*Taicona perroti* Jedlička, 1963, syn. n.

*Allocota bicolor* Shi & Liang, sp. n.

**Genus *Lachnoderma* W. J. Macleay, 1873**

*Lachnoderma asperum* Bates, 1883

*Lachnoderma biguttatum* Bates, 1892

*Lachnoderma rufithorax* Kirschenhofer, 1996

*Lachnoderma chebaling* Tian & Deuve, 2001

*Lachnoderma cheni* Tian & Deuve, 2001

*Lachnoderma cinctum* W. J. Macleay, 1873

*Lachnoderma confusum* Tian & Deuve, 2001

*Lachnoderma foveolatum* Sloane, 1915

*Lachnoderma metallicum* Tian & Deuve, 2001

*Lachnoderma nideki* Louwerens, 1952

*Lachnoderma philippinense* Jedlička, 1934

*Lachnoderma polybothris* Louwerens, 1967

*Lachnoderma tricolor* Andrewes, 1926

*Lachnoderma vietnamense* Kirschenhofer, 1996

*Lachnoderma yingdeicum* Tian & Deuve, 2001

**Genus *Dasiosoma* Britton, 1937**

*Teradaia* Habu, 1979a, syn. n.

*Dasiosoma testaceum* Britton, 1937

*Dasiosoma basilewskyi* Shi & Liang, nom. n.

*Dasiosoma hirsutum* Basilewsky, 1949 [secondary homonym]

*Dasiosoma sudanicum* Basilewsky, 1949

*Dasiosoma ivorense* Basilewsky, 1968

*Dasiosoma bellum* (Habu, 1979a), comb. n.

*Dasiosoma indicum* (Kirschenhofer, 2011), comb. n.

*Dasiosoma maindroni* (Tian & Deuve, 2001), comb. n.

*Dasiosoma hirsutum* (Bates, 1873), comb. n.

*Dasiosoma quadraticolle* Shi & Liang, sp. n.

**Genus *Orionella* Jedlička, 1963**

*Orionella lewisii* (Bates, 1873)

*Orionella obenbergeri* Jedlička, 1963

*Orionella discoidalis* (Bates, 1892), comb. n.

*Orionella kathmanduensis* (Kirschenhofer, 1994), comb. n.

**Genus *Endynomena* Chaudoir, 1872**

*Saronychium* Blackburn, 1877

*Endynomena pradieri* (Fairmaire, 1849a)

*Saronychium inconspicuum* Blackburn, 1877

*Endynomena huebneri* Fairmaire, 1878

*Thyreopterus paroecus* Csiki, 1915

#### Subtribe Physoderina

Chaudoir

Physodérides Chaudoir 1877: 213. Type genus: *Physodera* Eschscholtz, 1829.

##### Diagnosis.

Adults of this subtribe can be recognized by combination of the following characters: mandibles moderately to strongly widened; apex of ligula with four or more setae; palpifers without seta; mentum with tooth simple or bifid; front angles of pronotum with some setae longer or shorter; pronotal base usually more or less lobed; elytral dorsal setigerous pores, if distinct, at least with one present on base of 5th interval; elytral apex truncate, outer angles completely rounded, not angulate, sutural angles not projected; apex of 7th and 8th intervals more or less tumid; penultimate pore of elytral umbilical series not displaced laterally or medially; males with terminal sternum more or less emarginate; 4th tarsomere strongly bilobed; claws pectinate; median lobe of aedeagus usually with apical orifice opened apically, dorsal surface with some setae subapically, such setae generally fine but sometimes very strong, present around apical orifice; internal sac generally with a long flagellum-like sclerite; right paramere trifurcate, apex usually widened; apical segment of ovipositor without spine, apex with extension usually membranous; spermatheca inserted on bursa copulatrix or joining of common oviduct and bursa copulatrix.

Based on widened mandibles, bifid 4th tarsomere, pectinate claws, and female ovipositor characters, Calleidina may be more closely allied with Physoderina than any other subtribe of Lebiini from the Oriental Region. Members of Calleidina can be readily distinguished from those of Physoderina by the absence of setae on the pronotum front angles, except *Calleida sultana* Bates. But *Calleida sultana* has a totally different shape of the aedeagus and no setigerous pore on 5th interval.

##### Monophyly and relationships.

Reconstruction of phylogeny of Lebiini has been attempted by Ball et al. (1995) and Casale (1998), but their works did not focus on the systematic position of Physoderina. Their results support a close relationship between Physoderina and Metallicina + (Calleidina + Galerucidiina) (Ball et al. 1995) or *Agra* Fabricius (Casale 1998). Based on the study in the present work of all genera in Physoderina, we propose that Physoderina has more affinity with Calleidina than Metallicina. This is suggested by the following character states present in Physoderina and Calleidina but not in Metallicina: (1) apical segment of ovipositor without spine, usually spiculate, apex with extension usually membranous; (2) terminal labial palpomeres more or less widened in males; (3) mentum with tooth; (4) tempora ventrally without suborbital setigerous pore; and (5) males with terminal sternum emarginate.

So far, there is no rigorous phylogenetic analysis demonstrating that Physoderina is a monophyletic lineage in Lebiini. But, monophyly of this subtribe could be suggested by the following apomorphic character states: (1) setigerous pores present on 5th interval; (2) median lobe of aedeagus usually with apical orifice opened apically, internal sac usually with a long flagellum-like sclerite; (3) right paramere trifurcate; (4) spermatheca inserted on bursa copulatrix or joining of common oviduct and bursa copulatrix.

##### Genera included.

Chaudoir (1877) proposed the name Physodérides with insufficient definition, but ambiguously included *Allocota* Motschulsky, *Cryptobatis* Eschscholtz, *Aspasiola* Chaudoir and *Lachnoderma* Macleay. The original vernacular family-group name is available according to the Zoological Code of Nomenclature, and was first used in Latinized form by Bates (1883: 207) (Bouchard et al. 2011). Generally, Physoderina included seven genera (Jedlička 1963, Löbl and Smetana 2003, Lorenz 2005) before the present work.

In the present paper, we move three genera previously placed in Calleidina or Pericalina to Physoderina, propose two new generic synonyms, resurrect one generic name from synonymy, and describe one gen. n.. Hence, a total of ten genera is presently included in Physoderina: *Paraphaea* Bates, *Anchista* Nietner, *Metallanchista* gen. n., *Physodera* Eschscholtz, *Diamella* nom. n., *Allocota* Motschulsky, *Lachnoderma* Macleay, *Dasiosoma* Britton, *Orionella* Jedlička, and *Endynomena* Chaudoir.

The New World Cryptobatida group (*sensu* Erwin 2004) shares many characters with Physoderina from the Old World, such as: mandible widened, pronotum more or less angulate in middle, elytral disc depressed, and, above all, internal sac of male genitalia with flagellum-like sclerites. We propose that this group could be most closely related to Physoderina and perhaps should be included therein, similar to the concept including *Cryptobatis* in the original Physoderina (Chaudoir 1877). But, as the focus of the present work is the Old World fauna, we do not present a better subtribal arrangement for this New World group.

##### Distribution.

All of the ten genera have their center of distribution in the Oriental Region. One of them (*Dasiosoma*) has some African species; two genera (*Physodera*, *Lachnoderma*) have only a few Australian species; two species (*Paraphaea binotata*, *Endynomena pradieri*) have an Oriental origin, but are also widely distributed in the Pacific islands.

##### Key to genera of Physoderina

**Table:** 

1	Head completely glabrous; pronotum usually glabrous, rarely with disc sparsely pubescent	2
–	Head at least with some pubescence on vertex; pronotum densely and equally pubescent	6
2	Mandibles moderately widened, outer margin slightly arcuate (Fig. 149); mid-lateral setae of pronotum present, lateral margins with a few accessory setae restricted to front and hind angles; median lobe of aedeagus usually with long setae (Figs 67–73), if without, main flagellum of internal sac absent (Figs 74–75)	3
–	Mandibles strongly widened, outer margin semicircular (Fig. 150); mid-lateral setae of pronotum usually absent, if present, lateral margins with numerous accessory setae nearly reaching to middle area; median lobe of aedeagus without long setae, main flagellum of internal sac well developed (Figs 76, 79)	5
3	Umbilical pores of 9th interval placed in two rows, some pores adjacent to 8th stria, others in middle of the interval (Fig. 148); males with two pairs of setae on terminal sternum; median lobe of aedeagus with apical lamella longer, bent to dorsal side (Fig. 75)	*Metallanchista* gen. n.
–	Umbilical pores of 9th interval placed in one row, all adjacent to 8th stria (Fig. 147); males with one pair of setae on terminal sternum; median lobe of aedeagus with apical lamella shorter, not bent	4
4	Lateral margins of pronotum completely rounded at middle (Fig. 155); males with adhesive hairs on 1st metatarsomere; males with terminal sternum deeply emarginate (Fig. 143); median lobe of aedeagus twisted to left, internal sac with main flagellum developed and sinuous (Figs 67–69)	*Paraphaea* Bates
–	Lateral margins of pronotum slightly angulate at middle (Fig. 154); males without adhesive hairs on metatarsomeres; males with terminal sternum moderately emarginate (as in Fig. 145); median lobe of aedeagus not twisted, internal sac with main flagellum reduced (Figs 70–73)	*Anchista* Nietner (part)
5	Pronotum wide, PW/PL more than 1.4; protibiae with cleaning spur developed (as in Fig. 142); 5th interval with one or two large setigerous pores near base, if additional small pores present, all pores placed before middle; aedeagus with apical part of median lobe strongly bent to right side (Fig. 76)	*Physodera* Eschscholtz
–	Pronotum narrow, PW/PL less than 1.4; protibiae with cleaning spur more or less reduced (Figs 140, 141); 5th interval with four to ten setigerous pores, equally placed; aedeagus with apical part of median lobe slightly bent to right side (Figs 79–84)	*Allocota* Motschulsky
6	Posterior supraorbital setae distant from eyes, insertions more or less tumid, forming a pair of humps (Figs 13, 45–48); elytra with fine setae along striae and on odd intervals, even intervals glabrous; internal sac with main flagellum extraordinarily thick (Figs 77–78); apical part of trumpet-form expansion strongly expanded	*Diamella* nom. n.
–	Posterior supraorbital setae near eyes, insertions even, not forming humps; elytra equally pubescent on all intervals; internal sac with main flagellum fine or absent; if present, trumpet-form expansion at most with apical part moderately expanded	7
7	Mentum tooth bifid, with more than six setae; elytral striae indistinct, with very coarse punctures; mandibles with accessory setae on outer scrobe; labrum with long additional setae, as long as primary ones; internal sac with main flagellum projected beyond apical orifice (Fig. 85)	*Lachnoderma* Macleay
–	Mentum tooth simple, with two to four setae; elytral striae distinct, deep or shallow, at most with fine punctures; mandibles with outer scrobe glabrous, sometimes with a few fine setae along outer ridge; labrum without or with very fine additional setae, distinctly shorter than primary ones; internal sac with main flagellum at most reaching apical orifice, not projected (Figs 72, 74, 91–94)	8
8	Pronotum with basal foveae very deep, forming short grooves (Figs 24–26); elytral dorsal setigerous pores indistinct; ligula with four long setae at apex and a few short setae on dorsal surface; males with adhesive hairs reduced on all tarsomeres	*Dasiosoma* Britton
–	Pronotum with basal foveae shallow (Figs 7, 27–30); 5th interval with one or two distinct setigerous pores on base; ligula only with four long setae on apex; males with adhesive hairs distinct, at least on 1st, 2nd protarsomeres and 1st mesotarsomere	9
9	Mandibles moderately widened, outer margin slightly arcuate (Fig. 149); males with one pair of setae on terminal sternum; median lobe of aedeagus with apical part gradually narrowed, internal sac without main flagellum (Figs 72, 74)	*Anchista* Nietner (part)
–	Mandibles strongly widened, outer margin semicircular (Fig. 150); males with two pairs of setae on terminal sternum; median lobe of aedeagus with apical part equal in width, internal sac with main flagellum present but sometimes very short (Figs 91–94)	10
10	Elytral intervals slightly convex, striae distinct (Figs 27–29); 7th interval without setigerous pore; pronotal base weakly but distinctly lobed; males with adhesive hairs on first two protarsomeres only; median lobe of aedeagus with apical orifice opened apically, without seta around the orifice (Figs 91-93)	*Orionella* Jedlička
–	Elytral intervals flat, striae very shallow, indistinct in apical part (Fig. 30); 7th interval with some setigerous pores; pronotal base hardly lobed; males with adhesive hairs on first three protarsomeres; median lobe of aedeagus with apical orifice opened dorsally, with long setae around the orifice (Fig. 94)	*Endynomena* Chaudoir

#### Genus Paraphaea

Bates, 1873 status resurrected

http://species-id.net/wiki/Paraphaea

Paraphaea Bates 1873: 312; Chaudoir 1877: 236 (synonymized with *Anchista* Nietner); Jedlička 1963: 449 (in part, as a synonym of *Anchista* Nietner); Habu 1967: 137 (in part, as a synonym of *Anchista* Nietner); Habu 1982: 102 (in part, as a synonym of *Anchista* Nietner).

##### Type-species:

*Paraphaea signifera* Bates, 1873 [= *Paraphaea binotata* (Dejean)], by monotypy.

##### Diagnosis.

*Paraphaea* Bates can be readily distinguished from most genera of Physoderina except *Anchista* Nietner and *Metallanchista* gen. n. by the glabrous surface, moderately widened mandibles, and long setae around the aedeagal apical orifice.

Differences between *Anchista* and *Paraphaea* are: (1) pronotum lateral margins slightly angulate in middle in *Anchista*, but completely rounded in *Paraphaea*; (2) *Anchista* usually with distinct isodiametric microsculpture on the elytra, while *Paraphaea* has indistinct microsculpture; (3) in *Paraphaea*, males with adhesive hairs on the 1st metatarsomere, but such hairs absent in *Anchista*; (4) males with terminal sternum deeply emarginate in *Paraphaea*, but only shallowly emarginate in *Anchista*; (5) internal sac of aedeagus without main flagellum in *Anchista*, but main flagellum well developed and sinuous in *Paraphaea*; (6) in *Paraphaea*, the spermathecal gland inserted near the middle of the spermatheca, spermatheca more or less bent near the middle, but in *Anchista* the spermathecal gland inserted near the apex of spermatheca, spermatheca nearly straight.

Comparison between *Paraphaea* and *Metallanchista* gen. n. is presented in the diagnosis part under *Metallanchista* gen. n.

##### Generic characters.

Dorsal side generally reddish-brown to dark brown, sometimes with faint metallic reflections; elytra unicolored or bicolored. **Head** glabrous; eyes hemispherical and strongly prominent; tempora shorter than half of eyes length, abruptly narrowed behind eyes; vertex flat. Antennae reaching elytral base; 1st antennomere slightly narrowed at base, 3rd slightly longer than 4th. Labrum smooth, without secondary setae; mandibles moderately widened, outer margin nearly straight (Fig. 149), glabrous on outer scrobe and dorsal ridge; terminal maxillary palpomeres fusiform in males and females; terminal labial palpomeres strongly securiform and truncate apically in the males, narrower in the females; ligula with apex slightly projected, with four long setae; paraglossae membranous, not longer than ligula, adnate; mentum tooth simple, with two setae near base; submentum with two long setae; genae glabrous beneath eyes. **Pronotum** slightly wider than head, disc glabrous or sparsely pubescent; mid-lateral setae present; front angles more or less setose, hind angles generally with a few additional short setae; pronotal base briefly but distinctly lobed; lateral margins completely rounded in middle (Fig. 155), more or less sinuate before hind angles; hind angles sharp, rectangular or subrectangular. **Elytra** wide, apex truncate, sutural angles not projected, outer angles completely rounded; sides slightly depressed in anterior third, disc with an indistinct depression near apical two-fifths; intervals glabrous or only odd intervals with a few additional setae; umbilical pores of 9th interval placed in one row (Fig. 147); basal margination nearly complete; basal pores well developed; 3rd interval with two to four setigerous pores, 5th interval with base slightly widened, with one setigerous pore; 7th and 8th intervals slightly tumid near apex. **Ventral side** nearly glabrous; males with terminal sternum deeply emarginate (Fig. 143), with one pair of setae; females with apex of terminal sternum straight or slightly emarginate, with two pairs of setae. **Legs** short; protibiae with cleaning spur well developed, distant from inner margin; tarsi widened, 4th tarsomere bifid, claws pectinate; males with adhesive hairs well developed (two whole rows) on 1st to 3rd pro-, 1st to 2nd meso- and 1st metatarsomeres, rudimentary (two rows but very weakly present near apex) on 3rd mesotarsomere. **Male genitalia** with median lobe of aedeagus twisted to left; apical orifice opened dorsally, strongly setose along basal margin; internal sac with main flagellum more or less sinuous, nearly reaching apical orifice, trumpet-form expansion small and strongly bent; apical bursa strongly sclerotized; three additional small sclerotized pieces placed near apical third; secondary flagellum short and indistinct (Fig. 67). **Female genitalia.** Spermatheca tubular, with distinct ring-sculpture, inserted on bursa copulatrix; spermathecal gland slender and long, inserted near middle of spermatheca; spermatheca more or less bent near middle. Apical segment of ovipositor scimitar-shaped, curved to outer side, inner margin slightly angulate near apex; with fine setae near apex; apex with elongate membranous extension.

##### Distribution

**Map 1.**
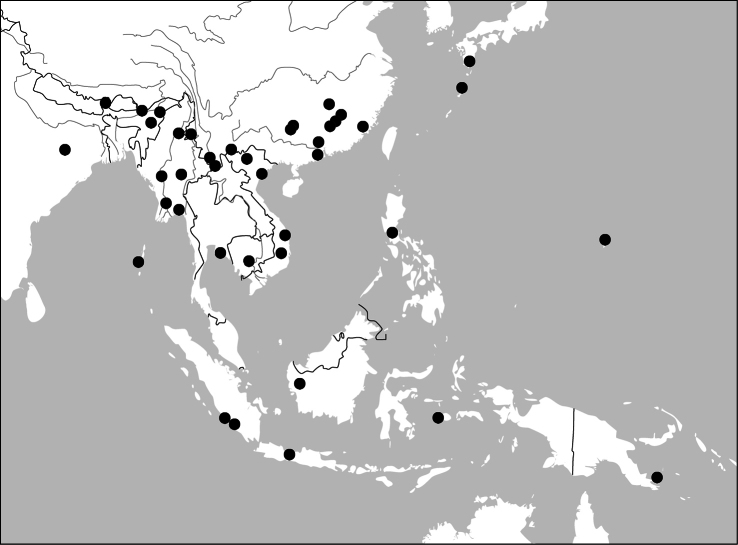
Known distribution of genus *Paraphaea* I: ● *Paraphaea binotata* (Dejean).

**Map 2.**
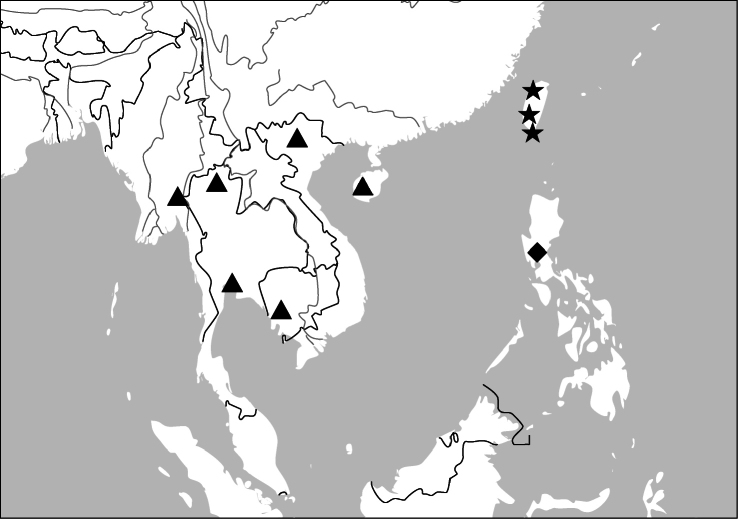
Known distribution of genus *Paraphaea* II: ▲ *Paraphaea minor* sp. n. ◆ *Paraphaea philippinensis* (Jedlička) ★ *Paraphaea formosana* (Jedlička).

(Maps 1, 2). This genus includes four species distributed in South and Southeast Asia. One of them (*Paraphaea binotata*) has a rather wide distribution, from Japan and India to the western Pacific islands (Map 1), but the other three species are more restricted.

##### Monophyly and relationships.

*Paraphaea* is presumed to be the sister group of *Anchista*. The relationship is supported by these character states: (1) mandibles moderately widened; (2) terminal sternum with single seta on each side in males; (3) median lobe of aedeagus strongly setose around apical orifice; (4) apical segment of ovipositor with inner margin slightly angulate near apex.

Monophyly of *Paraphaea* is suggested by the following apomorphic character states: (1) males with adhesive hairs present on hind tarsi; (2) males with terminal sternum deeply emarginate; (3) median lobe of aedeagus twisted; (4) spermatheca more or less bent near middle.

##### Taxonomic comments.

Bates (1873) proposed the genus *Paraphaea* without comparing it with *Anchista* Nietner. Later, Chaudoir (1877) synonymized these two genera without detailed explanation. Based on external characters, it is difficult to find significant differences between these two genera, but the genital and male secondary sexual characters mentioned above provide clear differences and justify the generic separation of *Paraphaea* Bates.

##### Key to species of *Paraphaea* Bates

**Table:** 

1	Pronotum more or less pubescent; pronotum reddish brown, elytra metallic blue; the Philippines (Fig. 36)	*Paraphaea philippinensis* (Jedlička)
–	Pronotum glabrous; elytra uniform brown or with bicolored pattern, not metallic	2
2	Third interval with more than three setigerous pores, 3rd 5th and 7th intervals with some secondary setigerous pores in some specimens; Taiwan (Figs 2, 34)	*Paraphaea formosana* (Jedlička)
–	Third interval with two setigerous pores, intervals without secondary setigerous pores	3
3	Pronotum widest at apical third, lateral margins slightly sinuate before hind angles; elytra with background dark, each side with an elongate pale patch before middle (Fig. 1); widely distributed in southeast Asia	*Paraphaea binotata* (Dejean)
–	Pronotum widest near middle, lateral margins distinctly sinuate before hind angles; elytra with disc yellowish, lateral margins and apex dark (Fig. 3); Hainan and Indo-China	*Paraphaea minor* sp. n.

#### Paraphaea binotata

(Dejean, 1825) comb. n.

http://species-id.net/wiki/Paraphaea_binotata

**Figures 1–6.**
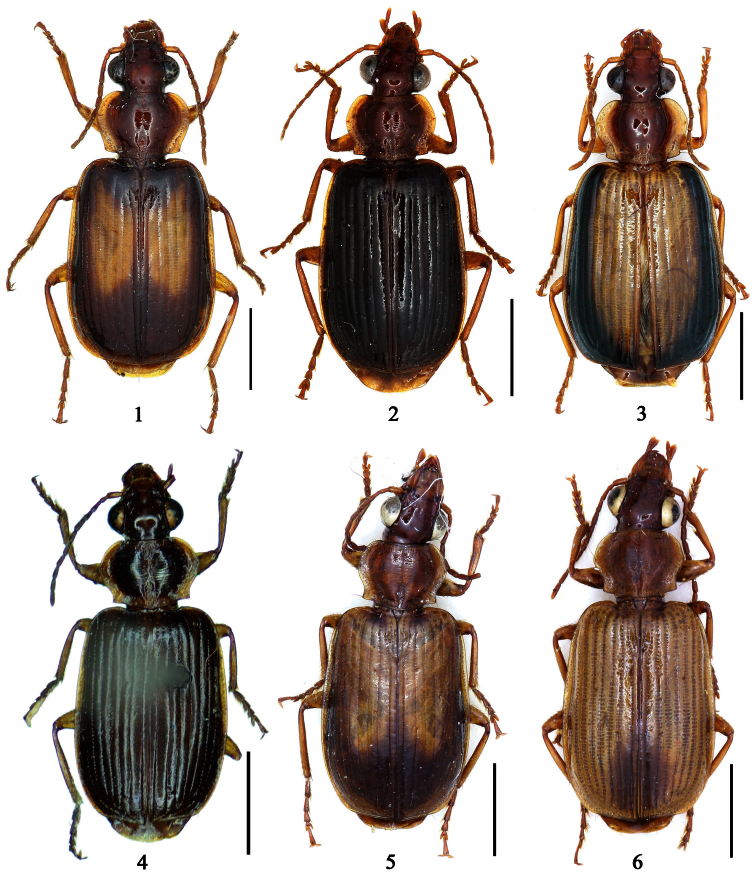
Habitus, scale bars = 2.0 mm: **1** *Paraphaea binotata* (Dejean), a specimen from Yunnan **2** *Paraphaea formosana* (Jedlička), a specimen from Taiwan **3** *Paraphaea minor* sp. n., a paratype from Hainan **4** *Anchista brunnea* (Wiedemann), a paralectotype of *picea* Chaudoir from India **5** *Anchista fenestrata fenestrata* (Schmidt-Göbel), a specimen from Nepal **6** *Anchista fenestrata fenestrata* (Schmidt-Göbel), a specimen from Nepal.

Habitus: Figs 1 31, 32, 33 male genitalia: Figs 67 95 female genitalia: Figs 111 126

Paraphaea binotata Dejean 1825: 252 (original: *Plochionus*; type locality: îles Mariannes; lectotype deposited in MNHN); Chaudoir 1877: 236 (*Anchista*; Indes orientales, Iles Andaman, Iles Mariannes, Japon); Bates 1883: 208 (*Anchista*); Bates 1889: 284 (*Anchista*; Qui-Nhon (Vietnam), Pnomh-Penh (Cambodia)); Bates 1892: 423 (*Anchista*; Bhamò (Burma)); Andrewes 1924b: 117 (*Anchista*; Siju Cave (Assam)); Andrewes 1930a: 337 (*Anchista*; Buru (Indonesia)); Andrewes 1930d: 22 (*Anchista*; catalogue); Kanô 1930a: 77 (*Anchista*); Csiki 1932: 1455 (*Anchista*, catalogue); Andrewes 1947: 12 (*Anchista*; Inle lake (Burma)); Jedlička 1963: 449 (*Anchista*; Japan, Philippinen, Süd-China, Birma, Indien, Andamanen); Habu 1967: 138 (*Anchista*; Japan); Darlington 1968: 140 (*Anchista*; Milne Bay (New Guinea)); Darlington 1970: 45 (*Anchista*; Saipan, Guam); Habu 1982: 103 (*Anchista*; Japan).
Calleida discophora Chaudoir 1852: 48 (type locality: nord de l’Hindostan; lectotype deposited in MNHN); Chaudoir 1877: 236 (*Anchista*; synonymized with *binotata* Dejean). [Synonym]
Paraphaea signifera Bates 1873: 312 (type locality: Satsuma (Japan); lectotype deposited in MNHN); Bates 1876: pl. I, Fig. 5; Chaudoir 1877: 236 (*Anchista*; synonymized with *binotata* Dejean). [Synonym]
Anchista eurydera Chaudoir 1877: 236 (type locality: Indes orientales; holotype deposited in MNHN); Andrewes 1930d: 22 (*Anchista*; catalogue); Csiki 1932: 1456 (*Anchista*; catalogue). **syn. n.** [Synonym]

##### Type examined.

**Lectotype** of *Plochionus binotatus* Dejean, designated herein (MNHN): male, body length = 7.8 mm, pin mounted, “TYPE” [red label]; “*binotatus, mil*. / *in inf. Mariannes*” [yellow label]; “*Guerin*” [yellow label]; “*Plochionus*”; “*Paraphaea* / *signifera Bates*”; “Ex Musaeo / Chaudoir” [red letters]; “Museum Paris / 1952 / Coll. R. Oberthür”; “LECTOTYPE ♂ / Plochionus binotatus / Dejean, 1825 / des. SHI H. L. 2011” [red label][Fig. 31]. **Lectotype** of *Calleida discophora* Chaudoir, designated herein (MNHN): male, body length = 8.0 mm; pin mounted; labia removed and separately pinned, “*Calleida* / *discophora Ch*.”; “Ex Musaeo / Chaudoir” [red letters]; “Museum Paris / 1952 / Coll. R. Oberthür”; “LECTOTYPE ♂ / Calleida discophora / Chaudoir, 1852 / des. SHI H. L. 2011” [red label][Fig. 32]. **Lectotype** of *Paraphaea signifera* Bates, designated herein (MNHN): male, body length = 7.9 mm; pin mounted, “TYPE” [red label]; “SATZUMA.”; “Ex. Musaeo / H. W. Bates, 1892”; “Museum Paris / 1952 / Coll. R. Oberthür”; “*Paraphaea* / *signifera* / *Bates*”; “LECTOTYPE ♂ / Paraphaea signifera / Bates, 1873 / des. SHI H. L. 2011” [red label][Fig. 33]. **Paralectotype** of *Paraphaea signifera* Bates (MNHN): a female, body length = 8.8 mm; pin mounted, labia removed and separately pinned, “SATZUMA.”; “PARATYPE” [red label]; “Ex. Musaeo /H. W. Bates / 1892”; “Museum Paris / 1952 / Coll. R. Oberthür”; “PARALECTOTYPE ♀ / Paraphaea signifera / Bates, 1873 / des. SHI H. L. 2011” [red label]. **Holotype** of *Anchista eurydera* Chaudoir, monotypy (MNHN): male, without head, pin mounted, “Ex Musaeo / Chaudoir” [red letter]; “TYPE” [red label]; “Museum Paris / 1952 / Coll. R. Oberthür”; “*eurydera* / *Chaudoir* / *Indes Orient*” [box label but pinned under specimen]; “HOLOTYPE ♂/ Anchista eurydera / Chaudoir 1877 / det. SHI H. L. 2011” [red label].

##### Notes on types.

***Plochionus binotatus* Dejean**: In the collection of MNHN, we found only one specimen (Fig. 31) from the Mariannas, apparently from Dejean’s collection. So this specimen could be the basis for the original description, although the original literature didn’t indicate or imply that the species was based on a single specimen. According to the Zoological Code of Nomenclature (4th Edition), Articles 61, 73 and 74, for taxonomic purpose of fixing the name to unique name-bearing type and preventing further uncertainty, we designate this specimen as the lectotype.

***Calleida discophora* Chaudoir**: The original literature didn’t indicate or imply how many specimens were examined. It can be confirmed that the male (Fig. 32) from Chaudoir’s collection, and bearing Chaudoir’s hand-written label, belongs to the type series. The other three specimens in Chaudoir’s collection do not accord with the original literature, although they are mentioned later (Chaudoir 1877). We herein designate this first specimen as lectotype for taxonomic purpose of fixing the name to unique name-bearing type.

***Paraphaea signifera* Bates**: This species was originally described from an unspecified number of specimens, but both sexes were mentioned, as well as type locality “Satzuma”. In the collection of MNHN, two specimens (one male and one female) from Bates’ collection are perfectly in accord with the original literature. We herein designate the male (Fig. 33) as lectotype for the purpose of providing the name with a unique name-bearing type. Another specimen from Bates’ collection with different locality was labeled as paratype by J. Mateu (not published), but it is neither a paratype nor a syntype and should not be part of the type series. We removed the paratype label.

***Anchista eurydera* Chaudoir**: This species was originally described from a single specimen which head is missing. This male in MNHN (ex. collection of Chaudoir) is clearly the holotype.

##### Non-type material examined

(total 141 specimens). **China**: 1 female (IZAS), “Jiangxi, Xingguo, 1956.7.26, Chen Yong leg., early season rice”. 1 female (IZAS), “Jiangxi, Xingguo, 1956.7.2, Chen Yong leg., early season rice”. 1 female (IZAS), “Jiangxi, Yongxin, 1956.5.12, Long Changqi leg., early season rice”. 1 male, 2 females (IZAS), “Jiangxi, Ganzhou, 1956.8.1, Qiu Futao leg., barley”. 1 female (IZAS), “Guangdong, Zhongshan, Cuihengcun, 1957.8.4, grassland”. 1 female (IZAS), “Guangdong, Yingde, Chengguan Granary, 1957.8.22”. 2 males (IZAS), “Guangdong, Zhongshan, Baqu, 1957.7.29”. 1 male (IZAS), “Guangdong, Nanxiong, 1956.7.5, Wu Guangwen leg., rice land”. 5 males 6 females (IZAS), “Guangxi, Guilin, Yanshan Mt., 1953.4.24/ 1953.5.4/ 1953.7.17”[Figs 67, 95]. 1 female (HBUM), “Guangxi, Guilin, Yanshan Mt., 2003.V.17, Li Tianshan leg.”[Fig. 126]. 7 males 4 females (IZAS), “Guangxi, Guilin, Liangfeng, 1952.3.13/ 1952.5.17”.[Fig. 111] 2 males 3 females (IZAS), “Guangxi, Lingui, 1952.3.6/ 1952.3.10/ 1952.3.13/ 1958.4.22”. 2 males (IZAS), “Guangxi, Lingui, Dayu, 1958.4”. 1 female (IZAS), “Yaoshan, 1938.V.25”. 1 female (IZAS), “Yangshuo, 1938.VIII.1”. 1 male (IZAS), “Yunnan, Jinping, 1958.6.19, black fungi”. 1 female (IZAS), “Yunnan, Luxi, 1957.5.20, Zhao Wenguang leg., rice land”. 1 female (IZAS), “Yunnan, Cheli, 1958.VIII.29”. 1 female (IZAS), “Yunnan, Xishuangbanna, Yunjinghong, 900m, 1958.VI.26, Zhang Yiran leg.”[Fig. 1]. 1 female (IZAS), “Yunnan, Xishuangbanna, Damenglong, 650m, 1958.VIII.7, Zhang Yiran leg.”. 3 specimens (MNHN), “Chine. Kouangsi, P. Barriere, 1909”. 1 specimen (MNHN), “Chine, Foo Kien, M. dela Touche, 1899”. **Japan**: 1 specimen (MNHN), “Japan“; “Ex. Musaeo, H. W. Bates, 1892”. **Vietnam**:2 males 4 females (IZAS), “Tonkin, Hoa-Binh, 1939.VII/ 1940.VII/ 1940.VIII, leg. A. de Cooman”. 1 specimen (MNHN), “Honoï, Domonge, 1909”. 1 specimen (MNHN), “Cochinchine, aui nhon”. 2 specimens (MNHN), “cochinchine, Hasmand, 1872”. 1 specimen (MNHN), “Cochinchine, Env. De Saigon, Simard, 1902”. 1 specimen (MNHN), “Cochinchine, Baudooin d’Aulne, 1897”. 2 specimens (MNHN), “Tonkin Sept., Ha-Lang, Lamey 151-97”. 37 specimens (MNHN), “Tonkin occ., Env. De Hoa-Binh, R. P. A. de Cooman, 1919.”. 9 specimens (MNHN), “Hoah binh, Tonkin”. **Thailand**: 2 specimens (MNHN), “Siam, Chantaboun, A. Battambang, A. Pavie, 1886”. **Cambodia**: 1 specimen (MNHN), “Cambodia“; “Ex. Musaeo, H. W. Bates, 1892”. 2 specimens (MNHN), “Cambodge, Rég de Chiehreng, G. Thomas 1912.”. 1 specimen (MNHN), “Kampong, Cambidge, Coll. J. Negre”. 1 specimen (MNHN), “Cambodge, 20/9/1912, R. Vitalis de Salvaza”. **Myanmar**:1 specimen (MNHN), “Bhamò, Birmania, Fea VII 1886”; “Ex. Musaeo, H. W. Bates, 1892”. 1 specimen (MNHN), “Minhia, Birmania, D. Comotto 1883”; “Ex. Musaeo, H. W. Bates, 1892”. 1 specimen (MNHN), “Birmanie, Theinzeik, P. Loizeau, 1914”. 1 male (MNPC), “Tharrawaddy, Burma, G. Q. Corbett”. **Indian:** 1 specimen (MNHN), “Ind. Or. Bor., Dr Bacon”; “Ex. Musaeo, Chaudoir”. 1 specimen (MNHN), “I. Andamman, H. Deyroll.”; “Ex. Musaeo, Chaudoir”. 1 specimen (MNHN), “Andaman”. 1 specimen (MNHN), “I. Andaman, Deyrolle 1877”. 1 specimen (MNHN), “Andaman, Coll. Borel”. 1 specimen (MNHN), “Chota Nagpore, Nowatoli, R. P. Cardon, XI-XII 1896”. 2 specimens (MNHN), “Naga Hills.”. 2 specimens (MNHN), “N. Manipur”. 1 specimen (MNHN), “Sikkim, Guntok, Eté 1894, Chasseurs Bretaudeau”. **The Philippines**: 1 specimen (MNHN), “Philipines, Ch. semper”. **Indonesia**: 1 specimen (MNHN), “Java, Mt. Tengger. Mme. E. Walsh”. 1 specimen (MNHN), “Sumatra, Rég. De. Benkoelen, Tandjong Sakti, Mme. M. E. Walsh, 1935”. 2 specimens (MNHN), “Paggar Alam, Sumara, J. Bouchard”. 1 specimen (MNHN), “Boreno Occ., Pontianak, 1903”.

##### Diagnosis.

Pronotum glabrous, widest at apical third, lateral margins slightly sinuate before hind angles; elytra with bicolored pattern, dark brown background with a reddish yellow elongate spot on each side (Fig. 1); elytra with two setigerous pores on 3rd interval, and one setigerous pore on base of 5th interval; elytra without any secondary setigerous pores; median lobe of aedeagus with apical third strongly expanded, internal sac with screwed main flagellum (Fig. 67).

The unique pronotal shape, elytral pattern and number of setigerous pores easily distinguish this species from all other allied species.

##### Description.

**Male genitalia**. Median lobe of aedeagus with apical third strongly expanded; in dorsal view, left-lateral margin strongly sinuate medially, and then gradually narrowed to apex; apical lamella placed on left-ventral side, broadly triangular, apex slightly rounded, not distinctly extended apically; internal sac with main flagellum screwed; basal part of main flagellum strongly bent, so trumpet-form expansion reaching right margin, apical margin of trumpet-form expansion crenulate (Fig. 67). **Female genitalia**. Spermatheca very long and slender; spermathecal gland inserted near apical one-third of spermatheca, with a short branch near base; spermatheca slightly expanded, with ring-sculpture between the gland insertion and apex, basal part of spermatheca without sculpture; spermatheca strongly bent at apical third (Fig. 126). Apical segment of ovipositor scimitar-shaped, inner margin slightly angulate at apical third; length about four times basal width; inner margin setose in apical half; apex sharp, with membranous extension long and slender (Fig. 111).

Detailed description of external characters has been provided by Habu (1967, 1982).

##### Distribution

(Map 1). China [Jiangxi, Guangdong, Guangxi, Yunnan, Taiwan (only Orchid Island, by Kanô 1930a)]; Japan; Vietnam; Thailand; Cambodia; Myanmar; India; the Philippines; Indonesia; New Guinea (Darlington, 1968); Mariana.

##### Notes on synonym.

*Anchista eurydera* Chaudoir was described from Indes Orientales which overlaps the range of *Paraphaea binotata*. The head of the male holotype is missing and its abdomen was badly destroyed by dermestid beetles, so it is impossible to examine its genitalia. Chaudoir (1877) noted that this species is extremely close to *Paraphaea binotata*, and wrote of some differences between these two species which are correct according to the holotype. But the differences in pronotal shape and elytral pattern should be regarded as mere individual variation, which is ubiquitous in this common and widely-distributed species. The paler color and more distinct yellow stripes on the pronotum are due to immaturity of the holotype. The transverse wrinkles on the pronotum are possibly an artifact caused by abnormal emergence. So we herein synonymize *Anchista eurydera* Chaudoir with *Plochionus binotatus* Dejean.

##### Remarks.

In Japan, this species is only recorded in South Kyushu and Satsunan Islands. Kanô’s (1930b) record of *Paraphaea binotata* from Tokyo is presumably a misidentification of *Euplynes batesi* Harold (Habu 1967). Kanô (1930a) recorded *Paraphaea binotata* from Taiwan (Orchid Island). It is probable that this record is also based on a misidentification. Moreover, we have studied a large series of carabid specimens from Taiwan, but didn’t find this species. So, it seems likely that this species is not distributed in Taiwan.

#### Paraphaea formosana

(Jedlička, 1946) comb. n.

http://species-id.net/wiki/Paraphaea_formosana

Habitus: Figs 2 34 male genitalia: Fig. 68

Parena formosana Jedlička 1946: 9 (original: *Parena*; type locality: Kuraru (Taiwan); holotype deposited in NMPC); Jedlička 1963: 450 (*Anchista*).

##### Type examined.

**Holotype** of *Parena formosana* Jedlička, by monotypy (NMPC): male, body length = 7.0 mm, board mounted, genitalia dissected and deposited in microvial pinned under specimen “*Kuraru* / Formosa. / *8-V.1935* / Col Y. Miwa”; “TYPUS” [red label with black frame]; “*formosana* / *sp. n*. / DET. ING. JEDLIČKA” [pink label][Fig. 34].

##### Notes on type.

Type locality “Kuraru” is the old spelling of “Kueitzuchiao”, in the area of “Kenting National Park” at the southernmost part of Taiwan.

##### Non-type material examined

(Total 10 specimens). 1 male (CCCC), “Taiwan, Pingtung County, Lilong Mt., 2008.XI.5 N, Chou Wen-I leg.” [Fig. 2]. 1 male (CCCC), “Taiwan, Pingtung County, Fenggang, Longfengsi, 2010.VIII.8 N, Lin Wensin leg.”. 1 male (MTMB), “Taiwan: Pingtung County, Kenting National Park, Hengchun Research Center, 21°57'43.0"N, 120°48'50.0"E, Lanyu plant collection, sweeping, 2008.VIII.18., leg. Redei D & Tsai JF”. 1 male (CCCC), “Taiwan, Taitung County, Haiduan, 1996.V.18, Chou Wen-I leg.”[Fig. 68]. 1 male (CCCC), “Taiwan, Taoyuan County, Fusing, Shangbaling, 1200m, 1994.VII.30, Chen Changchin leg.”. 1 female (MTMB), “Taiwan, Taipei county, PiHu, at light, 3.IV.2002, leg. Gy. Fabian & O. Merkl”. 1 female (NHML), “yauo. F. 31. FORMOSA MC:AR1 Abatsusen?. XII. 07 y. yauo”; “H. E. Andrewes Coll. B. M. 1945-97”. 1 female (NHML), “Mokuriryo, Near Mt. Ari, Formosa 1-IV. 1938 Coll. Yoshio Yano”; “H. E. Andrewes Coll. B. M. 1945–97”. 1 male (NHML), “Mokuriryo, Near Mt. Ari, Formosa 1-IV. 1938 Coll. Yoshio Yano”; “H. E. Andrewes Coll. B. M. 1945–97”. 1 female (NHML), “Formosa KAGI MT. TAIKOU 1937 Coll. Y. Yano.”; “27-12-1937 Coll. Yoshio Tano.”; “No. 626 YOSHIO YANO COLLECTION”; “H. E. Andrewes Coll. B. M. 1945–97”.

##### Diagnosis.

Pronotum glabrous, widest at middle, lateral margins sinuate before hind angles; elytra dark brown without pattern, or disc with a faint reddish patch; elytra with three or four setigerous pores on 3rd interval and one setigerous pore on base of 5th interval, in some specimens odd intervals with a few secondary setae; median lobe of aedeagus with apical third slightly expanded, internal sac with sinuous main flagellum (Fig. 68).

This species is closest to *Paraphaea minor*. Distinguishing characters are provided in the diagnosis of *Paraphaea minor*.

##### Description.

Body length 7.0–7.8 mm; head and pronotum reddish brown to dark brown, lateral explanate areas of pronotum paler; mouthparts, antennae and legs yellowish brown or reddish brown; elytra with luster, uniformly dark brown or disc somewhat reddish, in some specimens with a faint reddish patch on central area, the patch at most reaching 6th interval; ventral side yellowish brown to dark brown. **Head** glabrous, without punctures or microsculpture. **Pronotum** cordiform, widest near middle; ratio PW/PL 1.31 to 1.40; pronotal base briefly but distinctly lobed; disc glabrous, microsculpture indistinct, without punctures; front angles with a few setae, sometimes abundant and long (in specimens with additional interval setae), or sparse and short, but always distinctly longer than in *Paraphaea binotata*; lateral margins completely rounded in middle, sinuate before hind angles; front angles wide and rounded, not projected; hind angles rectangular or nearly so, slightly pointed; disc moderately convex; basal foveae wide and even, with a few punctures. **Elytra** with striae distinct, finely punctate along striae; intervals slightly convex, without punctures or microsculpture; 3rd interval with three or four setigerous pores, the apical one placed about apical one-eighth, adjacent to 2nd stria, position of the other two or three pores variable, pore number and position variable even in same specimen; 5th interval with one setigerous pore near base, adjacent to 5th stria; in some specimens, the odd intervals with some secondary setae; 17-19 umbilical pores on 9th interval. **Male genitalia.** Median lobe of aedeagus with apical third slightly expanded; in dorsal view, left-lateral margin moderately sinuate medially, and then gradually narrowed to apex; apical lamella placed in left-ventral side, narrowly triangular, apex rounded, slightly prolonged; internal sac with main flagellum sinuous; basal part of main flagellum moderately bent, trumpet-form expansion adjacent to left margin, apical margin of trumpet-form expansion not crenulate (Fig. 68). Female genitalia not studied.

##### Distribution

(Map 2). This species is endemic in Taiwan.

##### Geographical variation.

This is a highly variable species, although not widely distributed. According to the eleven specimens examined, three forms exist on Taiwan island: (1) The typical form is found in the southernmost part of Taiwan (four specimens examined), with elytra uniformly dark brown or disc slightly reddish and intervals without secondary setae; (2) specimens from the Alishan Mountains (four specimens examined), with a faint reddish patch in the middle of the elytra; (3) specimens from the lowlands of Taiwan (three specimens examined from Taitung, Taoyuan and Taipei) with secondary setae on odd intervals, and setae on front angles of pronotum distinctly more abundant and longer than the other two forms. These three forms are different only in color and secondary setae. We dissected the holotype, two typical form from Pingtung and a lowland form from Taitung, and found no significant differences between their male genitalia. We didn’t study male genitalia of the Alishan form.

#### Paraphaea minor

Shi & Liang sp. n.

urn:lsid:zoobank.org:act:C0987D82-6F52-42F8-93A8-D105D2A8D0B1

http://species-id.net/wiki/Paraphaea_minor

Habitus: Figs 3 35 male genitalia: Figs 69 96 female genitalia: Figs 112 127

##### Type material.

**Holotype** (MNHN): male, body length = 5.8 mm, board mounted, genitalia dissected and deposited in microvial pinned under specimen, “Hoa-Binh / Tonkin / A. de Cooman”; “MUSEUM PARIS / Coll. Ch. ALLUAUD”; “HOLOTYPE ♂/ Paraphaea minor / new species / Des. SHI H. L. 2011” [red label][Figs 69, 96, 35]. **Paratypes (**Total 1 male, 6 females**)**: **Vietnam**: 1 female (MNHN), “*Tonkin* / *Dap-Can*”; “MUSEUM PARIS / Ex. Coll. M. MAINDRON / Coll. G. BABAULT 1930”; “*Anchista* /*circumdata* / *sp. n*.”; “PARATYPE ♀/ Paraphaea minor / new species / Des. SHI H. L. 2011” [red label]. **Thailand**: 1 female (MNHN), “MUSEUM PARIS / BANGKOK / LARNAUDIE 349-64”; a round yellow label probably meaning Oriental with backside written “*3kg* / *Gk*”; “PARATYPE ♀/ Paraphaea minor / new species / Des. SHI H. L. 2011” [red label]. 1 male (CRS), “Thailand bor. occ. / Pai / Soppong / 28.5-5.6. 1997 / Lgt. M. Snizek”; “PARATYPE ♂/ Paraphaea minor / new species / Des. SHI H. L. 2011” [red label]. **Myanmar**: 1 male (NHML), “*Toungoo*”; “*Anchista* / *sp. n*.”; “H. E. Andrewes Coll. / B. M. 1945-97”; “PARATYPE ♂/ Paraphaea minor / new species / Des. SHI H. L. 2011” [red label]. **Cambodia**: 1 female (MNHN), “Cambodge / Rég. De Chiehreng / G. Thomas 1912.”; “Muséum Paris / Coll. R. Oberthür / 1952”; “PARATYPE ♀/ Paraphaea minor / new species / Des. SHI H. L. 2011” [red label]. **China**: 1 female (HBUM), “2006-XI-15-16 / 海南昌江县尖峰岭 / 任国栋 / 河北大学博物馆” [2006-XI-15-16, Hainan, Changjiang, Jianfengling, Ren Guodong leg., Hebei University Museum]; “PARATYPE ♀/ Paraphaea minor / new species / Des. SHI H. L. 2011” [red label][Figs 112, 127]. 1 female (IZAS), “采集地：海南五指山 / 海拔 日期 2008-*IV-03* / 方法 灯诱 采集人：杨玉霞” [Hainan, Wuzhishan, 2008.IV.03. by light trap, Yang Yuxia leg.]; “PARATYPE ♀/ Paraphaea minor / new species / Des. SHI H. L. 2011” [red label][Fig. 3].

##### Diagnosis.

Pronotum glabrous, widest at middle, lateral margins sinuate before hind angles; elytra reddish yellow with dark lateral and apical margins; two setigerous pores present on 3rd interval and one setigerous pore on base of 5th interval; median lobe of aedeagus with apical third slightly expanded, internal sac with sinuous main flagellum (Fig. 69).

The new species is most similar to *Paraphaea formosana* in male genitalia. It differs as follows: (1) 3rd interval with two setigerous pores in *Paraphaea minor*, but with three or four pores in *Paraphaea formosana*; (2) elytral patterns of these two species are different; (3) *Paraphaea minor* with median lobe of aedeagus less expanded subapically, left-lateral margin only slightly sinuate in dorsal view (Fig. 69); *Paraphaea formosana* with left-lateral margin of aedeagal median lobe more distinctly sinuate in dorsal view (Fig. 68); (4) *Paraphaea minor* with apical lamella completely rounded, very short, apex wide; *Paraphaea formosana* with apical lamella somewhat triangular, much longer, apex narrower.

This new species maybe confused with *Paraphaea binotata*, but can be distinguished by different elytral pattern, and pronotum widest at middle, not at apical third.

##### Description.

Body length usually 5.8–6.9 mm (specimens from Hainan length 7.6–7.9 mm); head and pronotum reddish yellow to brown, lateral explanate areas of pronotum paler; mouthparts, antennae and legs yellowish brown or reddish brown; elytra reddish yellow, with dark marginal fascia occupying 7th–9th intervals, fascia conjoined on elytral apex but not on base, elytral lateral margins yellowish brown; ventral side yellowish brown to dark brown. **Head** glabrous, without punctures or microsculpture. **Pronotum** cordiform, widest near middle; ratio PW/PL 1.38 to 1.52; pronotal base briefly but distinctly lobed; disc glabrous, microsculpture indistinct, without punctures; front angles with a few short setae; lateral margins completely rounded in middle, sinuate before hind angles; front angles wide and round, not projected; hind angles sharp, rectangular or nearly so, slightly pointed; disc moderately convex; basal foveae wide and even, with a few fine punctures. **Elytra** with striae distinct, finely punctate; intervals slightly convex, without punctures or microsculpture; 3rd interval with two setigerous pores: basal one placed approximately basal one-third, adjacent to 3rd stria; apical one placed approximately apical one-eighth, adjacent to 2nd stria or in middle of interval; 5th interval with one setigerous pore near base, adjacent to 5th stria; 15-17 umbilical pores on 9th interval. **Male genitalia.** Median lobe of aedeagus with apical third slightly expanded; in dorsal view, left-lateral margin moderately sinuate medially, and then gradually narrowed to apex; apical lamella placed on left-ventral side, broadly rounded, not prolongate; internal sac with main flagellum slightly sinuous; basal part of main flagellum moderately bent, so trumpet-form expansion adjacent to left margin, apical margin of trumpet-form expansion not crenulate (Fig. 69). **Female genitalia**. Spermatheca slender and strongly bent near middle, but not so elongate as in *Paraphaea binotata*; spermathecal gland inserted near apical one-third of spermatheca, distinctly expanded at basal part, not bent; spermatheca with ring-sculpture between apex and the gland insertion; spermatheca strongly bent at apical one-third (Fig. 127). Apical segment of ovipositor scimitar-shaped, inner margin slightly angulate at apical one-third; length about three times basal width; inner margin setose in apical half; apex slightly widened, with membranous extension long and slender (Fig. 112).

##### Distribution

(Map 2). China (Hainan), Vietnam, Thailand, Myanmar, Cambodia.

##### Etymology.

This species is the smallest one of the genus, so we name it “*minor*”, a word from Latin meaning “small”.

##### Geographical variation.

Two females from Hainan (China) (Fig. 3) are distinctly larger than the other specimens from South East Asia (7.6–7.9 mm contrasting with 5.8–6.9 mm). But there is no other difference between them and other paratypes from Tonkin in external and female genitalia characters, so we include these two females in type series.

#### Paraphaea philippinensis

(Jedlička, 1935b) comb. n.

http://species-id.net/wiki/Paraphaea_philippinensis

Habitus: Fig. 36 female genitalia: Figs 113 128

Paraphaea philippinensis Jedlička 1935b: 116 (original: *Coptodera*; type locality: Philippinen; holotype deposited in NHML); Jedlička 1963: 306 (*Allocota*); Shpeley and Ball 1993: 129 (*Anchista*); Kirschenhofer 1996: 763-764 (*Allocota*).

##### Type examined.

Holotype of *Coptodera philippinensis* Jedlička, by monotypy (NHML): female, body length = 7.6 mm, board mounted, with antennae missing except scapes, “Type” [round label with red ringed]; “Philippine Is. / Coll. Bottcher. / B. M. 1929-201.”; “*217*”; “*Coptodera* / *philippinen- / type sis sp. n*. / Det. ING. JEDLIČKA” [pink label]; “loan from / Brit. Mus.”; “*Coptodera / philippi- /* ♂ *ensis* / det. *Jedl/ 97* / George E. Ball”; “*Anchista* / *philippinensis* / det. *Jedlicka* / D. Shpeley 19*93*”[Figs 36, 113, 128].

##### Notes on type.

Shpeley and Ball (1993) mentioned the holotype is a male, but actually it is a female (Fig. 36).

##### Diagnosis.

Pronotum sparsely pubescent, widest at apical two-fifths, lateral margins slightly sinuate before hind angles; head and pronotum yellowish to reddish brown, elytra metallic blue; elytra with two or three primary setigerous pores on 3rd interval and one pore on base of 5th interval, intervals with a few very fine secondary setigerous pores.

By the metallic blue elytra and sparse secondary setae on pronotum and elytra, this species can be readily distinguished from other allied species.

##### Description.

Body length 7.6 mm; head reddish brown, pronotum, mouthparts and legs yellowish brown, apices of mandibles darker; elytra metallic blue with 1st and 2nd intervals slightly reddish, lateral margins, elytral suture and epipleurae reddish brown; ventral side brownish. **Head** nearly glabrous (only two very fine accessory setae visible), without punctures or microsculpture. **Pronotum** cordiform, widest at apical two-fifths; ratio PW/PL 1.52; pronotal base weakly lobed; disc sparsely and finely setose, lateral explanate areas and area along median line glabrous, microsculpture indistinct, without punctures; front angles with a few setae, distinctly longer than in *Paraphaea binotata*; lateral margins completely rounded in the widest part, slightly sinuate before hind angles; front angles widened and rounded, not projected; hind angles obtuse; disc moderately convex; basal foveae wide and even, with a few punctures. **Elytra** with striae very shallow, almost completely composed of rows of fine punctures; intervals flat, without punctures or microsculpture, very fine and sparse accessory setae present on all intervals; 3rd interval with two or three primary setigerous pores (in the holotype, two pores on left elytron, three pores on right one), first one placed at basal one-fourth, adjacent to 3rd stria, the last one placed at apical one-eighth, adjacent to 2nd stria, the middle one, if present, just behind the basal one, at basal one-third approximately; 5th interval with one pore near base, adjacent to 5th stria; 17-18 umbilical pores in 9th interval. **Male genitalia** unknown. **Female genitalia**. Spermatheca not distinctly bent in middle; spermathecal gland inserted near basal one-third of spermatheca, its base slightly expanded, with a very short branch; apical part of spermatheca after the gland insertion with distinct ring-sculpture, slightly expanded to fusiform; basal part before the gland insertion without sculpture, slightly bent (Fig. 128). Apical segment of ovipositor scimitar-form, inner margin slightly angulate at apical fourth; length 4 times basal width; inner margin setose in apical fourth; apex narrow, with membranous extension long and slender (Fig. 113).

##### Distribution

(Map 2). Only known from the type locality, the Philippines.

##### Remarks.

This species is only known from the holotype. It was originally described in genus *Coptodera* and later moved to *Allocota* by Jedlička (1935b, 1963). Shpeley and Ball (1993) indicated that it is an *Anchista* species. Kirschenhofer (1996) overlooked Shpeley and Ball’s (1993) comments, and kept it in *Allocota*. Based on the pronotal shape and female genitalia characters, this species is most similar to *Paraphaea minor* sp. n., so we herein move it into *Paraphaea*.

#### Genus Anchista

Nietner, 1856

http://species-id.net/wiki/Anchista

Anchista Nietner 1856: 523; Nietner 1857: 374 (redundant publication); Chaudoir 1877: 236 (in part); Csiki 1932: 1455 (catalogue); Jedlička 1963: 449 (in part; key to species); Habu 1967: 137 (in part); Habu 1982: 102 (in part); Kirschenhofer 1994: 1006 (in part; key to species).

##### Type-species:

*Anchista modesta* Nietner, 1856 [= *Anchista brunnea* (Wiedemann)], by monotypy.

##### Diagnosis.

Mandibles moderately widened (Fig. 149); pronotum with lateral margins slightly angulate in middle (Fig. 154), mid-lateral setae present; elytral 5th interval with one to four setigerous pores near base; terminal sternum of males moderately emarginate, with one pair of setae; median lobe of aedeagus usually strongly setose around apical orifice, internal sac without flagellum.

This genus is most closely related to *Paraphaea* Bates and *Metallanchista* gen. n. Comparison of these genera is presented in the key to genera and in the diagnosis of *Paraphaea* and *Metallanchista*.

##### Generic characters.

Dorsal side generally reddish brown to dark brown; elytra unicolored or bicolored. **Head** glabrous or sparsely pubescent; eyes hemispherical and strongly prominent; tempora shorter than half length of eyes, strongly narrowed behind eyes; vertex flat. Antennae extended to elytral base; 1st antennomere gradually narrowed to base, 3rd slightly longer than 4th. Labrum smooth, without secondary setae; mandibles moderately widened, outer margin nearly straight (Fig. 149), glabrous on outer scrobe and dorsal ridge; terminal maxillary palpomeres fusiform in both sexes; terminal labial palpomeres strongly securiform, apex truncate in males, less widened in females; ligula with apex slightly projected, with four long setae; paraglossae membranous, not longer than ligula, adnate; mentum tooth simple, with two setae near base; submentum with two long setae; genae glabrous or sparsely pubescent beneath eyes. **Pronotum** slightly wider than head, disc glabrous or pubescent; mid-lateral setae present; front angles more or less setose, hind angles generally with a few additional short setae; pronotal base briefly but distinctly lobed; lateral margins slightly angulate in middle (Fig. 154), distinctly sinuate before hind angles; hind angles sharp, rectangular or nearly so. **Elytra** wide, apex truncate, sutural angles not projected, outer angles evenly rounded; laterally slightly depressed in basal one-third, disc with an indistinct depression near basal two-fifths; umbilical pores of 9th interval placed in one row (Fig. 147); basal margination nearly complete or only reaching to 3rd interval; basal pores well developed; 3rd interval with two to four primary setigerous pores, 5th interval with one to four primary setigerous pores near base; elytra glabrous or pubescent; 7th and 8th intervals slightly tumid near apex. **Ventral side** nearly glabrous; males with apex of terminal sternum moderately emarginate, with one pair of setae; females with apex of terminal sternum straight or slightly emarginate, with two pairs of setae. **Legs** short; protibiae with cleaning spur well developed, quite distant from inner margin; tarsi widened, 4th tarsomere bifid, claws pectinate; males with adhesive hairs well developed (two whole rows) on 1st to 3rd protarsomeres; well developed or rudimentary (two rows but very weakly present near apex) on first three mesotarsomeres; absent on metatarsomeres. **Male genitalia** with median lobe of aedeagus nearly straight, not twisted; apical orifice opened dorsally, basal margin of apical orifice setose or glabrous; internal sac without flagellum or apical bursa, with weakly sclerotized sclerites and spined areas on internal sac. **Female genitalia.** Spermatheca tubular, not bent, with indistinct ring-sculpture, inserted on bursa copulatrix; spermathecal gland slender and long, inserted near apex of spermatheca. Apical segment of ovipositor scimitar-form, curved to outer side, inner margin slightly angulate near apex; with fine fluff near apex; apex with elongate membranous extension.

##### Distribution

**Map 3.**
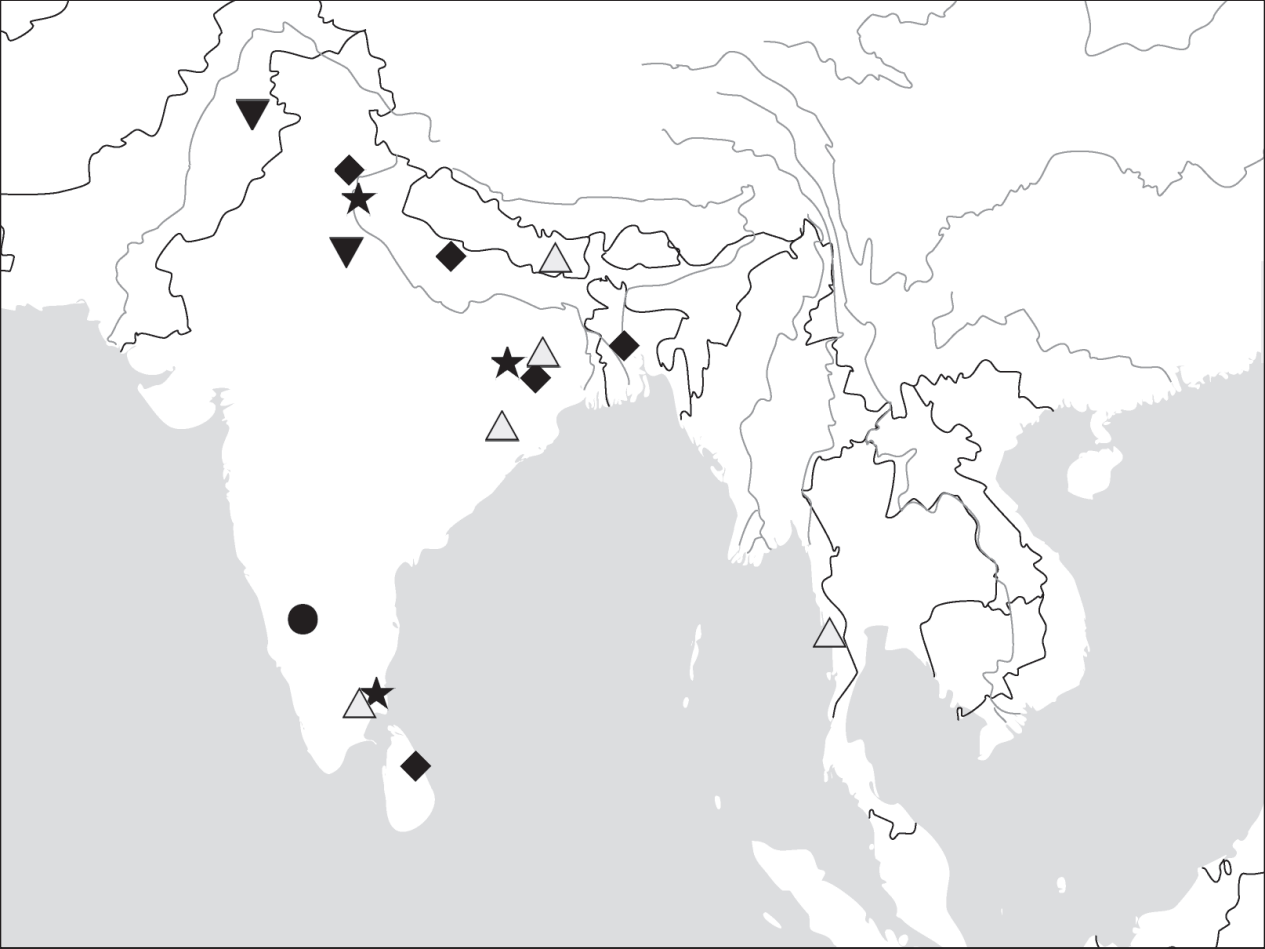
Known distribution of genus *Anchista*: ◆ *Anchista brunnea* (Wiedemann) ★ *Anchista nubila* Andrewes ● *Anchista pilosa* sp. n. △ *Anchista fenestrata fenestrata* (Schmidt-Göbel) ▼ *Anchista fenestrata subpubescens* Chaudoir.

(Map 3). This genus includes four species. *Anchista pilosa* is known only from the type locality (Bangalore, south India). The other three species are found in many localities in Indian Subcontinent, with *Anchista fenestrata* also occurring in Myanmar.

##### Monophyly and relationships.

The relationship between *Anchista* and *Paraphaea* is discussed under *Paraphaea*. The monophyly of *Anchista* is suggested by the following apomorphic character states: (1) pronotum slightly angulate in middle; (2) median lobe of aedeagus with main flagellum reduced, usually setose around apical orifice; (3) spermathecal gland inserted near apex of spermatheca.

##### Taxonomic comments.

Based on setose aedeagal apical orifice and mandibles moderately widened, *Anchista* could be closely related to *Paraphaea* Bates, although they have a different aedeagal internal sac, male secondary sexual character and distribution center. *Anchista*, together with *Metallanchista* gen. n., is unique in Physoderina in having an aedeagal internal sac without flagellum. The slightly sclerotized area near the median lobe base shows a reduced trumpet-form expansion (Fig. 71), so we believe these three genera are closely allied.

**Key to species of *Anchista* Nietner**

**Table:** 

1	Dorsal side evenly pubescent on most areas	2
–	Dorsal side glabrous	3
2	Basal margination of elytra only extended to 3rd interval; males with adhesive hairs well developed (two whole rows) on 1st mesotarsomere; median lobe of aedeagus with apical orifice glabrous. South India	*Anchista pilosa* sp. n.
–	Basal margination of elytra nearly complete; males with adhesive hairs rudimentary (weakly present near apex) on 1st mesotarsomere; median lobe of aedeagus with long setae around apical orifice; northern India and Pakistan	*Anchista fenestrata subpubescens* Chaudoir
3	Third interval with two setigerous pores; 5th interval with one setigerous pore near base	*Anchista brunnea*(Wiedemann)
–	Third interval with three or more setigerous pores; 5th interval with two or more setigerous pores near base	4
4	Pronotum distinctly angulate in middle; front angles with very short setae; elytra uniform reddish brown, sometimes with disc slightly paler, but not forming distinct pattern; males with adhesive hairs well developed (two whole rows) on 2nd and 3rd mesotarsomeres	*Anchista nubila* Andrewes
–	Pronotum slightly angulate in middle; front angles with long setae; elytra usually with distinct bicolored pattern; males with adhesive hairs rudimentary (weakly present near apex) on 2nd and 3rd mesotarsomeres	*Anchista fenestrata fenestrata* (Schmidt-Göbel)

#### Anchista brunnea

(Wiedemann, 1823)

http://species-id.net/wiki/Anchista_brunnea

Habitus: Figs 4 37, 38 male genitalia: Figs 70 97

Anchista brunnea Wiedemann 1823: 59 (original: *Lebia*; type locality: Bengalia; lectotype deposited in ZMUC); Andrewes 1921: 172 (*Anchista*); Andrewes 1930d: 22 (*Anchista*, catalogue); Csiki 1932: 1456 (*Anchista*, catalogue).
Anchista modesta Nietner 1856: 523 (type locality: Ceylon; syntype deposited in MZPW); Nietner 1857: 375 (*Anchista*, redundant publication); Chaudoir 1877: 239 (*Anchista*); Andrewes 1924a: 140 (*Anchista*, synonymized with *brunnea* Wiedemann); Andrewes 1927b: 103 (*Anchista*). [Synonym]
Anchista picea Chaudoir 1877: 238 (type locality: Deccan; lectotype deposited in MNHN); Andrewes 1921: 172 (*Anchista*, synonymized with *brunnea* Wiedemann). [Synonym]

##### Type examined.

**Lectotype** of *Lebia brunnea* Wiedemann, designated herein (ZMUC): female, body length = 7.4 mm, pin mounted, “Mus. / Westerm.”; “Type” [red label]; “*Bengal. / Mai 1809. / D: brunneus. /Lebia brun / nea Wied*. “; “LECTOTYPE ♀/ Lebia brunnea / Wiedemann, 1823 / Des. SHI H. L. 2011” [red label][Fig. 37]. **Lectotype** of *Anchista picea* Chaudoir, designated herein (MNHN), male, body length = 7.2 mm, pin mounted, “Ex Musaeo, Chaudoir” [red letter]; “TYPE” [red label]; “MUSEUM PARIS / 1952 / COLL. R. OBERTHÜR”; “*picea Chaudoir*, *Deccan*, *Stevens*” [large box label but pinned under specimen]; “LECTOTYPE ♂/ Anchista picea / Chaudoir, 1877 / Des. SHI H. L. 2011” [red label][Fig. 38]. **Paralectotype** of *Anchista picea* Chaudoir (MNHN): 4 males, “Ex Musaeo, Chaudoir” [red letter]; “PARATYPE” [red label]; “MUSEUM PARIS / 1952 / COLL. R. OBERTHÜR”; “PARALECTOTYPE ♂/ Anchista picea / Chaudoir, 1877 / Des. SHI H. L. 2011” [red label] [Figs 4, 70, 97].

##### Notes on types.

***Lebia brunnea* Wiedemann**: In the original literature, Wiedemann didn’t mention how many specimens belonged to the type series. We borrowed one female (Fig. 37) from ZMUC with a red label “Type” and Wiedemann’s hand-written label. We herein designate this specimen as lectotype for taxonomic purpose of fixing the name to unique name-bearing type.

***Anchista picea* Chaudoir**: Five specimens were mentioned in the original description. In the collection of MNHN, these specimens used to be in Chaudoir’s box, but were moved to Lebiini sorted boxes by J. Mateu. Obviously, Mateu selected the first one, and put Chaudoir’s box label and a red “type” label under it. We herein designate this specimen (Fig. 38) with such labels as lectotype for this species for taxonomic purpose of fixing the name to unique name-bearing type.

**Non-type material examined** (Total 7 specimens from India). 1 specimen (MNHN), “Chota Nagpore, Nowatoli, R. P. Cardon, XI-XII 1896”. 1 specimen (MNHN), “Chota Nagpore, Nowatoli, R. P. Cardon, VIII-IX 1896”. 2 specimens (NHML), “India Nevinson Coll. 1918–14.”. 1 specimen (NHML), “Fyzabad, Unt. Prov., India. R. W. G. Hingston.”; “Anchista brunnea Wied (= picea Chaud.), H. E. Andrewes det.”. 1 specimen (NHML), “Nagpur., C. P. India, 1000ft.”; “Anchista brunnea Wied, = picea Chaud., Compared with 2 types H. E. A.”. 1 female (NMPC), “Dabhalwala, Dehra Dun, India”.

##### Diagnosis.

Head and pronotum glabrous; pronotum widest at middle, lateral margins slightly angulate in middle; elytra uniform brownish; 3rd interval of elytra with two pores, 5th interval with one pore near base.

Based on the glabrous surface and number of setigerous pores on 3rd and 5th intervals, this species can be readily distinguished from all the other species of *Anchista*. Other species with the same pore number all belong to different genera (*Paraphaea*, *Metallanchista* gen. n.). They are different in elytral pattern and pronotum shape and have other generic differences.

##### Description.

Body length 7.2–7.8 mm; head and pronotum reddish brown; antennae and mouthparts yellowish brown or reddish brown, 1st antennomere and apices of terminal maxillary and labial palpomeres slightly paler; pronotum with lateral explanate area yellowish brown; elytra dark brown, without distinct pattern, but usually with basal two-thirds of disc slightly paler; ventral side same color as dorsal side; femora yellow, tarsi and tibiae yellowish brown. **Head** glabrous, without punctures or microsculpture. **Pronotum** glabrous,cordiform, widest slightly before middle; ratio PW/PL 1.38 to 1.41; pronotal base briefly but distinctly lobed; disc moderately convex, microsculpture indistinct, without punctures; front angles widened and rounded, not projected, with a few short setae; lateral margins slightly angulate in middle, strongly sinuate before hind angles; lateral explanate areas wide and even, with a few coarse punctures; hind angles sharp, rectangular or slightly acute, with a few very short accessory setae; basal foveae moderately deep, with a few punctures; median line distinct, strongly depressed and punctate anteriorly and subbasally. **Elytra** with striae shallow, with fine punctures; intervals hardly convex, without accessory setae, sparsely and finely punctate; microsculpture distinct, isodiametric; 3rd interval with two setigerous pores, basal one placed at basal one-third approximately, adjacent to 3rd interval, apical one placed at apical one-eighth approximately, adjacent to 2nd stria; 5th interval with one setigerous pore near base, adjacent to 5th stria; basal margination nearly complete. Males with adhesive hairs well developed (two whole rows) on 1st to 3rd mesotarsomeres. **Male genitalia** with median lobe of aedeagus straight and flat, slightly expanded to apex; left-lateral margin curved in middle; apical orifice opened dorsally, basal margin of apical orifice setose; apical lamella placed on right side, flat, short and rounded in dorsal view, slightly curved upward dorsally in lateral view; internal sac with two elongate and sinuate weakly sclerotized sclerites near apical orifice, basal part of internal sac finely scaled (Fig. 70). Female genitalia not studied.

##### Distribution

(Map 3). India, Bengal, Sri Lanka.

##### Notes on synonym.

Andrewes (1924a, 1927b) indicated that *Anchista modesta* Nietner is a synonym of *Lebia brunnea* Wiedemann. We didn’t examine the type of *Anchista modesta*, which should be in the Warsaw Museum, nor any material from Sri Lanka. We follow Andrewes’ treatment in the present paper.

#### Anchista fenestrata fenestrata

(Schmidt-Göbel, 1846)

http://species-id.net/wiki/Anchista_fenestrata_fenestrata

Habitus: Figs 5, 6 39, 40 male genitalia: Figs 71 96 female genitalia: Figs 114 129

Anchista fenestrata fenestrata Schmidt-Göbel 1846: 42 (original: *Plochionus*; type locality: Birma; holotype deposited in MNPC); Chaudoir 1872: 168 (excluded from *Plochionus*); Bates 1892: 424 (*Endynomena*); Andrewes 1923: 20 (*Anchista*); Andrewes 1930d: 23 (*Anchista*, catalogue); Csiki 1932: 1456 (*Anchista*, catalogue); Jedlička 1963: 449 (*Anchista*).
Anchista glabra Chaudoir 1877: 237 (type locality: Pondichéry; holotype deposited in MNHN); Andrewes 1930d: 23 (*Anchista*; catalogue); Csiki 1932: 1456 (*Anchista*, catalogue). **syn. n.** [Synonym]
Anchista nepalensis Kirschenhofer 1994: 1005 (type locality: Ostnepal; holotype deposited in NHMW). **syn. n.**

##### Type examined.

**Holotype** of *Plochionus fenestratus* Schmidt-Göbel, monotypy (NMPC): male, body length = 7.7 mm, board mounted, “MUS. PRAGENSE / COLL. HELFER”; “*Birma / Helfer*.”; “Typus! teste / Dr. J. Obenberger” [red label]; “*fenestratus* / *Sch. g*. /COL. HELFER”[Fig. 39]. **Holotype** of *Anchista glabra* Chaudoir, monotypy (MNHN): female, body length = 8.2 mm, pin mounted, “Ex Musaeo / Chaudoir” [red letter]; “TYPE” [red label]; “Museum Paris / 1952 / Coll. R. Oberthür”; “*glabra Chaudoir*/ *Coromandel* / *Pondichéry* / *Guerin*” [box label but pinned under specimen]; “HOLOTYPE ♀/ Anchista glabra / Chaudoir, 1877 / Des. SHI H. L. 2011” [red label][Fig. 40].

##### Notes on types.

***Plochionus fenestratus*** Schmidt-Göbel: One female was mentioned in the original description. In NMPC boxes of Schmidt-Göbel’s collection, we only found a male determined by him (Fig. 39). This male is the holotype, and Schmidt-Göbel determined the sex erroneously. In the label or original description, no precise locality for this specimen was mentioned. From the record of Helfer’s expedition (Reichardt 1880), it can be inferred that this specimen was collected by Helfer from Tenasserim in Burma (Myanmar).

***Anchista glabra***Chaudoir: Only one specimen was mentioned in the original description. Therefore the one in Chaudoir’s collection is the holotype (Fig. 40).

##### Non-type material examined 

(Total 25 specimens). **India**: 1 male and 1 female (MNHN), “S. India, Nedungadu, P. S. Nathan, 1936”. 1 male (MNHN), “Ramnad, Hindonstan”. 17 specimens (MNHN), “Chota Nagpore, Nowatoli, R. P. Cardon, XI-XII 1896”[Fig. 71]. 2 specimens (NHMB), “India: Orissa state, Similipal N. P. Lulung 21°56'N, 86°32'E, 25.v.–13.vi.1998, Karel & Simon Majer leg”. **Nepal**: 2 males and 1 female (NHMB), “E-Nepal, Koshi, M. Brancucci”; “Simraghat, 500m, 13.VI.85”[Figs 5, 6, 114, 129].

##### Diagnosis. 

Head and pronotum glabrous; pronotum widest at middle, lateral margins slightly angulate in middle; elytra distinctly bicolored; 3rd interval of elytra with three to five setigerous pores, 5th interval with two to four setigerous pores near base.

By the glabrous surface and number of setigerous pores on 3rd and 5th intervals, this species can be readily distinguished from most allied species, except *Anchista nubila*. Diagnosis between these two species is presented in the key.

Male genitalia characters show this species to be very similar to *Anchista brunnea*, but the latter has the apical lamella wider, internal sac without strongly scaled area near middle, and small sclerite near to the base. From the external characters, these two species can be readily distinguished by the different number of setigerous pores on 3rd and 5th intervals.

##### Description.

Body length 7.7–8.5 mm; dorsal side yellowish to reddish yellow, pronotum lateral explanate area paler; antennae yellowish brown to reddish brown, 1st antennomere slightly paler. Elytra with variable bicolored pattern: darker color brownish to black while the paler parts yellowish to reddish yellow; sometimes elytra mostly dark, but left lateral margin and outer part of apex paler, 2nd to 6th intervals with an elongate pale patch occupying basal three-fifths as in the holotype of *Anchista glabra* Chaudoir (Fig. 40); or elytra mostly pale, with a small ovaloid or sub-diamondoid dark patch near apex as in some specimens from Nepal (Fig. 6); but in most specimens, elytra with pattern between these two extremities (Fig. 5). Ventral side yellowish to reddish yellow; femora yellow, tarsi and tibiae yellowish brown. **Head** glabrous, without microsculpture, vertex with a few fine punctures. **Pronotum** glabrous, cordiform, widest slightly before middle; ratio PW/PL 1.41 to 1.51; pronotal base briefly but distinctly lobed; disc moderately convex, microsculpture indistinct, without punctures; front angles with a few moderately long setae; lateral margins slightly angulate in middle, strongly sinuate before hind angles; lateral explanate area wide and even, with a few coarse punctures; hind angles sharp, rectangular or slightly acute, with a few very short accessory setae; basal foveae moderately deep, with a few punctures; median line distinct, strongly depressed and punctate anteriorly and subbasally. **Elytra** with striae shallow but distinct, finely punctate; intervals slightly convex, without accessory setae, finely and sparsely punctate; microsculpture distinct, isodiametric; 3rd interval with three to five setigerous pores, the basal one usually placed at basal one-third approximately, adjacent to 3rd stria or on middle of interval, the rest adjacent to 2nd stria; 5th interval with two to four setigerous pores, the basal one near interval base, the last one usually at middle, all pores usually in center of 5th interval or slightly adjacent to 5th stria; basal margination nearly complete. Male with adhesive hairs rudimentary (two rows, weakly present near apex) on first three mesotarsomeres. **Male genitalia** with median lobe of aedeagus straight and flat, slightly expanded to apex; left lateral margin slightly curved in middle; apical orifice opened dorsally, basal margin of apical orifice setose; apical lamella placed on right side, flat, slightly elongate and rounded in dorsal view, slightly curved up dorsally in lateral view; internal sac with a small strongly sclerotized sclerite near base, two weakly sclerotized elongate and sinuate sclerites near apical orifice, a small and strongly scaled area present near middle of median lobe, internal sac in basal half sparsely and finely setose ventrally (Fig. 71). **Female genitalia**. Spermathecal gland inserted near apex of spermatheca, strongly expanded but not bent at base; spermatheca gradually expanded to apex, apex globular with a small papilla; ring-sculpture very faint, only present on subapical part of spermatheca (Fig. 129). Apical segment of ovipositor scimitar-form, inner margin gradually curved; length nearly three times basal width; inner margin setose in apical half; apex rounded and slightly projected to outer side, with membranous extension slightly widened.

##### Distribution

(Map 3). India, Myanmar, Nepal.

##### Notes on synonym.

We dissected males from Nedungadu (close to type locality of *Anchista glabra*), Chota Nagpore, Nepal Koshi (type locality of *Anchista nepalensis*), and the holotype of *Anchista fenestrata*. There is no significant difference among these specimens from different localities, except for elytral pattern variation. We therefore herein synonymize *Anchista glabra* and *Anchista nepalensis* with *Anchista fenestrata*.

##### Geographical variation.

In this species, specimens from one locality may be obviously different in elytral pattern. Sometimes, intrapopulational variation ranges even wider than interpopulational. But statistically interpopulational pattern variation exists: individuals from south India usually with dark pattern occupying more area on elytra (e. g. in the holotype of *Anchista glabra* Chaudoir, Fig. 40) than those from north India; while some specimens from Nepal have the paler color occupying the largest area, with only a small dark patch near apex (Fig. 6).

#### Anchista fenestrata subpubescens

Chaudoir, 1877 new rank

http://species-id.net/wiki/Anchista_fenestrata_subpubescens

**Figures 7–12.**
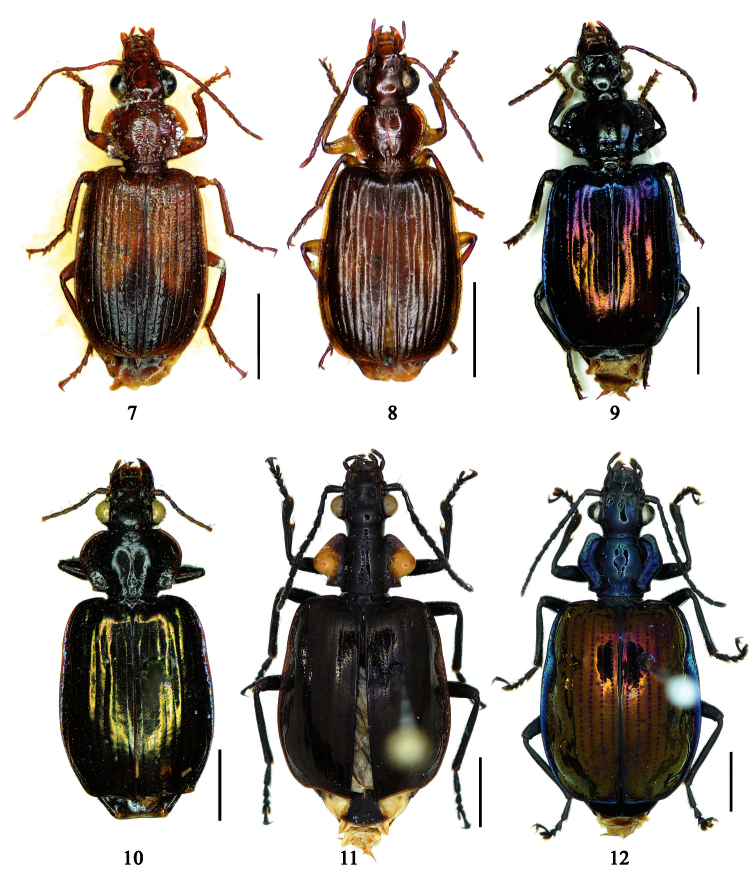
Habitus, scale bars = 2.0 mm: **7** *Anchista fenestrata subpubescens* Chaudoir, a specimen from Pakistan **8** *Anchista nubila* Andrewes, a specimen from India **9** *Metallanchista perlaeta* (Kirschenhofer), a specimen from Sumatra **10** *Metallanchista perlaeta* (Kirschenhofer), a specimen from Java **11** *Physodera dejeani* Eschscholtz, a specimen from Yunnan **12** *Physodera eschscholtzii* Parry, a specimen from Yunnan.

Habitus: Figs 7 41 male genitalia: Fig. 72

Anchista fenestrata subpubescens Chaudoir 1877: 238 (original: *Anchista*; type locality: nord de l’Hindostan; lectotype deposited in MNHN); Csiki 1932: 1456 (*Anchista*, catalogue).

##### Type examined.

**Lectotype** of *Anchista subpubescens* Chaudoir, designated herein (MNHN): female, body length = 7.8 mm, pin mounted, “Ex Musaeo / Chaudoir” [red letter]; “TYPE” [red label]; “*subpubescens* / *Chaudoir* / *Indes orient bor* / *Bacon*” [Chaudoir’s box label, but pinned under specimen]; “Museum Paris / 1952 / Coll. R. Oberthür”; “LECTOTYPE ♀/ Anchista subpubescens / Chaudoir, 1877 / Des. SHI H. L. 2011” [red label][Fig. 41].

##### Notes on types.

***Anchista subpubescens***Chaudoir: We found a female in the general Lebiini collection in MNHN, it was moved from the collection of Chaudoir. Except this one, we found no other specimen that accorded with the original literature in MNHN. The original literature didn’t indicate or imply this species was described on a single specimen, so we herein designate this specimen (Fig. 41) as lectotype for the purpose of fixing the name to unique name-bearing type.

##### Non-type material examined

(Total 33 specimens). **Pakistan**: 4 females (ZSM), “West Pakistan, Rawalpindi Umg., 25 km No., 600-, 700m, 20.XII.55, Chr. Lindemann leg.”. 1 female (ZSM), “West Pakistan, Rawalpindi Umg., Kanatti Chak, Salt Range, 9–14.I.56, Chr. Lindemann leg.”. 15 females, 8 males (ZSM), “West Pakistan, 11-, Rawalpindi Umg., Kanatti Chak, Salt Range, 15.II.56, Chr. Lindemann leg.” [4 of them with the label: “Anchista subpubescens, det. Ing Jedlička”][Figs 7, 72]. 3 females (NMPC), “West Pakistan 11.-, Rawalpindi Umg., Kanatti Chak, Salt Range 15.II.56, Chr. Lindemann leg.”. **India**: 2 females (CMB), “India, Rajasthan, Bharatpur 40km, w Agra N. P., Keoladeo leg., 11/07 Roppei”.

##### Diagnosis.

Dorsal side evenly pubescent; pronotum widest near middle, lateral margins slightly angulate; elytra bicolored; 3rd and 5th interval of elytra with primary pores small, indistinct, usually more than five pores on each interval.

##### Description.

Body size, form, and color the same as the nominotypical subspecies, also with similar elytral pattern variation. This subspecies differs from the nominotypical subspecies by: dorsal side finely and equally pubescent; microsculpture indistinct; head with pubescence sparse on vertex; elytra with intervals evenly pubescent; 3rd interval with five to eight setigerous pores, some pores small and indistinct, the basal one usually placed at basal one-fourth approximately, apical one adjacent to 2nd stria; 5th interval with five to seven setigerous pores at basal half, pores gradually decreasing to apex, sternum with dense pubescence. Male genitalia without significant difference from the nominotypical subspecies (Figs 71, 72).

##### Distribution

(Map 3). India (Rajasthan), Pakistan.

##### Remarks.

*Anchista subpubescens* Chaudoir was originally described from north India. According to specimens examined, it is distributed in northeast Pakistan and northwest India, allopatric with *Anchista fenestrata* from east India (Map 3). All specimens of *Anchista subpubescens* have no significant difference in male genitalia and external structure exceptdistinct dorsal pubescence. We therefore rank it as a subspecies of *Anchista fenestrata* (Schmidt-Göbel).

#### Anchista nubila

Andrewes, 1931

http://species-id.net/wiki/Anchista_nubila

Habitus: Figs 8 42 Habitus: male genitalia: Fig. 73

Anchista nubila Andrewes 1931: 526 (original: *Anchista*; type locality: Dehra Dun; lectotype deposited in NHML); Csiki 1932: 1456 (*Anchista*, catalogue).

##### Type examined.

**Lectotype** of *Anchista nubila* Andrewes, designated herein (NHML): female, body length = 8.5 mm, board mounted, “Dehra Dun / Rajpur, 3404 ft.”; “Bought from / Staudinger & / Bang-Haas, 1930”; “Type” [red label]; “*Anchista / nubila / Type Andr*. / H. E. Andrewes det.”; “H. E. Andrewes Coll. / B. M. 1945–97”; “LECTOTYPE ♀ / Anchista nubila / Andrewes, 1931 / des. SHI H. L. 2011” [Fig. 42]. **Paralectotypes** of *Anchista nubila* Andrewes: 1 male and 2 females (NHML), “Dehra Dun / Rajpur, 3404 ft.”; “Bought from / Staudinger & / Bang-Haas, 1930”; “Cotype” [round label, with green circle]; “H. E. Andrewes Coll. / B. M.1945–97”; “PARALECTOTYPE / Anchista nubila / Andrewes, 1931 / det. SHI H. L. 2011”.

##### Notes on types.

The original description mentioned five specimens from Dehra Dun in Andrewes’ collection, but did not designate a holotype. In the collection of NHML, we found only four specimens. Among them, a female specimen bears a determination label of Andrewes and a red label “Type”, and this is herein designated as lectotype (Fig. 42) for taxonomic purpose of fixing the name to unique name-bearing type. The other three specimens have only a round label “co-type”, but no Andrewes’ determination label.

##### Non-type material examined

(Total 16 specimens from India). 9 specimens (MNHN), “Chota Nagpore, Nowatoli, R. P. Cardon, VIII-XI 1896”[Fig. 73]. 3 specimens (MNHN), “Chota Nagpore, Nowatoli, R. P. Cardon, V.VI.1896”. 1 specimen (MNHN), “Inde Angl, Ghates, R. P. F. Tabourel, VII-IX 1898”. 1 specimen (MNHN), “S. India, Nedungadu, P. S. Nathan, 1936”[Fig. 8]. 1 specimen (MNHN), “Chota Nagpore”; “Ex Musaeo, H. W. Bates, 1892”. 2 specimens (NHML), “India mer. Tanjore Distr. Nedungadu”.

##### Diagnosis.

Head and pronotum glabrous; pronotum widest near middle, lateral margins distinctly angulate at middle; elytra uniform brownish; 3rd interval of elytra with three or four pores, 5th interval with two or three pores near base.

The glabrous surface and the number of setigerous pores on the 3rd and 5th intervals readily distinguish this species from most allied species, except *Anchista fenestrata fenestrata*. Diagnosis between these two species is presented in the key. In general appearance, this species looks like *Anchista brunnea*, but can be distinguished from the latter by the wider and more angulate pronotum, and the different number of elytral setigerous pores.

##### Description.

Body length 7.5–8.5 mm; dorsal side uniform reddish brown, pronotum lateral explanate areas somewhat paler; elytra usually with margins darker, but not forming central patch; antennae reddish brown, 1st antennomere paler; femora yellowish, tibiae and tarsi darker; ventral side reddish brown. **Head** glabrous, without or with very fine isodiametric microsculpture. **Pronotum** glabrous,cordiform, widest slightly before middle; ratio PW/PL 1.30 to 1.41; pronotal base briefly but distinctly lobed; disc moderately convex, microsculpture indistinct, without punctures; front angles with few short setae (distinctly shorter than in *Anchista fenestrata fenestrata*); lateral margins distinctly angulate in middle, strongly sinuate before hind angles; lateral explanate areas wide and flat, with few coarse punctures; hind angles sharp, rectangular or slightly acute, with few very short accessory setae; basal foveae moderately deep, with few punctures; median line distinct, strongly depressed and punctate anteriorly and subbasally. **Elytra** with striae shallow, distinct, finely punctate; intervals slightly convex, without accessory setae, finely and sparsely punctate; microsculpture distinct, isodiametric; 3rd interval usually with three setigerous pores, sometimes with four, all adjacent to 2nd interval, basal one usually placed at basal one-fourth approximately; 5th interval usually with two setigerous pores, sometimes with three, basal one near to interval base, all pores adjacent to 5th stria; basal margination nearly complete. Males with adhesive hairs rudimentary (two rows weakly present near apex) on 1st mesotarsomere, well developed (two whole rows) on 2nd and 3rd mesotarsomeres. **Male genitalia** with median lobe of aedeagus stout, bent to right side near apex; in lateral view, distinctly expanded in middle, gradually narrowed to apex; left lateral margin not curved in middle; apical orifice opened dorsally, basal margin of apical orifice with long setae; apical lamella placed on right side, flat, wide and rounded in dorsal view, not curved up dorsally; internal sac with a weakly sclerotized sclerite near middle, two weakly sclerotized elongate and sinuate sclerites near apical orifice, some parts of internal sac irregularly scaled (Fig. 73). Female genitalia not studied.

##### Distribution

(Map 3). India.

#### Anchista pilosa

Shi & Liang sp. n.

urn:lsid:zoobank.org:act:3BDFF1AA-FBD1-4699-B3C5-162BA0389D14

http://species-id.net/wiki/Anchista_pilosa

Habitus: Fig. 43 male genitalia: Figs 74 98

##### Type material.

**Holotype** (MNHN): male, body length = 7.8 mm, board mounted, genitalia dissected and deposited in microvial pinned under specimen, “Bangalore / Chikkangalur / Tabourel 1900”; “MUSEUM PARIS / 1952 / COLL. R. OBERTHÜR”; “HOLOTYPE ♂/ Anchista pilosa / new species / Des. SHI H. L. 2011” [red label][Figs 43, 74, 98].

##### Diagnosis.

Pronotum pubescent, lateral margins hardly angulate near middle, sinuate before hind angles; elytra with bicolored pattern; elytra pubescent, primary setigerous pores not distinct; elytral basal margination extended only to 3rd interval; median lobe of aedeagus without setae around apical orifice.

Based on pubescent surface and elytral pattern, the new species resembles the subspecies *Anchista fenestrata subpubescens* Chaudoir, but can be distinguished in having the following characters: (1) elytral basal margination extended only to 3rd interval, but in *Anchista fenestrata subpubescens* basal margination nearly complete; (2) pronotum with lateral margins less angulate and hind angles less distinct in the new species; (3) males with tarsi adhesive hairs well developed (two whole rows) on first three mesotarsomeres, but in *Anchista fenestrata subpubescens* adhesive hairs rudimentary (weakly present near apex) on first three mesotarsomeres in males; (4) males of the new species with terminal sternum more deeply emarginate than in *Anchista fenestrata subpubescens*; (5) median lobe of aedeagus without setae around apical orifice, but in *Anchista fenestrata subpubescens* median lobe of aedeagus with long setae around apical orifice.

##### Description.

Body length 7.8 mm; dorsum yellow to reddish yellow, pronotum lateral explanate areas pale yellow; 1st antennomere yellow, remaining antennomeres reddish brown. Elytra bicolored, forming faint pattern, background nearly brownish, with lateral part slightly paler; large yellow patch occupying inner five intervals from base to apical third; lateral margins and epipleura yellow. Ventral side yellow, sterna darker toward apex; femora and tibiae yellowish, tarsi yellowish brown. **Head** sparsely pubescent, nearly glabrous on front, without microsculpture or punctures. **Pronotum** with disc pubescent, rather sparser along median line, nearly glabrous on lateral explanate areas;pronotumcordiform, widest slightly before middle; ratio PW/PL 1.53; pronotal base briefly but distinctly lobed; disc moderately convex, microsculpture indistinct, without punctures; front angles with few moderately long setae; lateral margins hardly angulate in middle, strongly sinuate before hind angles; lateral explanate areas wide and even; hind angles slightly obtuse, with few accessory setae; basal foveae moderately deep, with few punctures; median line indistinct, slightly depressed subbasally. **Elytra** with striae shallow, distinct, slightly punctate along striae; intervals slightly convex, with dense and regular accessory setae; microsculpture indistinct; primary setigerous pores on 3rd and 5th intervals hardly visible, five pores on 3rd interval, two on the 5th; basal margination extended only to 3rd interval. Males with adhesive hairs well developed (two whole rows) on first three mesotarsomeres; males with terminal sternum more deeply emarginate than other members of the genus (as in Fig. 145), but less deeply than in *Paraphaea* (Fig. 143). **Male genitalia** with median lobe of aedeagus straight and flat, gently expanded to apex; left lateral margin slightly curved in middle; apical orifice opened dorsally, margin around apical orifice not setose; apical lamella placed on right side, flat and rounded, width subequal to length in dorsal view, not curved upwards dorsally in lateral view; internal sac with two moderately sclerotized flagella near apical orifice, internal sac irregularly scaled and sclerotized, basal part more distinctly sclerotized (Fig. 74). Female genitalia unknown.

##### Distribution

(Map 3). Only known from type locality Bangalore (India).

##### Etymology.

The name “*pilosa*” comes from Latin, meaning pubescent, referring to the pubescent surface of this species.

##### Remarks.

Based on median lobe of aedeagus without setae, elytral basal margination incomplete and male terminal sternum more deeply emarginate than other species, the new species seems to be isolated in the genus. But, male genital internal sac characters and distribution of male adhesive hairs on tarsi indicated that this species is a member of *Anchista*.

#### Genus Metallanchista

Shi & Liang gen. n.

urn:lsid:zoobank.org:act:5E1AFED8-B54F-4FDF-949A-F039F049D477

http://species-id.net/wiki/Metallanchista

##### Type-species:

*Metallanchista laticollis* Shi & Liang, sp. n.

##### Diagnosis.

Surface glabrous, with distinct metallic reflection; mandibles moderately widened (Fig. 149); umbilical pores of 9th interval placed in two rows (Fig. 148); males with terminal sternum deeply emarginate, bisetose on each side; males without adhesive hairs on metatarsomeres; median lobe of aedeagus without setae around apical orifice; internal sac with main flagellum reduced. These characters readily distinguish the new genus from all other genera of Physoderina.

The gen. n. is most closely allied to *Anchista*, but can be distinguished by: (1) median lobe with apical lamella longer and bent dorsally; (2) males with two pairs of setae on terminal sternum; (3) umbilical pores of 9th interval placed in two rows. *Metallanchista* new genus also resembles some species of *Physodera*, but differs by the following characters: (1) pronotum with mid-lateral setae; (2) basal margination of elytra nearly complete; (3) elytral intervals more or less convex; (4) 3rd to 5th intervals slightly depressed at basal two-fifths; (5) mandibles moderately widened.

##### Generic characters.

Body rather flat, dorsal side with distinct metallic reflections; microsculpture indistinct. **Head** glabrous; eyes hemispherical, prominent; tempora short, nearly half as long as eyes, abruptly narrowed behind eyes; vertex flat. Antennae hardly extended to elytral base; 1st antennomere stout, slightly expanded in middle, slightly narrowed to base, 3rd as long as 4th. Labrum smooth, without any secondary setae; mandibles moderately widened, outer margin nearly straight, glabrous on outer scrobe and dorsal ridge; terminal maxillary palpomeres fusiform in both sexes; terminal labial palpomeres moderately expanded but not strongly securiform in both sexes; ligula with apex nearly truncate, with four long setae; paraglossae membranous, slightly longer than ligula, adnate; mentum tooth wide and rounded, with two long setae near base, a few additional short setae present on central area of mentum; submentum with four long setae, distant from lateral margin; genae and gula with a few short seta at anterior part. **Pronotum** wider than head, disc glabrous; mid-lateral setae present; front angles more or less setose, hind angles generally with a few additional short setae; pronotal base briefly but distinctly lobed; lateral margins rounded or slightly angulate, strongly sinuate before hind angles; hind angles sharp. **Elytra** wide, apex truncate, sutural angles not projected, outer angles completely rounded; lateral margins slightly depressed in basal one-third, disc with an indistinct depression near basal two-fifths; intervals glabrous, without additional setae; umbilical pores of 9th interval placed in two rows (Fig. 148), the outer row (the primary umbilical pore series) adjacent to lateral expansion usually composed of 18 pores, the inner row (secondary umbilical pore series) adjacent to 8th stria usually composed of 10 pores; basal margination nearly complete; basal pores well developed; 3rd interval with two setigerous pores, basal one located at basal one-third approximately, adjacent to 3rd stria, apical one at apical one-eighth approximately, adjacent to 2nd stria; 5th interval slightly widened at base, with one setigerous pore near base, adjacent to 5th stria; 7th and 8th intervals slightly tumid near apex. **Ventral side** nearly glabrous; males with terminal sternum deeply emarginate apically (Fig. 143), bisetose on each side; females with terminal sternum straight apically, bisetose on each side. **Legs** short; protibiae with cleaning spur well developed, distant from inner margin; tarsi widened, 4th tarsomere bifid, claws pectinate; males with adhesive hairs well developed (two whole rows) on 1st to 3rd pro-, and 2nd to 3rd mesotarsomeres, rudimentary (two rows weakly present near apex) on 1st mesotarsomere. **Male genitalia** with median lobe of aedeagus not twisted; apical orifice opened dorsally, apical lamella without long setae; apical lamella strongly bent dorsally; internal sac with main flagellum fully reduced, without any distinct sclerite, some parts of internal sac strongly scaled. **Female genitalia.** Apical segment of ovipositor straight, glabrous; membranous extension short, slightly sclerotized. Internal reproductive system not studied.

##### Distribution

**Map 4.**
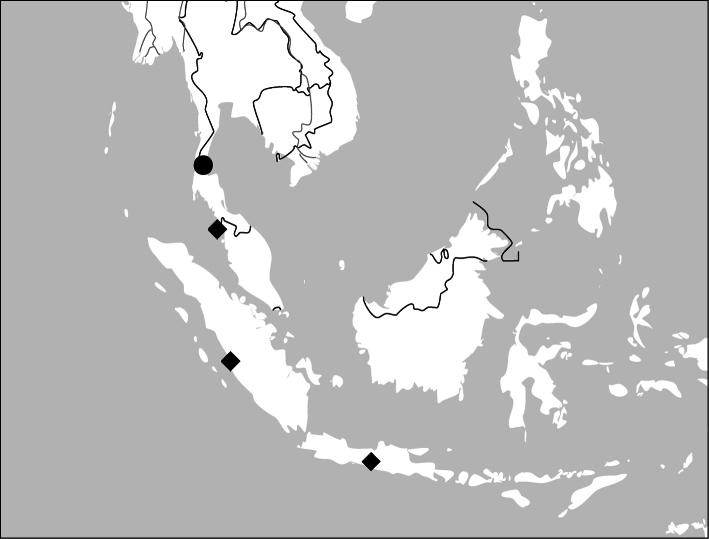
Known distribution of genus *Metallanchista*: ● *Metallanchista laticollis* sp. n. ◆ *Metallanchista perlaeta* (Kirschenhofer).

(Map 4). Malay Peninsula, Sumatra, Java.

##### Etymology.

The genus name “*Metallanchista*” is combined from the Greek “*metall*”, a reference to metal, and the genus name “*Anchista*”, meaning that it is closely related to genus *Anchista*, but strongly metallic. The gender of this genus name is feminine.

##### Monophyly and relationships.

The new genus is most closely allied with *Anchista*, as both genera have the main flagella of the aedeagal median lobe fully reduced. Both are also allied with *Paraphaea* and *Endynomena* in sharing: (1) median lobe of aedeagus usually strongly setose around apical orifice; (2) mandibles usually moderately widened; (3) basal margination of elytra usually complete. These character states may be synapomorphies for this lineage in the subtribe.

We establish this new genus based on the following apomorphic character states: (1) umbilical pores of 9th interval placed in two rows; (2) median lobe of aedeagus with apex strongly bent dorsally, internal sac without distinct sclerite, but scaled in certain areas; (3) submentum quadrisetose, ventral side of head with some accessory setae; (4) terminal sternum of males with two pairs of setae on each side.

##### Taxonomic comments.

So far as we know, there is no other genus of Lebiini with two rows of umbilical pores as in *Metallanchista* gen. n. The pore series in *Metallanchista* gen. n. contains more pores than in other allied genera, 28 pores contrasting with usually 15–20 pores in other genera. So the peculiar pore series is not the result of a different arrangement, but due to the presence of the unique secondary pore series adjacent to the 8th stria. The outer pore series, composed of 18 pores adjacent to the lateral expansion, is the primary pore series and homologous with the umbilical pore series in other genera.

##### Key to species of *Metallanchista* gen. n.

**Table:** 

1	Elytral disc metallic violaceous, green, or cupreous, lateral margins more or less bluish; pronotum narrower, width 1.50 times length, lateral margins completely rounded in middle (as in Fig. 155), front angles with some moderately long setae; elytral intervals barely convex	*Metallanchista perlaeta* (Kirschenhofer)
–	Elytra uniform metallic piceous–green; pronotum wider, width 1.65 times length, lateral margins slightly angulate in middle (as in Fig. 154), front angles with a few very short setae; elytral intervals slightly convex	*Metallanchista laticollis* sp. n.

#### Metallanchista laticollis

Shi & Liang sp. n.

urn:lsid:zoobank.org:act:2F0CFBB3-1150-43D5-95DC-C6F1DC39D141

http://species-id.net/wiki/Metallanchista_laticollis

Habitus: Fig. 44 male genitalia: Figs 75 99

##### Type material.

**Holotype** (NHMB): male, body length = 7.8 mm, board mounted, genitalia dissected and deposited in microvial pinned under specimen, “Thailand, 1.-20.iii.1996 / Chumphon prov. / Pha To env. 9°48’ 98°47’ / K. Majer leg.”; “HOLOTYPE ♂/ Metallanchista laticollis / new species / Des. SHI H. L. 2011” [red label][Figs 44, 75, 99].

##### Diagnosis.

Elytra uniform metallic piceous–green, intervals slightly convex; pronotal width 1.65 times length, lateral margins slightly angulate in middle, front angles with only a few very short setae. This species is similar to *Metallanchista perlaeta*, but can be distinguished from the latter by pronotal lateral margins angulate in middle and dorsal side with less metallic color.

##### Description.

Body length 7.8 mm; dorsum piceous, head and pronotum with moderate reflection, pronotum slightly brownish, elytra dark green, with strong metallic reflection; antennae, mouthparts yellowish brown, apices of mandibles darker, apices of terminal labial and maxillary palpomeres yellow; femora dark brown, tibiae and tarsomeres reddish brown; ventrum brown. **Head** glabrous, without microsculpture or punctures. **Pronotum** strongly transverse, widest slightly before middle; lateral explanate areas very wide and even, with a few punctures; ratio PW/PL 1.65; pronotal base briefly but distinctly lobed; disc moderately convex, microsculpture indistinct, without punctures or pubescence; front angles with a few very short setae; lateral margins slightly angulate in middle, strongly sinuate before hind angles; hind angles obtuse, sharp, not projected, with a few accessory setae; basal foveae shallow, with a few punctures; median line distinct, short, not reaching to base. **Elytra** with striae shallow, distinct, finely punctate; intervals slightly convex; microsculpture absent; 3rd interval with two setigerous pores, the basal one placed at basal one-third approximately, adjacent to 3rd stria, the apical one placed at apical one-eighth approximately, adjacent to 2nd stria; 5th interval with one setigerous pore near base. **Male genitalia** with median lobe of aedeagus tubular, slightly compressed; left lateral margin not curved, right lateral margin gently expanded in dorsal view; apical orifice opened dorsally, margin around apical orifice without long setae; apical lamella placed on right side, flat and elongate, apex rounded, length twice width; apical lamella strongly bent dorsally in lateral view; subapical area finely setose on right side. Internal sac without distinct sclerite; two separate areas near to dorsal margin strongly and coarsely scaled, basal one smaller and more coarsely scaled; main flagellum and trumpet-form expansion reduced to a hardly visible semi-sclerotized sclerite near base (Fig. 75). Female genitalia unknown.

##### Distribution

(Map 4). Only known from the type locality PhaTo (Thailand).

##### Etymology.

The name “*laticollis*” is from the Latin “*lat-*” meaning wide and “*coll*” meaning neck, referring to the pronotum. This species is named for its rather wide pronotum.

#### Metallanchista perlaeta

(Kirschenhofer, 1994) comb. n.

http://species-id.net/wiki/Metallanchista_perlaeta

Habitus: Figs 9, 10

Metallanchista perlaeta Kirschenhofer 1994: 1025 (original: *Allocota*; type locality: Langkawi Isld. (Malaysia); holotype deposited in NHMW).

##### Notes on types.

The holotype, a female, came from Langkawi Island. We have not examined it.

##### Non-type material examined

(Total 2 females from Indonesia) 1 female (CRS), “Sumatra C., Padang 3. 97, Panjang”[Fig. 9]. 1 female (NHML), “Java”; “Bowring., 63 47*”[Fig. 10].

##### Diagnosis.

Elytral color various, metallic violaceous, green, or cupreous, but always with distinct bluish color along lateral margins; pronotal width 1.50 times length, lateral margins completely rounded in middle, front angles with moderately long setae; elytral intervals hardly convex. This species is close to *Metallanchista laticollis* sp. n., but can be distinguished from it by different color and pronotal shape.

##### Description.

Body length 9.1–9.9 mm; head nearly black, with luster, antennae dark brown; mouthparts reddish brown, apices of terminal palpomeres yellow; pronotum black, with luster, slightly bluish in lateral expansion; elytra strongly metallic, disc violaceous, green, or cupreous, sometimes with basal and lateral areas bluish, but at least lateral margins distinctly bluish; epipleurae yellowish brown with metallic blue; ventral side dark brown to black, slightly lustrous; legs black, tarsomeres brown. **Head** glabrous, without microsculpture or punctures. **Pronotum** rounded, widest slightly before middle; lateral explanate areas very wide, flat, with a few punctures; ratio PW/PL 1.50; pronotal base briefly but distinctly lobed; disc moderately convex, microsculpture indistinct, without punctures or pubescence; front angles with some moderately long setae; lateral margins completely rounded in middle, strongly sinuate before hind angles; hind angles slightly acute, distinctly but slightly projected, with a few accessory setae; basal foveae shallow, with a few punctures; median line shallow, not reaching to apical or basal margin. **Elytra** with striae barely sulcate, composed of fine punctures, but slightly deepened in middle of 6th stria; intervals hardly convex, without punctures or microsculpture; 3rd interval with two setigerous pores, basal one placed at basal one-third approximately, adjacent to 3rd stria, apical one placed at apical one-eighth approximately, adjacent to 2nd stria; 5th interval with one setigerous pore near base. **Male genitalia** unknown. **Female genitalia** with apical segment of ovipositor slender and straight, without setae, length about four times basal width; widest at base, gradually narrowed to apex, apex sharp; inner and outer margin nearly straight; extended part on apex slightly sclerotized, very short. Spermathecal characters not studied.

##### Distribution

(Map 4). Malay Peninsula, Sumatra, Java.

##### Geographical variation.

So far only the three females of this species from different localities are known to us. They are different in color from each other, probably representing geographical variation. The holotype from Malay Peninsula has the elytra cupreous, apex violaceous, lateral margin slightly blue (by original description). The second female is from Sumatra and has the elytra violaceous on most areas, laterally with bluish band occupying 7th to 9th intervals, and the two bands joined at base. The last one is from Java and has elytra that are fully metallic green, but slightly bluish along lateral margin. More specimens, especially males, are needed for further study.

#### Genus Physodera

Eschscholtz, 1829

http://species-id.net/wiki/Physodera

Habitus: Figs 11, 12 male genitalia: Figs 76 100 female genitalia: Figs 115, 116 130, 131

Physodera dejeani Eschscholtz 1829: 8; Schmidt-Göbel 1846: 46; Lacordaire 1854: 130; Van de Poll 1889: 251; Heller 1923: 304 (key to species); Jedlička 1963: 300.
Nepalotarsus Mateu 1990: 156. Nomen nudum. [Synonym]

##### Type-species:

*Physodera dejeani* Eschscholtz, 1829, by monotypy.

##### Diagnosis.

This genus can be readily recognized by the combination of the following character states: dorsal side usually glabrous, at most elytra with a little secondary pubescence; mandibles strongly widened; pronotum wide, ratio PW/PL more than 1.4; primary mid-lateral setae of pronotum usually absent, if present, lateral margin with numerous accessory setae nearly along full length; protibiae with cleaning spur well developed; elytra with one or two setae near base of 5th interval; median lobe of aedeagus with apical orifice opened apically.

This genus is closest to *Allocota*, but can be distinguished in having: (1) pronotum and elytra much wider, ratio PW/PL more than 1.4 (usually more than 1.6, but narrower in *Physodera eschscholtzii* and allied species), but much narrower in *Allocota*, ratio PW/PL less than 1.4; (2) cleaning spur present on protibia (as in Fig. 142), but usually absent (Fig. 140) in *Allocota*, if present, very fine (Fig. 141); (3) 5th interval of elytra only with one or two primary pores near base, if additional small pores present, all pores placed before middle, but in *Allocota*, 5th interval of elytra with four to ten setigerous pores, equally distributed; (4) median lobe of aedeagus strongly bent to right side in dorsal view, but only slightly bent in *Allocota*; (5) apical segment of female ovipositor strongly elongate, but only a little longer than width in *Allocota*.

Some species of *Anchista*, *Paraphaea* or *Metallanchista* could be confused with members of this genus, but can be distinguished from *Physodera* by having the mandibles narrower, primary lateral seta of pronotum present and different shape of the aedeagus.

##### Generic characters.

Body length 8.0 to 12.5 mm, stout, pronotum usually wide, elytra wide and distinctly convex. Dorsal side usually metallic, usually with pattern composed of vivid color, or with ivory callosities on pronotum or elytra. Head and pronotum glabrous, elytra glabrous or with a few accessory setae; microsculpture indistinct. **Head** glabrous; eyes hemispherical, strongly prominent; tempora shorter or slightly longer than half length of eyes; tempora abruptly or gradually narrowed behind eyes; vertex flat. Antennae extended to basal one-fifth of elytra approximately; 1st antennomere slightly narrowed to base, 3rd slightly longer than 4th. Labrum smooth, without or with a few very short secondary setae; mandibles strongly widened, outer margins rounded, glabrous on outer scrobe, sometimes along dorsal ridge with a few fine and short setae; terminal maxillary palpomeres fusiform in both sexes; terminal labial palpomeres not widened in both sexes, or strongly securiform and truncate apically in males and less widened in females; ligula with apex truncate or slightly projected, with four long setae; paraglossae membranous, not longer than ligula, adnate; mentum tooth simple, apex rounded or truncate, with two setae or without seta near base; submentum with two long setae; genae glabrous beneath eyes. **Pronotum** distinctly wider than head, disc glabrous; front angles more or less setose, setae usually restricted to front angle, or nearly distributed along full length of lateral margins; mid-lateral seta usually absent; pronotal base more or less lobed; lateral margins usually completely rounded in middle; hind angles sharp or rounded. **Elytra** wide, strongly convex, apex truncate or slightly curved, sutural angles not projected, outer angles rounded; lateral margins more or less depressed at basal one-third, disc without distinct depression; intervals flat, glabrous or with a little pubescence or additional setae; basal margination reaching only to 3rd or 4th interval; basal pores well developed; primary setigerous pores small, 3rd interval with two to four setigerous pores, sometimes the apical one present on 2nd interval, 5th interval with one or two primary setigerous pores near base; 7th and 8th intervals slightly to strongly tumid near apex. **Ventral side** nearly glabrous; males with terminal sternum moderately emarginate, with one or two pairs of setae; females with terminal sternum apex straight or slightly curved, with two pairs of setae. **Legs** short; protibiae with cleaning spur well developed, distant from inner margin; tarsi widened, 4th tarsomere bifid, claws pectinate; males with adhesive hairs on 1st to 3rd protarsomeres well developed (two whole rows), those on 1st to 3rd mesotarsomeres rudimentary (two rows, weakly present near apex) or well developed (two whole rows) on some of these tarsomeres. **Male genitalia** with median lobe of aedeagus not twisted, strongly bent to right side in dorsal view; apical orifice opened apically; apical lamella usually long; dorsal surface with a few fine setae subapically; internal sac with main flagellum fine, apex nearly reaching to apical orifice, trumpet-form expansion usually small; apical bursa present or absent; secondary flagellum distinct or not. **Female genitalia.** Spermatheca tubular, with ring-sculpture, inserted at the joining of bursa copulatrix and common oviduct; spermathecal gland much longer than spermatheca, inserted near middle of spermatheca; spermatheca not distinctly bent. Apical segment of ovipositor tapered, apex pointed, base gradually widened, apex with membranous extension fine and long.

##### Distribution.

Oriental Region: India, Sri Lanka, Nepal, South China, Indochina Peninsula, Malay Peninsula, Malay Archipelago, New Guinea, the Philippines. Not discovered in Japan or Australia.

##### Monophyly and relationships.

*Physodera* appears to be closely related to *Allocota*, based on the similar aedeagal characters, generally glabrous surface, and pronotum usually without primary mid-lateral seta. Female internal reproductive system of *Physodera* resembles that of *Paraphaea philippinensis* (Jedlička), and may suggest a close relationship between these two genera.

Species of *Physodera* are more morphologically diverse than those in other genera of the subtribe. *Physodera dejeani* and related species seem to be more distant from other species of *Physodera*, according to the differences in the terminal labial palpomeres, setae on the terminal sternum and male genital characters. Further study may divide this genus into other subgenera or even genera.

Nevertheless, we suppose species of *Physodera*, or *Physodera* + *Allocota*, form a monophyletic lineage, such as those species previously arranged in *Anchista* (*Anchista* + *Paraphaea* + *Metallanchista* in the present paper). Monophyly of *Physodera* could be inferred by: (1) elytra strongly convex and wide; (2) pronotum strongly transverse; (3) terminal sternum or tergum usually with yellowish patch; (4) median lobe of aedeagus strongly bent to right side in dorsal view, apical lamella usually long.

##### Taxonomic comments.

Heller (1923) provided a key for the seven species of *Physodera* known at that time. Later, Andrewes (1930b, 1930c), Darlington (1971) and Mateu (1990) added some species to the genus. To date, including one new combination (*Lachnoderma andrewesi* Jedlička), a total of 15 available names has been included in *Physodera*, with one regarded as a junior synonym and one a subspecies. However, there is no comprehensive taxonomic work on this brilliant beetle group and such a work would be useful.

*Lachnoderma andrewesi* Jedlička was moved to *Allocota* by Jedlička (1963) and followed by Kirschenhofer (1996). But, from pronotal shape and elytral setigerous pores, it is far different from all other members of *Allocota*. After studying the female holotype, we confirmed that it is actually a *Physodera* species. The supporting characters are present in the diagnosis part of *Physodera*.

Mateu (1990) proposed the subgenus name *Nepalotarsus* for *Physodera bousqueti* Mateu and other species with adhesive hairs present on first three protarsomeres in males, but the type species of the subgenus was not fixed in the original publication. According to the Zoological Code of Nomenclature (4th Edition), Article 13.3, the name *Nepalotarsus* is a *nomen nudum*, an unavailable name.

**List of species:**

***Physodera amplicollis*** van de Poll, 1889: 254. Type locality: Java. Syntype location unknown.

***Physodera andrewesi*** (Jedlička, 1934: 120) (Original: *Lachnoderma*). Type locality: Mt. Makiling (Philippinens). Holotype deposited in NHML. **comb. n.**

***Physodera bacchusi*** Darlington, 1971: 326. Type locality: New Guinea. Holotype deposited in NHML.

***Physodera bifenestrata*** Heller, 1923: 304. Type locality: Sandakan (Borneo). Syntype deposited in SNSD.

***Physodera bousqueti*** Mateu, 1990: 156. Type locality: Nepal. Holotype deposited in BRIO.

***Physodera chalceres*** Andrewes, 1930b: 135. Type locality: Penang (Malaya). Holotype deposited in NHML.

***Physodera cyanipennis*** van de Poll, 1889: 253. Type locality: Celebes. Syntype location unknown.

***Physodera dejeani*** Eschscholtz, 1829: 8. Type locality: Manilla (Philippines). Syntype deposited in MNHN.

***Physodera diglena*** Andrewes, 1930c: 202. Type locality: Selangor (Malaya). Holotype deposited in NHML.

***Physodera eburata*** Heller, 1923: 304. Type locality: Los Banos (Luzon). Syntype deposited in SNSD.

***Physodera eschscholtzii*** Parry, 1849: 179. Type locality: Ceylan and Philippines. Syntypes deposited in NHML (probably) and MNHN.

***Physodera davidis*** Fairmaire, 1887: 92. Type locality: Fokien (China). Syntype deposited in MNHN.

***Physodera eschscholtzii sumatrensis*** (Kirschenhofer, 1996: 761) (Original: *Allocota*). Type locality: Sumatra. Holotype deposited in NHMW

***Physodera noctiluca*** Mohnike, 1875: 154. Type locality: Java. Syntype deposited in MNHN (probably).

***Physodera parvicollis*** van de Poll, 1889: 252. Type locality: Hong Kong. Syntype location unknown.

#### Genus Diamella

Shi & Liang nom. n.

http://species-id.net/wiki/Diamella

Diamella Jedlička 1952: 81; Jedlička 1963: 307. Junior homonym of *Diamella* Gude, 1913 (Hydrobiidae, Gastropoda). [Synonym]

##### Type-species:

*Diamella kaszabi* Jedlička, 1952, by monotypy.

##### Diagnosis.

Head and pronotum more or less pubescent; elytra with accessory setae at odd intervals or along striae, even intervals glabrous; vertex distinctly tumid, posterior supraorbital setae distant from eyes, insertions more or less pointed; internal sac of male genitalia with main flagellum extraordinarily thick, apical part of trumpet-form expansion strongly expanded.

In general appearance, this genus resembles *Allocota*, but the latter is different from *Diamella* in having: (1) head and pronotum always glabrous; (2) vertex less tumid and posterior supraorbital setae not distant from eyes; (3) lateral setae on pronotal margin absent; (4) males with terminal sternum bisetose on each side; (5) main flagellum of aedeagus finer.

##### Generic characters.

Dorsal side generally brownish to dark brown, elytra usually with metallic reflections; elytra unicolored. **Head** more or less pubescent, clypeus with some pubescence; eyes hemispherical, strongly prominent; tempora gradually narrowed behind eyes, longer than half length of eye; vertex distinctly tumid, posterior supraorbital setae distant from eyes, insertions more or less prominent, sometimes forming a horn-like hump. Antennae extended to basal one-sixth of elytra approximately; 1st antennomere slightly curved, 3rd slightly longer than 4th. Labrum with faint microsculpture, glabrous or with a few very fine short secondary setae, apex straight or emarginate; mandibles distinctly widened, outer margins rounded (as in Fig. 150), glabrous on outer scrobe, sometimes with a few short setae along dorsal ridge; terminal maxillary palpomeres fusiform in both sexes; terminal labial palpomeres strongly securiform, truncate apically in males, less widened in females; ligula with apex slightly projected, with four long setae; paraglossae membranous, not longer than ligula, narrow, adnate; mentum tooth simple, with two setae near base; submentum with two long setae; genae glabrous beneath eyes. **Pronotum** slightly wider than head, disc more or less pubescent; lateral margins with setae along full length, sometimes sparser at middle; primary mid-lateral setae present; pronotal base more or less lobed; lateral margins rounded in middle, sinuate before hind angles; hind angles sharp, rectangular or acute. **Elytra** wide, apex truncate, sutural angles not projected, outer angles completely rounded; anterior one third of sides slightly depressed or not, disc with or without a depression; odd intervals with accessory setae, even intervals glabrous; striae with or without setae; umbilical pores of 9th interval placed in one row; basal margination reaching to 3rd interval; basal pores present; primary setigerous pores indistinct among accessory ones, 3rd and 5th intervals with some larger pores; 7th and 8th intervals more or less tumid near apex. **Ventral side** with long and dense pubescence except proepisterna; males with apex of terminal sternum moderately emarginate, with 1one pair of setae; females with apex of terminal sternum straight or slightly curved, with two pairs of setae. **Legs** short; protibiae with cleaning spur well developed, distant from inner margin; tarsi widened, 4th tarsomere bifid, claws pectinate; males with adhesive hairs well developed (two whole rows) on 1st to 3rd protarsomeres, rudimentary (two rows, weakly present near apex) on 1st to 3rd mesotarsomeres. **Male genitalia** with median lobe of aedeagus not twisted; apical orifice opened apically; internal sac with main flagellum extraordinarily thick, apex nearly reaching to apical orifice, trumpet-form expansion strongly expanded; apical bursa absent; secondary flagellum short and indistinct. **Female genitalia.** Spermatheca very short, tubular, with distinct ring-sculpture, inserted on bursa copulatrix; spermathecal gland much longer than spermatheca, inserted distal to the middle of spermatheca; spermatheca indistinctly bent. Apical segment of ovipositor scimitar-shaped, curved to outer side; apical half setose; apex with elongate sclerotized extension.

##### Distribution

**Map 5.**
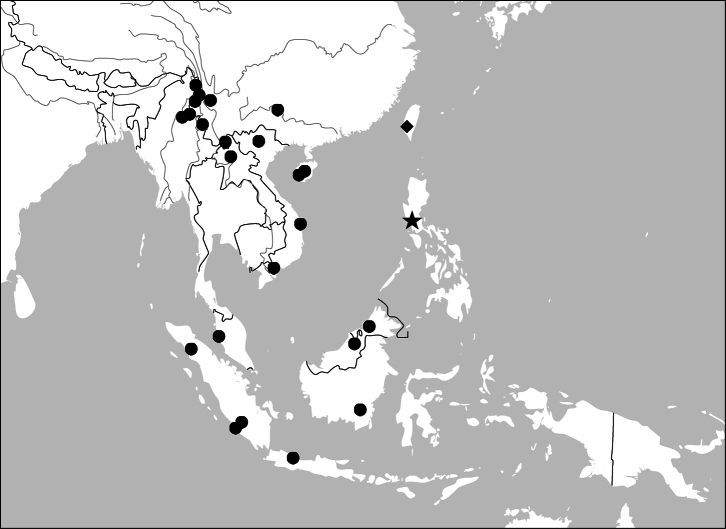
Known distribution of genus *Diamella*: ● *Diamella cupreomicans* (Oberthür) ◆ *Diamella kaszabi* (Jedlička) ★ *Diamella arrowi* (Jedlička).

(Map 5). South China, Indo-China Peninsula, Malay Peninsula, the Philippines, Borneo, Sumatra, Java. Not discovered in South Asia or islands east of Wallace’s Line.

##### Etymology.

Jedlička proposed the name *Diamella* for this genus. Unfortunately, it was preoccupied by a genus of snails in Hydrobiidae. We propose a new name “*Diamella*” for this genus herein, respecting Jedlička’s notion, with only one letter changed. The gender of this genus name is feminine.

##### Monophyly and relationships.

The nearest allied genus to *Diamella* is unclear. From general appearance and setigerous pores on odd intervals, this genus may be related to *Allocota*. Detailed discussion is presented in the taxonomic comments below.

Monophyly of *Diamella* is suggested by the following apomorphic character states: (1) vertex distinctly tumid, posterior supraorbital setae distant from eyes, insertions more or less pointed; (2) males with one pair of setae on terminal sternum; (3) internal sac of aedeagus with main flagellum extraordinarily thick; (4) apical segment of ovipositor with apical extension sclerotized.

##### Taxonomic comments.

The first described species of this genus, *Diamella cupreomicans* (Oberthür), has been placed in *Calleida*, *Physodera* and *Allocota* by different authors. Obviously, *Diamella cupreomicans* is much different from members of *Calleida* or *Physodera*. In general appearance, *Diamella cupreomicans* resembles some species of *Allocota*, but the concept of *Allocota* has never been clearly defined before the present work.

Among Physoderina, under the present system, *Diamella cupreomicans* could be more related to *Allocota* than any other genera both from external and genital characters (*Allocota* also has the main flagellum slightly thick). But *Diamella cupreomicans* should be excluded from *Allocota* based on two important characters: (1) males with one pair of setae on terminal sternum; (2) apical segment of ovipositor long and with apical extension sclerotized.

The mysterious species *Diamella kaszabi* has never been mentioned after Jedlička (1952, 1963). So far, it is only known from the unique female holotype. We examined the holotype and confirmed that this species is congeneric with *Diamella cupreomicans*. The most important characters shared by them are tumid vertex and uneven pubescence on elytral intervals. We therefore redefine the concept of genus *Diamella* herein to include three species.

##### Key to species of *Diamella* nom. n.

**Table:** 

1	Dorsal side uniform reddish brown, without metallic reflection; elytral base distinctly narrowed, hind wings reduced; elytral striae with punctures coarse; Taiwan	*Diamella kaszabi* (Jedlička)
–	At least elytra with distinct metallic reflection; elytral base wide, shoulder distinct, hind wings well developed; elytral striae with punctures fine; species from other localities	2
2	Vertex strongly tumid, sides of the tumidity forming a pair of horn-like humps, posterior supraorbital setae inserted on them; front angles of pronotum strongly narrowed; elytra with striae distinct, some setae inserted along striae; widely distributed in southeast Asia, but not occurring in Philippines	*Diamella cupreomicans* (Oberthür)
–	Vertex slightly tumid, not forming distinct horns, posterior supraorbital setae inserted on sides of the tumidity; front angles of pronotum widely rounded; elytra with striae barely sulcate, all setae on elytra inserted in middle of intervals; the Philippines	*Diamella arrowi* (Jedlička)

#### Diamella kaszabi

(Jedlička, 1952)

http://species-id.net/wiki/Diamella_kaszabi

Habitus: Fig. 45

Diamella kaszabi Jedlička 1952: 82, fig. (original: *Diamella*; type locality: Kosempo (Taiwan); holotype deposited in MTMB); Jedlička 1963: 307, Fig. 40 (*Diamella*).

##### Type examined.

**Holotype** of *Diamella kaszabi* Jedlička, by original designation (MTMB): female, body length = 8.1 mm, board mounted, “Formosa / Sauter”; “Kosempo / 908.”; “TYPUS” [red label]; “*Diamella* / *Kaszabi sp. n*. / det. ING. JEDLIČKA” [pink label][Fig. 45].

##### Notes on type.

The original literature clearly indicated that this unique specimen is holotype. The type locality “Kosempo” is old name for “Jiasianpu”, a small hill near township of Jiasian, Kaohsiung County. The locality = 23.07088°N, 120.59386°E, altitude about 380m.

##### Diagnosis.

This species can be readily distinguished from the other two species of the genus by: (1) dorsal side brownish, elytra without metallic reflection; (2) elytral base strongly narrowed, hind wings reduced; (3) striae with punctures very coarse. Furthermore, the species also differs from *Diamella cupreomicans* by: vertex less tumid, front angles of pronotum not narrowed; and from *Diamella arrowi* by: pronotum narrower, elytra with some setae along striae.

##### Description.

Body length 8.1 mm; dorsal side uniform brown, lateral explanate area of pronotum paler, pronotum and elytra with luster but not metallic; mouthparts and antennae reddish brown, apices of mandibles dark brown; legs yellowish brown; ventral side brownish. **Head** without microsculpture, with punctures and pubescence; tempora slightly shorter than length of eyes; vertex distinctly tumid, but not forming horn-like hump at posterior supraorbital seta insertion. **Pronotum** slightly wider than head, widest slightly before middle; ratio PW/PL 1.25; pronotal base strongly lobed; front angles not narrowed, slightly projected forward; lateral margins slightly expanded in middle, slightly rounded but not angulate, strongly sinuate before hind angles; hind angles subrectangular, distinctly projected; disc convex, microsculpture indistinct; lateral explanate areas wide and even; basal foveae slightly deep; disc nearly impunctate, with a few punctures along median line and in basal foveae; disc with a few long setae on basal and lateral areas, lateral explanate area glabrous; lateral margins with long accessory setae equidistant along full length; median line distinct, not reaching to apical and basal margins; disc slightly rugose beside median line. **Elytra** wider than pronotum, shoulder strongly narrowed, hind wings reduced, nearly as long as elytra; elytral lateral border wide, somewhat reflexed up; striae very deep, coarsely and irregularly punctate, with some setae, especially abundant on 5th to 7th striae; intervals convex, odd intervals with secondary pores, primary pores not distinct among the secondary pores, 3rd and 5th intervals with some larger pores; umbilical series of 9th interval composed of 22 pores; disc without distinct depression; 7th and 8th intervals slightly tumid near apex; elytral lateral margins with dense fine setae; epipleura pubescent. **Ventral side**. Prosternum with long hairs, proepisterna glabrous; mesosternum nearly glabrous; abdomen hairy. **Male genitalia** unknown. **Female genitalia** not studied.

##### Distribution

(Map 5). Only known from the type locality, Taiwan.

#### Diamella cupreomicans

(Oberthür, 1883) comb. n.

http://species-id.net/wiki/Diamella_cupreomicans

**Figures 13-18.**
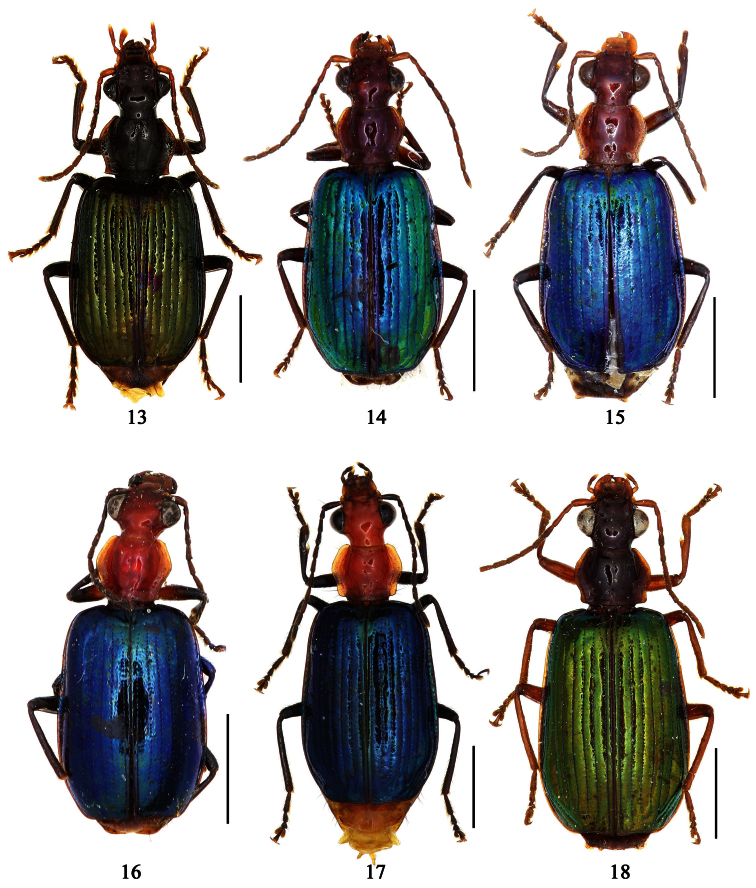
Habitus, scale bars = 2.0 mm: **13** *Diamella cupreomicans* (Oberthür), a specimen from Hainan **14** *Allocota viridipennis* Motschulsky, a specimen from Java **15** *Allocota viridipennis* Motschulsky, a specimen from Singapore **16** *Allocota cyanipennis* Heller, a specimen from Philippines **17** *Allocota bicolor* sp. n., a paratype from Hainan **18** *Allocota aurata* (Bates), a specimen from Japan.

Habitus: Figs 13 46, 47 male genitalia: Figs 77 101 female genitalia: Figs 117 132

Diamella cupreomicans Oberthür 1883: 218 (original: *Calleida*; type locality: Sumatra; lectotype deposited in NNML); Andrewes 1928: 590 (*Physodera*); Andrewes 1930d: 277 (*Physodera*; catalogue); Csiki 1932: 1347 (*Physodera*; catalogue); Andrewes 1936: 65 (*Physodera*); Kirschenhofer 1996: 763 (*Physodera*).
Allocota aerata Bates 1892: 425 (type locality: Birma: Bhamo; lectotype deposited in MSNG); Andrewes 1930d: 13 (*Allocota*; catalogue); Csiki 1932: 1348 (*Allocota*; catalogue); Andrewes 1936: 65 (*Physodera*, synonymized with *cupreomicans* Oberthür); Jedlička 1963: 306 (*Allocota*); Kirschenhofer 1994: 1027 (*Allocota*). [Synonym]

##### Type examined.

**Lectotype** of *Calleida cupreomicans* Oberthür, designated herein (NNML): male, body length = 6.9 mm, board mounted, “Dr. B. Hagen. / Tandjong Morawa. / Serdang / (N. O. Sumatra)”; “*type*” [green label]; “*Calleida / cupreomicans / R. Obr type*”; “Museum Leiden. / *Physodera / cupreomicans / Oberth /* Det:”; “type” [red label]; “LECTOTYPE ♂ / Calleida cupreomicans / Oberthür, 1883 / des. SHI H. L. 2011” [red label][Fig. 46]. **Paralectotype** of *Calleida cupreomicans* Oberthür: 1 female (NNML), “Dr. B. Hagen. / Tandjong Morawa. / Serdang / (N. O. Sumatra)”; “*Physodera/ cupreomicans / Oberth. /* H. E. Andrewes det.”; “Museum Leiden. / *Physodera / cupreomicans / Oberth /* Det:”; “*Cupreomicans / sp. n. R. Oberth*.”; “PARALECTOTYPE ♀ / Calleida cupreomicans / Oberthür, 1883 / det. SHI H. L. 2011” [red label]. **Paralectotype** of *Calleida cupreomicans* Oberthür: 1 male (MNHN), “Type” [red label]; “Dr. B. Hagen. / Tandjong Morawa. / Serdang / (N. O. Sumatra)”; “Museum Paris / 1952 / Coll. R. Oberthür”; “*Calleida* / *cupreomicans* / R. Oberthür, Type / *Notes Leyden Museum* / *Vol. V. 1883 p. 218*”; “PARALECTOTYPE ♂ / Calleida cupreomicans / Oberthür, 1883 / det. SHI H. L. 2011” [red label]. **Lectotype** of *Allocota aerata* Bates, designated herein (MSNG): female, body length = 7.5 mm, board mounted, “Bhamò / Birmania / Fea *VI* 188*5*”; “TYPUS” [red letter]; “*Allocota / aerata / Bates*”; “*aerata / Bates*”; “*Allocota / aerata / typus! Bates*” [yellow label]; “SYNTYPUS / *Allocota / aerata / Bates, 1892*” [red label]; “*Physodera / cupreomicans / Oberth. /* H. E. Andrewes det. *1933*”; “Museo Civico / di Genova”; “Museo Civico / di Genova”; “LECTOTYPE ♀ / Allocota aurata / Bates, 1892 / des. SHI H. L. 2011” [red label] [Fig. 47].

##### Notes on types.

***Calleida cupreomicans* Oberthür**. A total three specimens was mentioned in the original literature. We found one of them in MNHN, the general collection of Lebiini, and the other two in NNML. All of them have Oberthür’s determination labels, and agree with original description. We herein designate the male specimen (Fig. 46) in NNML as lectotype for taxonomic purpose of fixing the name to unique name-bearing type.

***Allocota aerata* Bates**. The original literature didn’t mention how many specimens were studied. We found only one female in MSNG with Bates’ determination and type label. It seems that this specimen is the only syntype. We herein designate this female as lectotype (Fig. 47) for taxonomic purpose of fixing the name to unique name-bearing type.

##### Non-type material examined

(Total 47 specimens). **China**: 1 female (HBUM), “Guangxi, Leye, Yachang Forestry Center, 2004.VII.24, Yu Yang & Gao Chao leg.”. 1 male (IZAS), “Yunnan, Fugong county, Lumadeng, Yaping, Yamuhe, 27.13561°N, 98.84166°E, 1612m, 2007.X.7, beating, Shi Hongliang & Liang Hongbin leg.”. 2 males (CAS), “Yunnan, Fugong county, Maji, Majimi, 27.40301°N, 98.82446°E, 1505m, 2005.VIII.26, beating, Liang Hongbin, Zhang Jinfeng leg.”. 1 specimen (IZAS), “Yunnan, Fugong county, Pihe, Wawa, 2005.VIII.24, Liang Hongbin leg.”. 2 females (IZAS), “Yunnan, Fugong county, Pihe, Wawa, 26.58548°N, 98.90467°E, 1120m, 2005.VIII.24, beating, Liang Hongbin, Zhang Jinfeng leg.”[Figs 117, 132]. 1 male (CAS), “Yunnan, Fugong county, Pihe, Bajiao, 26.54816°N, 98.89576°E, 1120m, 2005.VIII.23, beating, Liang Hongbin & Zhang Jinfeng leg.”. 1 female (IZAS), “Yunnan, Longyang, Mangkuan, Baihualing, 25.30367°N, 98.80031°E, 1625m, 2007.X.10, on shrubs, Liang Hongbin leg.”. 2 males (IZAS), “Yunnan, Tengchong county, Hehua, Nangyan, 24.94046°N, 98.38541°E, 1150m, 2006.VI.2, on vegetation, Liang Hongbin leg.”[Fig. 101]. 2 males, 1 female (CAS), “Yunnan, Tengchong county, Mangbang, Longwenqiao, 25.02396°N, 98.67675°E, 1285m, 2006.VI.5, on shrubs, D. Kavanaugh & R. Brett leg.”. 1 female (CAS), “Yunnan, Tengchong county, Qingshui, Rehai, 24.94919°N, 98.44921°E, 1450m, 2006.VI.1, on vegetation, D. Kavanaugh & R. Brett leg.”. 1 specimen (IZAS), “Yunnan, Tengchong county, Mangbang, Longwenqiao, 2006.VI.5, Beating, Liang Hongbin leg.”. 1 male (HBUM), “Yunnan, Yangbi county, Shunbi, 2004.V.21, Yang Xiujuan & Liu Yushuang leg.”. 1 female (IZAS), “Yunnan, Xishuangbanna, Menglun Botany Garden, 2009.XII.1, Tang G. leg.”. 1 female (HBUM), “Yunnan, Yingjiang county, 2004.V.17, Yang Xiujuan & Liu Yushuang leg.”. 2 males (HBUM), “Yunnan, Gengma county, Mengding, 2004.VIII.6-7, Li Jing, Yuan Caixia leg.”. 1 male (IZAS), “Yunnan, Xishuangbanna, Mengla, 620-650m, 1959.V.29, Zhang Yiran leg”. 1 female (IZAS), “Yunnan, Xishuangbanna, Damenglong, 650m, 1958.V.3, Hong Chunpei leg.”. 1 female (IZAS), “Hainan, Changjiang county, Bawangling, Est. 2 station, 19.09901°N, 109.17540°E, 1015m, 2007.V.9, light trap, Liang Hongbin leg.”. 2 males (IZAS), “Hainan, Baisha county, Nankai, 19.07926°N, 109.41133°E, 262m, 2009.XI.22, Liang Hongbin leg.”. 1 male (IZAS), “Hainan, Yinggeling, Hongkan Reservoir, 19.08121°N, 109.49839°E, 525m, 2009.XI.24, Liang Hongbin leg.”[Figs 13, 77]. **Vietnam**: 5 males 1 female (IZAS), “Tonkin, Hoa-Binh, 1939.VII, leg. A. de Cooman”. 1 female (IZAS)”Tonkin, Hoa-Binh, leg. A. de Cooman”. 1 female (MNHU), “Annam, Phuc-Son, Nov. Dez., H. Fruhstorfer”. 1 specimen (MNHN), “Tan Hoa, Indochine, VI-43”. **Laos**: 1 specimen (CMB), “Laos, 34 km nnw., Lanau Nam Pho, 5.5.2005, leg. Yokoi”. 1 specimen (NHML), “Laos, nam mia, 31-IV.1918, R. Vitalis de Salvaza”. **Malaya**: 1 specimen (NHML), “Malaya, Kuala Lumpur., at night, Jan. 9.1924”. **Borneo**: 1 specimen (MNHN), “Nord Borneo, Mont Kina Balu, 5-8-1903, John Waterstradt”. 1 female (NHML), “Sarawak:, foot of Mt. Dulit, Junction of rivers, Tinjar & Lejok.,25.ix,1932”. 1 female (NHML), “Pagat, Borneo, 8/82, Grabowth”. **Sumatra**: 1 specimen (NHML), “Sumatra, Manna 1901, M. Knappert.”. 3 specimens (MNHN), “Paggar Alam, Sumatra, J. Bouchard”. **Java**: 1 specimen (NHML), “Java merid., Palabuan 1892, H. Fruhstorfer”.

##### Diagnosis.

Vertex strongly tumid, forming horn-like humps at posterior supraorbital seta insertions; pronotal front angles strongly narrowed; elytral base wide, hind wings developed; elytra copperish to dark green, strongly metallic; elytra with some setae along striae. Comparison with the other two species of this genus is presented in the key above and diagnosis under those species.

##### Description.

Body length 6.9–8.0 mm; head and pronotum dark brown, lateral explanate areas of pronotum paler; elytra copperish to dark green, strongly metallic; mouthparts and antennae brown, apices of terminal labial palpomeres yellow; legs dark brown, with faint metallic reflection, tarsomeres brown; ventral side brownish with luster. **Head** without punctures or microsculpture, vertex with a few setae; tempora slightly shorter than length of eyes; vertex strongly tumid, forming horn-like humps at posterior supraorbital seta insertions, posterior supraorbital setae distant from eyes; labrum slightly widened to apex, apical margin straight or slightly curved. **Pronotum** slightly wider than head, widest at apical one-third; ratio PW/PL 1.27–1.35; pronotal base strongly lobed; front angles distinctly narrowed, not projected forward; lateral margins slightly expanded in middle, slightly rounded but not angulate, strongly sinuate before hind angles; hind angles acute or subrectangular, distinctly projected; disc convex, microsculpture indistinct; lateral explanate areas wide and even; basal foveae slightly deepened; disc nearly impunctate, with a few punctures only along median line and in basal foveae; disc and basal area with a few long setae, lateral explanate areas glabrous; lateral margins with long accessory setae equidistant along full length, sometimes sparser on middle area; median line distinct, not reaching apical or basal margins; disc slightly rugose beside median line. **Elytra** wider than pronotum, shoulder not narrowed, hind wings well developed; elytral lateral borders narrow, not reflexed up; striae distinct, coarsely and irregularly punctate; intervals slightly convex, odd intervals with secondary pores, primary setigerous pores indistinct among the secondary pores, only two pores distinguishable: one pore on 5th interval base adjacent to 5th stria and one apically on 3rd interval adjacent to 2nd stria; striae with some setae, more abundant in 5th to 7th striae; umbilical series of 9th interval composed of 17–18 pores; 3rd to 6th intervals slightly depressed subapically; 7th and 8th intervals distinctly tumid near apex; elytral lateral margins with dense fine setae; epipleura pubescent. **Ventral side**. Prosternum with long pubescence, proepisterna glabrous; mesosternum nearly glabrous; abdomen hairy. **Male genitalia** with median lobe of aedeagus straight and flat, gently expanded to apex; dorsal side slightly pubescent subapically; left lateral margin slightly curved in middle; apical orifice opened apically; apical lamella placed on right side, triangular, width subequal to length in dorsal view; internal sac with main flagellum thick and slightly sinuous, distinctly grooved ventrally, nearly reaching apical orifice; trumpet-form expansion strongly expanded, nearly half length of main flagellum (Fig. 77). **Female genitalia**. Spermatheca straight, short; apical fourth slightly widened, forming a weak globular expansion, with distinct ring-sculpture; subbasal part of spermatheca with faint ring-sculpture; spermathecal gland inserted near apical fourth of spermatheca, not branched, base slightly thickened and curved, slender and long, much longer than spermatheca (Fig. 132). Apical segment of ovipositor scimitar-shaped, slightly curved outwards after middle, gradually narrowed to apex; length four times basal width approximately; inner and outer margins finely setose in apical half; apex very sharp and fine, with very narrow and long sclerotized extension (Fig. 117).

##### Distribution

(Map 5). South China (Yunnan, Guangxi, Hainan); Myanmar, Laos, Vietnam, Malaysia, Indonesia (Sumatra, Borneo, Java).

##### Geographical variation.

Two females from Borneo differ from typical specimens by dark blue elytra, but other characters are identical with this species.

We dissected a paralectotype of *Calleida cupreomicans* Oberthür and a male from Yunnan, very close to the type locality of *Allocota aerata* Bates, and found that there were slight differences: specimen from Yunnan with median lobe of aedeagus a little more expanded to right side, and apical lamella slightly slenderer than that in the paralectotype of *Calleida cupreomicans*.

##### Notes on synonym.

After examining types of both species, Andrewes (1936) indicated that *Allocota aerata* Bates is a synonym of *Calleida cupreomicans* Oberthür. We also examined both types and confirmed the synonymy. Types of these two species come from far distant localities, but they are identical. The slight difference of genitalia between specimens is regarded as geographical variation.

#### Diamella arrowi

(Jedlička, 1935a) comb. n.

http://species-id.net/wiki/Diamella_arrowi

Habitus: Fig. 48 male genitalia: Figs 78 102

Diamella arrowi Jedlička 1935a: 15 (original: *Lachnoderma*; type locality: Philippines; holotype deposited in NHML); Jedlička 1963: 305 (*Allocota*); Kirschenhofer 1996: 764 (*Allocota*).

##### Type examined.

**Holotype** of *Lachnoderma arrowi* Jedlička, by original designation (NHML): male, body length = 7.3 mm, board mounted, “Philippine Is. / Coll. Bottcher. / B. M. 1929-201”; “*96*”; “Type” [round label with red ring]; “*Lachnoderma* / *Arrowi / Jedl. sp. n*. / det. ING. JEDLIČKA” [pink label] [Figs 48, 78, 102].

##### Notes on type.

The original literature clearly indicated that this specimen (Fig. 48) is the holotype. But precise locality was not provided.

##### Diagnosis.

Vertex moderately tumid, not forming horn-like humps at posterior supraorbital seta insertions; pronotum front angles not narrowed, lateral margins completely rounded in middle; elytra dark blue, strongly metallic; elytra with striae very shallow, with accessory setae only on odd intervals. Male genitalia of this species resemble *Diamella cupreomicans*, but: (1) apical lamella very short and wide, apex rounded; (2) in lateral view, median lobe of aedeagus slender, ventral margin less expanded near base.

##### Description.

Body length 7.3 mm; head and pronotum reddish brown, with luster but not metallic, lateral explanate areas of pronotum paler; elytra metallic blue with disc somewhat reddish brown; mouthparts and antennae reddish brown, apices of terminal labial palpomeres yellow; legs brown, tarsomeres slightly paler; ventral side brownish. **Head** without microsculpture or punctures, sparsely pubescent on vertex; tempora slightly longer than half length of eyes; vertex moderately tumid, posterior supraorbital setae distant from eyes, seta insertions slightly humped, but not horn-like; labrum slightly widened to apex, apical margin deeply emarginate. **Pronotum** slightly wider than head, widest slightly before middle; ratio PW/PL 1.45; pronotal base briefly but distinctly lobed; front angles wide; lateral margins strongly expanded in middle, completely rounded, slightly sinuate before hind angles; hind angles subrectangular, hardly projected; disc slightly convex, microsculpture indistinct; lateral explanate areas wide and even; basal foveae shallow; disc nearly impunctate, with a few punctures along median line and in basal foveae; disc with long setae except area along median line, some setae distributed to middle of lateral explanate areas; lateral margins with long accessory setae through their full lengths, sparser after middle; median line distinct, nearly reaching basal and apical margins; disc not rugose. **Elytra** wider than pronotum, shoulders not narrowed, hind wings well developed; elytral lateral borders narrow, not reflexed up; striae very shallow, finely punctate; scutellary intervals with three large pores (basal pores); intervals flat, without microsculpture, odd intervals with secondary pores, more abundant on 7th interval; primary pores indistinct among secondary pores, 3rd and 5th intervals with some larger pores distinguishable; setae not present along striae; umbilical series of 9th interval composed of 15 pores; disc without distinct depression; lateral margins slightly depressed at basal third; 7th and 8th intervals slightly tumid near apex; elytral lateral margins with dense and fine setae; epipleura pubescent. **Ventral side**. Prosternum with long pubescence, proepisterna glabrous; mesosternum nearly glabrous; abdomen hairy. **Male genitalia** with median lobe of aedeagus straight and flat, gently expanded to apex; dorsal side slightly pubescent subapically; left lateral margin slightly curved in middle; apical orifice opened apically; apical lamella placed on right side, short and rounded, distinctly wider than length in dorsal view; internal sac with main flagellum thick and slightly corkscrewed, distinctly grooved ventrally, nearly reaching apical orifice; trumpet-form expansion strongly expanded, nearly half length of main flagellum (Fig. 78). **Female genitalia** unknown.

##### Distribution

(Map 5). Only known from the type locality, the Philippines.

#### Genus Allocota

Motschulsky, 1859

http://species-id.net/wiki/Allocota

Allocota Motschulsky 1859: 29; Chaudoir 1877: 203; Jedlička 1963: 304 (in part); Kirschenhofer 1996: 763 (in part).
Taicona Bates 1873: 314; Jedlička 1963: 448; Habu 1967: 140. **Syn. n.** [Synonym]

##### Type-species:

*Allocota viridipennis* Motschulsky, 1859, by monotypy.

##### Diagnosis.

Dorsal side glabrous; mandibles strongly widened; posterior supraorbital setae near eyes, insertions not tumid; pronotum narrow, ratio PW/PL less than 1.4; lateral setae of pronotum absent; elytral 3rd and 5th intervals with four or more setigerous pores, subequally placed; 7th intervals sometimes with setigerous pores; protibiae with cleaning spur reduced or absent; males with two pairs of setigerous pores on terminal sternum.

This genus is closest to *Physodera*; their differences are presented in the diagnosis of *Physodera*. In general appearance, *Allocota* is similar to *Diamella*, but can be distinguished from the latter by the glabrous surface and vertex not tumid.

##### Generic characters.

Body length 6.0 to 8.7 mm, slender, pronotum narrow. Elytra strongly metallic, sometimes disc more or less reddish. Head and pronotum glabrous, elytra glabrous except primary setigerous pores; elytra usually with faint isodiametric microsculpture. **Head** glabrous; eyes hemispherical, strongly prominent; tempora slightly longer than half length of eyes, gradually narrowed behind eyes, not expanded; vertex not tumid. Antennae extended to about elytral basal one-third; 1st antennomere gradually narrowed to base, 3rd longer than 4th. Labrum smooth, without secondary setae, slightly widened to apex, apical margin more or less emarginate; mandibles strongly widened, outer margin rounded (Fig. 150), surface glabrous; terminal maxillary palpomeres fusiform in both sexes; terminal labial palpomeres more or less widened in males, fusiform, truncate or slightly securiform; less widened in females; ligular apex truncate, with four long setae; paraglossae membranous, as long as ligula, adnate; mentum tooth simple, apex rounded or sharp, with two setae near base; submentum with two long setae; genae glabrous beneath eyes. **Pronotum** slightly wider than long, nearly as wide as head; disc glabrous, front angles with a few setae, lateral margins glabrous; mid-lateral setae absent; pronotal base briefly but distinctly lobed; lateral margins slightly expanded in middle; hind angles more or less distinct; basal foveae shallow. **Elytra** narrow, slightly convex, slightly widened to apex; apex truncate or slightly curved, sutural angles not projected, outer angles rounded; disc without distinct depression; intervals flat, without additional pubescence; basal margination only reaching 3rd interval; basal pores present; 3rd and 5th intervals with four or more setigerous pores, sometimes 7th interval also with setigerous pores; 7th and 8th intervals strongly tumid near apex. **Ventral side** nearly glabrous; males with terminal sternum moderately emarginate apically, with two pairs of setae (Fig. 145); females with terminal sternum straight or slightly curved apically, with two pairs of setae, rarely one additional setae present on one side (the right side in Fig. 146). **Legs** short; protibiae with cleaning spur absent (Fig. 140), or very fine and short (Fig. 141), distant from inner margin; tarsi widened; 4th tarsomere bifid, claws pectinate; males with adhesive hairs on first three protarsomeres well developed (two whole rows), those on first three mesotarsomeres rudimentary (two rows, weakly present near apex) or well developed (two whole rows) on some mesotarsomeres. **Male genitalia** with median lobe of aedeagus not twisted, slightly bent to right side in dorsal view; apical orifice opened apically; apical lamella short; dorsal surface with some fine setae subapically; internal sac with main flagellum moderately thick, apex not reaching apical orifice, trumpet-form expansion small; apical bursa absent; secondary flagellum distinct. **Female genitalia.** Spermatheca tubular, with more or less ring-sculpture, inserted at the joining of the bursa copulatrix and common oviduct; spermathecal gland with basal part very fine, inserted at middle of spermatheca; spermatheca not distinctly bent. Apical segment of ovipositor very short and wide, apex rounded, with membranous extension slightly widened.

##### Distribution

**Map 6.**
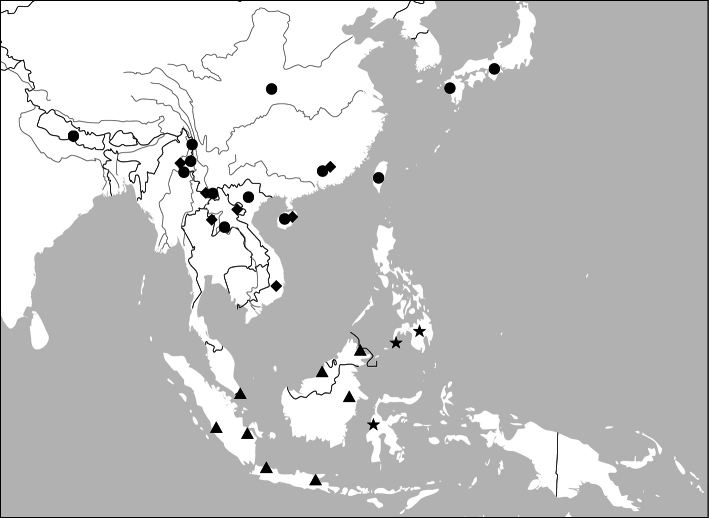
Known distribution of genus *Allocota*: ● *Allocota aurata* (Bates) ★ *Allocota cyanipennis* Heller ▲ *Allocota viridipennis* Motschulsky ◆ *Allocota bicolor* sp. n.

(Map 6). East Asia and Southeast Asia: Japan, China, Indo-China Peninsula, Malay Peninsula, Philippine Islands, Borneo, Sumatra, Java, Sulawesi. Not discovered in South Asia or the eastern part of the Malay Archipelago.

##### Monophyly and relationships.

Monophyly of *Allocota* is suggested by the following apomorphic character states: (1) cleaning spur on protibiae reduced or absent; (2) setigerous pores on 5th interval not restricted to basal half; (3) apical segment of ovipositor only slightly longer than basal width.

##### Taxonomic comments.

We studied the type series and other material of *Taicona aurata* Bates (type-species of *Taicona*), and a male of *Allocota viridipennis* Motschulsky (type-species of *Allocota*) from Java (type locality) that had been compared with the type by Andrewes. Except for color and body size, the only significant difference between them is that setigerous pores are present on the 7th interval in *Allocota*. Even the male genitalia show no obvious differences at the species level (see the detailed discussion under *Allocota aurata*). So it can be inferred that these two species are extremely closely related. As the pore distribution on the 7th interval is not constant even at the species-level (see discussion under *Allocota viridipennis*), we herein synonymize *Taicona* Bates with *Allocota* Motschulsky.

A total of seven species was included in *Allocota* before the present study, but four of them should be transferred to other genera. In the present paper, we combine species previously included in *Taicona* with *Allocota*, propose two new synonyms, and describe a new species. Therefore, in the present concept of *Allocota*, a total of four species is included. Three of these are very closely allied with each other, and strictly allopatric; while the fourth one, *Allocota bicolor* sp. n., is quite different from the others and sympatric with *Allocota aurata*.

**Key to species of *Allocota* Motschulsky**

**Table:** 

1	Protibiae with cleaning spur present, but very fine (Fig. 141); males with terminal labial palpomeres moderately widened, apex truncate; males with adhesive hairs rudimentary on first three mesotarsomeres; 7th interval of elytra with setigerous pores; internal sac of aedeagus without setose area	*Allocota bicolor* sp. n.
–	Protibiae with cleaning spur absent (Fig. 140); males with terminal labial palpomeres slightly widened in middle, not truncate; males with adhesive hairs well developed on first two mesotarsomeres, rudimentary on 3rd mesotarsomeres; 7th interval of elytra usually without setigerous pores (except some specimens from Java and Borneo); internal sac of aedeagus with setose area near middle	2
2	Elytra greenish to cupreous; metasternum and abdomen not distinctly darker than prosternum; species from Japan, Taiwan, and Asian mainland	*Allocota aurata* (Bates)
–	Elytra more or less bluish; metasternum and abdomen much darker than prosternum; species from Malay Archipelago and Malay Peninsula	3
3	Setae on front angles of pronotum very short (Fig. 151); internal sac of aedeagus with setose area divided into two parts, apex of secondary flagellum simple; species from Malaya, Borneo, Sumatra, Java	*Allocota viridipennis* Motschulsky
–	Setae on front angles of pronotum much longer (Fig. 152); internal sac of aedeagus with setose area divided into three parts, apex of secondary flagellum forming a large triangular sclerite; species from the Philippines and Sulawesi	*Allocota cyanipennis* Heller

#### Allocota viridipennis

Motschulsky, 1859

http://species-id.net/wiki/Allocota_viridipennis

Habitus: Figs 14, 15 49 male genitalia: Figs 79 80

Allocota viridipennis Motschulsky 1859: 30 (original: *Allocota*; type locality: Java; syntype deposited in ZMUM); Chaudoir 1877: 205 (*Allocota*, Singapore); Andrewes 1930d: 13 (*Allocota*; catalogue); Andrewes 1933: 12 (*Allocota*, notes on type); Kirschenhofer 1996: 763 (*Allocota*).
Allocota caerulea Andrewes 1933: 12 (type locality: Singapore; lectotype deposited in MNHN); Jedlička 1963: 306 (*Allocota*, misspelled as coerulea); Kirschenhofer 1996: 763 (*Allocota*). **Syn. n.** [Synonym]

##### Type examined.

**Lectotype** of *Allocota caerulea* Andrewes, designated herein (MNHN): male, body length = 6.8 mm, pin mounted, “Ex Musaeo, Chaudoir” [red printed]; “Museum Paris, 1952, Coll. R. Oberthür”; *“viridipennis* / *Motsch. / Singapore / Wallace*” [large box label but pinned under specimen]; “LECTOTYPE ♂/ Allocota caerulea / Andrewes, 1933 / des. SHI H. L. 2011” [red label][Fig. 49]. **Paralectotype** of *Allocota caerulea* Andrewes: a male (MNHN), “♂”; “Ex Musaeo, Chaudoir” [red printed]; “Museum Paris, 1952, Coll. R. Oberthür”; “PARALECTOTYPE ♂/ Allocota caerulea / Andrewes, 1933 / des. SHI H. L. 2011” [red label].

##### Notes on types.

***Allocota viridipennis* Motschulsky**. The type series of *Allocota viridipennis* Motschulsky which was mentioned by Andrewes (1933) should be in Moscow University together with Motschulsky’s main collection. Unfortunately, we have not had opportunity to examine this collection. But in the collection of NHML we found a male of *Allocota viridipennis* from the type locality (Java) that had been compared with the type by Andrewes (it was mentioned by Andrewes 1933, as a female). This specimen is enough for us to recognize this species.

***Allocota caerulea* Andrewes**. Andrewes (1933) indicated that the name *Allocota caerulea* Andrewes was for the species mentioned by Chaudoir (1877) as *Allocota viridipennis*, and type material was in the collection of Oberthür. So, the two specimens from Chaudoir’s ex-collection in MNHN determined by Chaudoir as *Allocota viridipennis* should be the type series of *Allocota caerulea* Andrewes. We designate the male with Chaudoir’s box label pinned under as lectotype (Fig. 49) herein for *Allocota caerulea* Andrewes, for the taxonomic purpose of fixing the name to a single specimen and preventing further uncertainty.

##### Non-type material examined

(Total 33 specimens). **Singapore**: 1 specimen (MNHN), “Singapore, A. Raffray.”. 1 specimen (MNHN), “Singapore”; “Ex Musaeo, H. W. Bates, 1892”. 2 specimens (MNHN), “Singapore”; “ Ex Musaeo, L. Fairmaire, 1896”[Figs 15, 80]. **Sumatra**: 4 specimens (MNHN), “Sumatra, Palembang”. 1 male (NHMB), “W Sumatra prov.; Kerinci Seblat N.P.; 24km NE Tapan; Muara Sako – E env.; 2°05'S, 101°15'E; 400–550m; Dembický leg.; 4–18.iii.2003” **Borneo**: 1 male (MNHN), “Sarawak, Hewilt”. 1 specimen (NHML), “Quop, W. Sarawak, G. E. Bryant., 1.III.14”. 2 specimens (NHML) “N. Borneo, Bettotan, Nr. Sandakan., July 29.1927”. 1 male (NHML) “Sandakan, Borneo, Baker”; “Ex Mus. Coll. Agric. Phil. Is.”; “Allocota viridipennis Motch., H. E. Andrewes det.”. 3 specimens (MNHN), “Est Borneo, Batanbessi, Mem. W. Walsh, 1937”. 1 specimen (MNHN), “Est Borneo, Batan bessi, Me. M. E. Walsh, 1937”. 7 specimens (MNHN), “Est Borneo, Kariovang.”. 1 specimen (NHML), “Borneo”. **Java**: 1 specimen (MNHN), “Java (Meuwen Bay), Détr. De la Sonde, Raffray & Maindron, 1878”. 5 specimens (MNHN), “Museum Paris, Java, Deyrolle 1877”. 1 male (NHML) “23.III.1924, Depok, Karny”; “Ex. Mus. Buitenzorg”; “Allocota viridipennis Motch., Compared with type H. E. A.”[Figs 14, 79].

##### Diagnosis.

Elytra metallic blue, more or less greenish in some specimens; metasternum and abdomen much darker than prosternum; front angles of pronotum with setae very short and fine (Fig. 151); elytral 7th interval with setigerous pores in some specimens; internal sac of aedeagus with setose area divided into two parts, apex of secondary flagellum simple.

In general appearance, this species resembles *Allocota cyanipennis* Heller, but can be distinguished in having: (1) internal sac of aedeagus with setose area divided into two parts, apex of secondary flagellum simple (Figs 79, 80); but in *Allocota cyanipennis*,setose area divided into three parts, apex of secondary flagellum strongly expanded forming a large triangular sclerite (Fig. 81). (2) front angles of pronotum with setae very short and fine; but in *Allocota cyanipennis*, those setae much longer. (3) Setae on elytral lateral margins more distinct in *Allocota cyanipennis*.

##### Description.

Body length 6.0–6.9 mm; head and pronotum orange red to reddish brown; mouthparts earth yellow, palpomeres brownish, apex of terminal palpomeres yellow; 1st antennomere reddish yellow, the remaining antennomeres usually slightly darker; elytra metallic blue, sometimes more or less greenish, or green on lateral areas, elytral suture and lateral margins metallic; legs usually much darker than pronotum, darkest on apical half of femora; ventral side of head and prosternum the same color as dorsal side; metasternum and abdomen brownish to piceous, much darker than prosternum. **Head** glabrous, without punctures, microsculpture indistinct; males with terminal labial palpomeres fusiform, slightly expanded in middle. **Pronotum** glabrous,cordiform, widest at apical one-third; ratio PW/PL 1.22 to 1.32; pronotal base briefly but distinctly lobed; disc moderately convex, microsculpture indistinct, without punctures; front angles with a few fine and short setae (distinctly shorter than in *Allocota cyanipennis*); lateral margins rounded, slightly expanded in middle, strongly sinuate before hind angles; lateral explanate areas moderately wide, without punctures; hind angles more or less distinct, rectangular or nearly so, usually slightly pointed; basal foveae very shallow, without punctures; median line very fine, usually indistinct. **Elytra** with striae slightly sulcate, finely punctate; intervals nearly flat, without accessory setae, with a row of very fine punctures; microsculpture very shallow, isodiametric, or absent; 3rd and 5th intervals with four to ten setigerous pores, their position variable; 7th interval with some setigerous pores or without pore; setae on lateral margins very fine and short, hardly visible. **Legs.** Protibiae with cleaning spur absent (Fig. 140); males with adhesive hairs well developed (two whole rows) on first two mesotarsomeres, rudimentary (two rows, weakly present near apex) on 3rd mesotarsomere. **Male genitalia** with median lobe of aedeagus stout, slightly bent to right side near apex in dorsal view, right margin slightly curved before apical lamella; apical lamella placed on right side, narrow, slightly elongate; internal sac with setose area divided into two parts; apex of secondary flagellum simple, not expanded; trumpet-form expansion with ventral margin more or less expanded (Figs 79, 80). **Female genitalia.** Apical segment of ovipositor very short, slightly longer than width; outer margin straight; inner margin curved, with fine setae on apical half; membranous extension short and wide. Internal reproductive system not studied.

##### Distribution

(Map 6). Singapore, Borneo, Sumatra, Java.

##### Notes on synonym.

We examined some material from Singapore, Borneo and Sumatra identical with the lectotype of *Allocota caerulea*, and a male from Java determined as *Allocota viridipennis* and compared with type by Andrewes. Except color variation on elytra, the only other significant difference is: “*Allocota viridipennis*” from Java with five or six setigerous pores on elytral 7th interval (Fig. 14), but the lectotype of “*Allocota caerulea*” without any pores on 7th interval (Fig. 15). Setigerous pores on 7th interval are not only variable between specimens, e. g., individuals with or without pores can be found from same locality (for example in Java or Borneo), but also in same individual, e. g., one or two pores may present on 7th interval on one elytron, and no such pores on the other elytron. In addition, the aedeagi of these specimens show no significant differences (Figs 79, 80). We therefore synonymize *Allocota caerulea* Andrewes with *Allocota viridipennis* Motschulsky herein.

##### Geographical variation.

As mentioned above, this species is variable in color and setigerous pores on elytra. Usually, the elytra are nearly metallic blue (Figs 15, 49), and without setigerous pores on 7th interval. But in some specimens from Java elytra are distinctly greenish (Fig. 14); in some specimens from Java and Borneo, 7th interval with several setigerous pores.

#### Allocota cyanipennis

Heller, 1923

http://species-id.net/wiki/Allocota_cyanipennis

Habitus: Figs 16 50 male genitalia: Fig. 81

Allocota cyanipennis Heller 1923: 305 (original: *Allocota*; type locality: Mindanao; holotype deposited in SNSD); Andrewes 1930d: 13 (*Allocota*; catalogue); Jedlička 1963: 306 (*Allocota*); Kirschenhofer 1996: 763 (*Allocota*).

##### Type examined.

**Holotype** of *Allocota cyanipennis* Heller, monotypy (SNSD): male, body length = 6.3 mm, board mounted, “Tangcolan / Bukidnon /Baker”; “*cyanipennis /* Typus” [red label]; “*14261*”; “1921 / *3*” [yellow label]; “Staatl. Museum für / Tierkunde, Dresden”[Fig. 50].

##### Notes on types.

Heller (1923) didn’t clearly state this species was based on a single specimen, but he only cited one specimen with a serial number “14261”. So, the holotype (Fig. 50) was originally fixed by monotypy, as the description implies a single specimen.

##### Non-type material examined

(Total 5 specimens). **The** **Philippines**: 1 specimen (NHML), “Philippine Is., Coll. Bottcher., B. M. 1929–201”. 1 male (NHML), “Island of Basilan, Baker”; “Ex Mus. Coll. Agric. Phil. Is.”; “Allocota cyanipennis Heller, Compared with type H. E. A.”[Figs 16, 81]. 1 specimen (NHML), “Tangcolan, Bukienon, Baker”. **Sulawesi**: 1 male and 1 female (NHML), “Celebes”.

##### Diagnosis.

Elytra metallic blue; metasternum and abdomen much darker than prosternum; front angles of pronotum with setae relative long (Fig. 152); elytral 7th interval without setigerous pores; internal sac of aedeagus with setose area divided into three parts, apex of secondary flagellum forming a large triangular sclerite.

This species is closest to *Allocota viridipennis* Motschulsky; differences are presented in the diagnosis part under the latter species.

##### Description.

Body length 6.3–7.1 mm; head and pronotum orange red or brown; mouthparts earth yellow, palpomeres brownish, apex of terminal palpomeres yellow; 1st antennomere reddish, the remaining antennomeres usually slightly darker; elytra metallic blue, elytral suture and lateral margins metallic; legs usually much darker than pronotum, darkest on apical half of femora, basal half usually the same color as pronotum; ventral side of head and prosternum the same color as dorsal side; metasternum and abdomen brownish to piceous, much darker than prosternum. **Head** glabrous, without punctures, microsculpture indistinct; males with terminal labial palpomeres fusiform, slightly expanded in middle. **Pronotum** glabrous,cordiform, widest at anterior third; ratio PW/PL 1.28 to 1.32; pronotal base briefly but distinctly lobed; disc moderately convex, microsculpture indistinct, without punctures; front angles with setae relative long (Fig. 152); lateral margins rounded, slightly expanded in middle, strongly sinuate before hind angles; lateral explanate areas moderately wide, without punctures; hind angles slightly acute, less distinct; basal foveae very shallow, without punctures; median line very fine, usually indistinct. **Elytra** with striae very shallow, finely punctate; intervals nearly flat, without accessory setae, with a row of very fine punctures; microsculpture absent; 3rd and 5th intervals with four to ten setigerous pores, their positions variable; 7th interval without setigerous pores; setae on lateral margins fine and short, but more distinct than in *Allocota viridipennis*. **Legs.** Protibiae with cleaning spur absent (Fig. 140); males with adhesive hairs well developed (two whole rows) on first two mesotarsomeres, rudimentary (two rows, weakly present near apex) on 3rd mesotarsomere. **Male genitalia** with median lobe of aedeagus stout, more distinctly bent to right side near apex than in *Allocota viridipennis* in dorsal view, right margin slightly curved before apical lamella; apical lamella placed on right side, narrow, slightly elongate; internal sac with setose area divided into three parts; apex of secondary flagellum expanded, formed a large triangular sclerite; trumpet-form expansion with ventral margin more or less expanded. Female genitalia not studied (Fig. 81).

##### Distribution

(Map 6). The Philippines, Sulawesi.

##### Geographical variation.

Pronotum and head of two specimens from Sulawesi we examined are brown, much darker than those from the Philippines which are vivid orange.

##### Remarks.

We regard specimens from the Philippines and Sulawesi as a distinct species from *Allocota viridipennis* Motschulsky for the constant difference in internal sac of aedeagus as mentioned above in the diagnosis section under *Allocota viridipennis*. In external characters these two species are very similar, with only slight differences in setae on pronotal front angles (Figs 151, 152) and elytral lateral margins.

#### Allocota aurata

(Bates, 1873) comb. n.

http://species-id.net/wiki/Allocota_aurata

**Figures 19–24.**
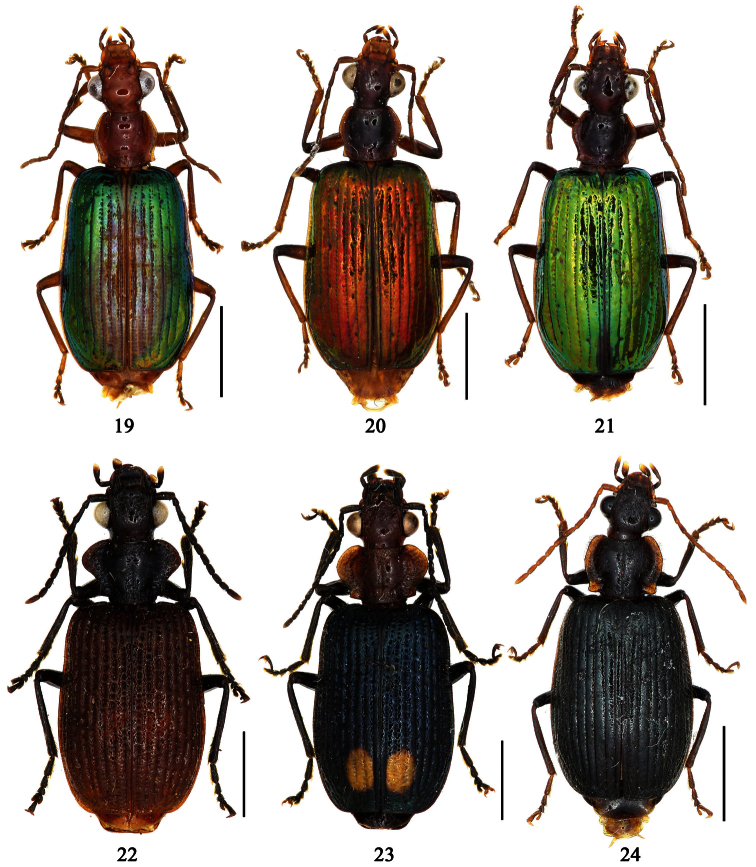
Habitus, scale bars = 2.0 mm: **19** *Allocota aurata* (Bates), a specimen from Hainan **20** *Allocota aurata* (Bates), a specimen from Yunnan **21** *Allocota aurata* (Bates), a specimen from Yunnan **22** *Lachnoderma asperum* Bates, a specimen from Taiwan **23** *Lachnoderma biguttatum* Bates, a specimen from Yunnan **24** *Dasiosoma quadraticolle* sp. n., a paratype from Yunnan.

Habitus: Figs 18 –21 51 male genitalia: Figs 82 83 103, 104 female genitalia: Figs 118 133

Allocota aurata Bates 1873: 315 (original: *Taicona*; type locality: Nagasaki (Japan); lectotype deposited in MNHN); Bates 1876: 5, pl. 1, Fig. 6 (*Taicona*); Jedlička 1963: 448 (*Taicona*); Habu 1967: 141 (*Taicona*); Habu 1982: 106 (*Taicona*); Uéno et al. 1985: 170, pl. 31 Fig. 14 (*Taicona*).
Taicona perroti Jedlička 1963: 448 (type locality: Tonkin; holotype probably deposited in MNHN). **Syn. n.** [Synonym]

##### Type examined.

**Lectotype** of *Taicona aurata* Bates, designated herein (MNHN): male, body length = 7.2 mm, pin mounted, “NAGASAKI”; “TYPE”[red label]; “*Taicona / aurata / Bate*s”; “Ex Musaeo / H. W. Bates / 1892”; “Museum Paris / 1952 / Coll. R. Oberthür”; “LECTOTYPE ♂ / Taicona aurata / Bates, 1873 / Des. SHI H. L., 2011” [red label][Fig. 51]. **Paralectotypes** of *Taicona aurata* Bates: 1 female (MNHN), “NAGASAKI”; “PARATYPE”[red label]; “Ex Musaeo / H. W. Bates / 1892”; “Museum Paris / 1952 / Coll. R. Oberthür”; “*Taicona* / *Bates*”; “PARALECTOTYPE ♀ / Taicona aurata / Bates, 1873 / Des. SHI H. L., 2011” [red label]; Labial removed and pinned isolated with 2 labels: “*Taicona* / *aurata*”; “Ex Musaeo / H. W. Bates / 1892”. 1 female (NHML), “Type” [round label with red circle]; “Japan. / G. Lewis / 1910-320”; “*Taicona / aurata / Bates*”; “PARALECTOTYPE / Taicona aurata / Bates, 1873 / det. SHI H. L. 2011” [red label]. 1 male (NHML), “NAG”; “Japan. / G. Lewis. / 1910-320”; “Ex coll. Brit. Mus.”; “Co-type” [round label with green circle]; “*Taicona* / *aurata*/ *Bates*”; “H. E. Andrewes Coll. / B. M. 1945-97”; “PARALECTOTYPE / Taicona aurata / Bates, 1873 / det. SHI H. L. 2011” [red label].

##### Notes on types.

***Taicona aurata* Bates**. Bates (1873) didn’t state the number of specimens in the type series. We found a total of four specimens bearing Bates’ determination labels in the collection of MNHN and NHML. Two specimens from different collections were both determined and labeled as “type”. We herein designate the male in the collection of MNHN as lectotype (Fig. 51) for taxonomic purpose of fixing the name to unique name-bearing type.

***Taicona perroti* Jedlička**. As indicated in the original description, the holotype of *Taicona perroti* Jedlička should be in MNHN. But we didn’t find it in MNHN or NMPC where the collection of Jedlička is deposited. We suspect that the holotype is probably still in MNHN, but has been misplaced.

##### Non-type material examined

(Total 36 specimens). **Japan**: 2 males, 1 female (OMNH), “KASUGA NARA, 1959.V.31, K. Ueda leg.”[Fig. 82]. 1 female (OMNH), “KASUGA NARA, 1959.V.31, T. Tomiwa leg.”[Fig. 18]. 1 female (NHML), “Japan. G. Lewis. 1910-320.”; “Shiba San Chio 1883”. **Shaanxi**: 1 male, 4 females (IZAS), “Shaanxi, Foping county, Shangshawo, 33.59716°N, 108.01366°E, 1107m, 2007.VIII.15, beating, SHI Hongliang, YANG Ganyan leg.”[Figs 104, 118, 133]. **Guangdong**: 3 males, 1 female (IZAS), “Guangdong, Shixing county, Chebaling, Xianrendong village, 24.73478°N, 114.20727°E, 508m, 2008.VII.23, beating, LIANG Hongbin leg.”. **Hainan**: 1 female (IZAS), “Hainan, Baisha county, Yinggeling Mt., Hongkan reservoir, 19.08121°N, 109.49839°E, 525m, 2009.XI.24, beating, LIANG Hongbin leg.”. 1 male, 2 females (IZAS), “Hainan, Baisha county, Nankai, 19.07926°N, 109.41133°E, 262m, 2009.XI.22, beating, LIANG Hongbin leg.”[Fig. 19]. 1 specimen (IZAS), “Hainan, Baisha county, Nankai, beating, 2009.11.21, LIANG Hongbin leg.”. 1 male, 1 female (IZAS), “Hainan, Baisha county, Nankai, Daoyin village; on vegetation; 19.01021°N, 109.36910°E, 336m, 2010.4.15 D, LIN Meiying leg.”. **Yunnan**: 1 male (IZAS), “China, Yunnan Prov., Fugong, Lumadeng, Yaping vill. Plant beating, 27.13076°N, 98.87447°E; 1295m, 2005.8.25 day, Liang H. B., Zhang J. F. leg.”. 1 male (CAS), “China, Yunnan Prov., Fugong, Pihe Town, Wawa, Plant beating, 26.58548°N, 98.90467°E; 1120m, 2005.8.24 day, Liang H. B., Zhang J. F. leg.”. 2 males, 2 females (IZAS), “China, Yunnan Prov., Tengchong, Qingshui, Rehai, on vegetation; 24.94861°N, 98.45181°E; 1470m, 2006.6.1 day, Liang H. B., Hu P. leg.”[Fig. 20]. 2 males, 1 female (CAS), “China, Yunnan Prov., Tengchong, Mangbang, Longwenqiao, on shrubs; 25.02329°N, 98.67710°E; 1290m, 2006.6.5 day, Liang H. B., Hu P. leg.”. 1 female (IZAS), “China, Yunnan, Ruili, Dengga to Mafengshan. N23.95285, E97.59808 – N23.94485, E97.55647; 927–1207m; 2009.VIII.10, Shi H. L. leg. beating”. 1 female (IZAS), “Yunnan, Xishuangbanna, Xiaomengyang, 850m, 1957.VI.12, WANG Shuyong leg.”. 1 male, 1 female (IZAS), “Yunnan, Jinghong, Nabanhe Reserve, Mandian, N.22.13061, E1000.67377, 718m, 2010.IX.30, LIN Meiying leg.”[Fig. 21]. 1 male, 2 females (IZAS), “Yunnan, Xishuangbanna, Menglun Botany Garden, Lyushilin; 2009.XI.17, 643m, Tang G., Yao Z. Y. leg.”. **Vietnam**: 1 male, 1 female (IZAS), “TONKIN, Hoa-Binh, 1939.VII, leg. A. de Cooman”. 1 male (IZAS), “TONKIN, Hoa-Binh, 1940.VII, leg. A. de Cooman”[Figs 83, 103]. 1 male (IZAS), “TONKIN, Hoa-Binh, leg. A. de Cooman”. 1 specimen (MNHN), “tonkin, Cap. Fouquet”. 1 specimen (MNHN), “Tonkin, Region de, Hoa-Binh”. 1 specimen (MNHN), “Tonkin, Reg. De Hoa-Binh, A. De Cooman, 1929”. **Laos**: 1 male (ZSM), “Laos, Umg. Vientiane, III.-VI.1963”. **Nepal**: 1 female (CRS), “W. Nepal, chitwan Distrikt/ Chitwan Nat. Park, 230m,/ Leg. Probst 28.–30.5.1993”. 1 male (NHMB), “Jiri-Shivalaya (Khimti Khola) 2500–1800m, 11–12.VI.1987”; “C-Nepal, Janakpur, C. J. Rai”

##### Diagnosis.

Elytra metallic green or cupreous–green, disc with more or less distinct reddish patch in some specimens; metasternum and abdomen not darker than prosternum; front angles of pronotum with setae very fine and short (as in Fig. 151); elytral 7th interval without setigerous pores; internal sac of aedeagus with setose area divided into two parts, apex of secondary flagellum simple.

This species is very close to *Allocota viridipennis* Motschulsky. We didn’t find significant difference between their male genitalia. These two species can be distinguished by different color on elytra and ventral side, and also by different distribution (Map 6).

##### Description.

Body length 6.4–8.2 mm; head and pronotum reddish yellow to dark brown; mouthparts yellowish brown, apices of terminal palpomeres paler; 1st antennomere usually slightly paler than the remaining antennomeres; elytra metallic green to cupreous green, sometimes disc with a more or less distinct reddish patch, elytral suture and lateral margins yellowish or metallic; legs reddish yellow to dark brown, usually the same color as pronotum, sometimes slightly darker on apex of femora; ventral side the same color as pronotum; metasternum and abdomen not darker than prosternum. **Head** glabrous, without punctures, microsculpture indistinct; males with terminal labial palpomeres fusiform, slightly expanded in middle. **Pronotum** glabrous,cordiform, widest at apical one-third; ratio PW/PL 1.20 to 1.35; pronotal base briefly but distinctly lobed; disc moderately convex, microsculpture indistinct, slightly transverse, without punctures; front angles with a few very fine short setae (distinctly shorter than in *Allocota cyanipennis*); lateral margins rounded, slightly expanded in middle, strongly sinuate before hind angles; lateral explanate areas moderately wide, without punctures; hind angles usually acute and sharp; basal foveae very shallow, without punctures; median line very fine, usually indistinct. **Elytra** with striae slightly distinct, finely punctate; intervals slightly convex, without accessory setae, with a row of very fine punctures; usually with very faint isodiametric microsculpture; 3rd and 5th intervals with four to ten setigerous pores, their positions variable; 7th interval without setigerous pores; setae on lateral margins very fine and short, hardly visible. **Legs**. Protibiae with cleaning spur absent (Fig. 140); males with adhesive hairs well developed (two whole rows) on first two mesotarsomeres, rudimentary (two rows, weakly present near apex) on 3rd mesotarsomere. **Male genitalia** with median lobe of aedeagus stout, slightly bent to right side near apex in dorsal view, right margin slightly curved before apical lamella; apical lamella placed on right side, narrow, slightly elongate; internal sac with setose area divided into two parts; apex of secondary flagellum simple, not expanded; trumpet-form expansion with ventral margin more or less expanded (Figs 82, 83). **Female genitalia.** Spermatheca straight, tubiform, moderately long; apical part not distinct widened, with very faint ring-sculpture; spermathecal gland inserted near middle of spermatheca, not branched, slightly thickened at base, not longer than spermatheca (Fig. 133). Apical segment of ovipositor very short, slightly longer than width; outer margin straight; inner margin curved, with fine setae on apical half; membranous extension short and wide (Fig. 118).

##### Distribution

(Map 6). Japan, China (Shaanxi, Guangdong, Hainan, Yunnan), Vietnam (Tonkin), Laos, Nepal.

##### Notes on synonym.

Jedlička (1963) described *Taicona perroti* based mainly on the distinct reddish patch on elytral disc and bluish metallic color being different from *Allocota aurata*. These characters are always present in specimens from Tonkin or Hainan. But we didn’t find any difference between this “form” and the typical “*Allocota aurata*” from Japan in male genitalia or external characters except color. The other characters mentioned by Jedlička (1963), such as differences in pronotal shape and punctures on striae, vary among individuals, so we herein synonymize *Taicona perroti* Jedlička with *Taicona aurata* Bates.

##### Geographical variation.

A total of four different color forms was found in this species from different localities: (1) typical “*Allocota aurata*” comes from Japan, and was also found in Shaanxi and Guangdong of China; the head and pronotum are reddish brown; elytra metallic green, disc slightly reddish, not forming distinct patch (Figs 18, 51); (2) specimens from Tonkin, Hainan, Laos and Nepal, namely “*Allocota perroti*”, with head and pronotum vivider compared to specimens from Japan, elytra metallic green, slightly bluish, disc with large reddish patch reaching 5th or 6th interval, the patch much more distinct than in the “typical *Allocota aurata*” (Fig. 19); (3) specimens from the southern part of Yunnan (Xishuangbanna) with head, pronotum and legs dark brown; elytra metallic green, disc not reddish (Fig. 21); (4) specimens from the western part of Yunnan (Fugong, Tengchong, Ruili) with head and pronotum slightly darker than in the “typical form”, elytra distinctly cupreous, disc not reddish (Fig. 20). These forms were only different in color, but other characters including male genitalia are the same. So we consider them as geographical variation rather than distinct species or subspecies.

##### Remarks.

This species has male genitalia very similar with *Allocota viridipennis* Motschulsky implying they could be synonyms. However, their constant differences on elytral and ventral color, as well as the distribution gap make us quite doubtful to synonymize them at present. Therefore, we keep them as distinct species before more specimens are available, especially those from Indo-China.

The shape of right paramere apex is quite different among specimens of this species. (Figs 103, 104) According to examined materials of some other species in Physoderina, we speculate that in this subtribe the shape of right paramere is an intraspecifically variable character, but less variable between different genera (Figs 95–110). Therefore, this character has no taxonomic importance at both species and genus levels.

#### Allocota bicolor

Shi & Liang sp. n.

urn:lsid:zoobank.org:act:E45EC11F-FE7D-438D-8822-50010A352653

http://species-id.net/wiki/Allocota_bicolor

Habitus: Figs 17 52 male genitalia: Figs 84 105 female genitalia: Figs 119 134

##### Type material.

**Holotype** (IZAS): male, body length = 8.3 mm, pin mounted, genitalia dissected and deposited in microvial pinned under specimen, “China, Yunnan, Ruili / Dengga to Mafengshan; / 23.95285°N, 97.59808°E / 23.94485°N, 97.55647°E”; “927–1207m; 2009.VIII.10 / SHI H. L. leg.; Beating / Inst. of Zool., CAS / 瑞丽市等噶至麻风山"; “IOZ(E)1891845”; "HOLOTYPE ♂/ Allocota bicolor/ new species / Des. SHI H. L. 2011” [red label][Figs 52, 84, 105]. **Paratypes** (10 males, 13 females): **Yunnan**: 2 males (IZAS), “China, Yunnan, Ruili / Dengga to Mafengshan; / 23.95285°N, 97.59808°E / 23.94485°N, 97.55647°E”; “927–1207m; 2009.VIII.10 / SHI H. L. leg.; Beating / Inst. of Zool., CAS / 瑞丽市等噶至麻风山"; “PARATYPE/ Allocota bicolor/ new species / Des. SHI H. L. 2011” [red label]. 1 male 2 females (IZAS) [the male preserved in 100% alcohol, females pinned], “CHINA, Yunnan, Ruili, / Dengga to Sepeng bridge; / 23.95285°N, 97.59808°E / 23.97518°N, 97.56944°E”; “927–807m; 2009.VIII.11 / SHI H. L. leg., Beating; / Inst. of Zool., CAS / 瑞丽市等噶至色蓬桥”; “PARATYPE/ Allocota bicolor/ new species / Des. SHI H. L. 2011” [red label] [Fig. 134]. 1 female (HBUM), “2005-VIII-3 / 云南瑞丽市勐秀 / 2150m 毛本勇 / 河北大学博物馆” [2005-VIII-3 / Yunnan, Ruili, Mengxiu / 2150m Mao Benyong leg. / Hebei University Museum]; “PARATYPE/ Allocota bicolor/ new species / Des. SHI H. L. 2011” [red label]. 1 male 1 female (CBW), “CHINA, Yunnan Prov., / Xishuangbanna, Menglun / Reserve, west part. 570m / 2009.VI.2; BI Wenxuan leg.”; “PARATYPE/ Allocota bicolor/ new species / Des. SHI H. L. 2011” [red label]. **Guangdong**: 1 female (IZAS), “CHINA, Guangdong Prov., / Shixing County, Mt. Che- / baling, Xianrendong; / 24.73478°N, 114.20727°E”; “508m; 2008.VII.23 / vegetation beating; / LIANG Hongbin leg. / Ins. of Zool., CAS”; “PARATYPE/ Allocota bicolor/ new species / Des. SHI H. L. 2011” [red label][Fig. 119]. 1 female (IZAS) “CHINA, Guangdong / Shixing, Chebaling / Chayuan (Tea Garden) / N24.72320, E114.25640”; “354m, 2008.7.27 day / Liang H. B.; vegetation; Institute of Zoology / 广东始兴县车八岭”; “PARATYPE/ Allocota bicolor/ new species / Des. SHI H. L. 2011” [red label]. **Hainan**: 1male, 1 female (IZAS), “CHINA, Hainan, / Ledong, Jianfengling, / 5th area; beating / 18.73263°N, 108.87023°E”; “978m; 2009.XII.3; Day / Lin Meiying coll. / Inst. of Zoology, CAS / 尖峰岭核心区五区”; “PARATYPE/ Allocota bicolor/ new species / Des. SHI H. L. 2011” [red label][Fig. 17]. 1 male (IZAS) [preserved in 100% alcohol], “CHINA, Hainan, Wuzhi- / shan Mt., way to the peak; / beating; / 18.90161°N, 109.68844°E”; “997m; 2009.XI.28 / LIANG Hongbin leg. / Inst. of Zool., CAS / 海南五指山主峰”; “PARATYPE/ Allocota bicolor/ new species / Des. SHI H. L. 2011” [red label]. **Vietnam**: 1 female (NNML), “Museum Leiden / Viet Nam (Dak Lak Prov.) Chu / Yang Sin N. P.: 6–8 km S of / head quarters: S of Dam / construction-site. 2–10.vi.2007. / Leg. C. van Achterberg, R. de / Vries & E. Gasso Miracle”; “primary evergreen forest near / stream; in malaise traps; 800- / 820m, 12°26'26.3"N / 108°19'58.5"E”. **Laos**: 2 males, 2 females (NHMB), “LAOS-NE, Houa Phan prov., / 20°11–13'N 103°59'–104°01'E, / Ban Saluei – Phou Pane Mt., / 9.–17.vi.2009, 1300–1900 m, / Michael Geiser leg.”; “NHMB Basel, NMPC Prague / Laos 2009 Expedition: / M. Brancucci, M. Geiser, / Z. Kraus, D. Hauck, V. Kubáň”; “PARATYPE/ Allocota bicolor/ new species / Des. SHI H. L. 2011” [red label]. 2 males, 2 females (NHMB), “LAOS-NE, Houa Phan prov., / ~20°13'N, 104°00'E, / PHOU PANE Mt., / 1.–16.vi.2009, 1350~1500 m, / M. Brancucci leg.”; “NHMB Basel, NMPC Prague / Laos 2009 Expedition: / M. Brancucci, M. Geiser, / Z. Kraus, D. Hauck, V. Kubáň”; “PARATYPE/ Allocota bicolor/ new species / Des. SHI H. L. 2011” [red label]. **Thailand**: 1 female (CMB), “Thailand, Prov. Nan. Bo Khua/ 19.4-7.5.2004/ Moravec Petr”.

##### Diagnosis.

Elytra metallic blue, disc without reddish patch; metasternum and abdomen not darker than prosternum; front angles of pronotum with long setae (Fig. 153); elytral 7th interval with setigerous pores; protibiae with cleaning spur present but very fine (Fig. 141); internal sac of aedeagus without setose area near middle, apex of secondary flagellum simple.

From dorsal color, this new species resembles *Allocota viridipennis* Motschulsky or *Allocota cyanipennis* Heller, but it is actually more isolated in the genus than it appears. The new species can be distinguished from the other three species of the genus in having: (1) protibiae with cleaning spur present, but absent in other species; (2) males with adhesive hairs rudimentary on first three mesotarsomeres, but well developed on 1st and 2nd mesotarsomeres in other species; (3) males with terminal labial palpomeres truncate, but fusiform in the other species; (4) setae on pronotal front angles longer than in other species; (5) internal sac of aedeagus without setose area near middle.

##### Description.

Body length 7.5–8.7 mm; head and pronotum vivid orange red; mouthparts yellowish brown, palpomeres dark brown, apex of terminal labial and maxillary palpomeres yellow; base of 1st antennomere yellowish, remaining part of 1st antennomere and 2nd to 4th antennomeres piceous, gradually turning to brownish after 5th antennomere; elytra metallic blue, elytral suture and lateral margins metallic; legs with most parts much darker than pronotum, coxae, trochanters and base of femora yellow, apical part of femora and tibiae nearly black, tarsomeres usually dark brown; ventral side of head and prosternum almost the same color as dorsal side; metasternum and abdomen yellowish, not darker than prosternum. **Head** glabrous, without punctures, microsculpture indistinct; males with terminal labial palpomeres expanded at apex, truncate but not strongly securiform. **Pronotum** glabrous,cordiform, widest at about apical one-third; ratio PW/PL 1.30 to 1.40; pronotal base briefly but distinctly lobed; disc moderately convex, microsculpture indistinct, without punctures; front angles with long setae, distinctly longer than other species of the genus; hind angles with a few secondary setae; lateral explanate areas moderately wide, without punctures; lateral margins slightly expanded in middle, usually slightly sinuate before hind angles; hind angles usually rounded or subrectangular, not sharp or projected (Fig. 157), but in two females from Guangdong, hind angles sharp, acute, distinctly projected (Fig. 156); basal foveae very shallow, without punctures; median line very fine, indistinct; disc finely transversely rugose. **Elytra** with striae slightly sulcate, finely punctate; 7th, 8th striae and base of the remaining striae nearly unimpressed; intervals not distinctly convex, without accessory setae; microsculpture absent; 3rd, 5th and 7th intervals with four to ten setigerous pores, their positions variable, most of them on center of interval; lateral margins with some short and fine but distinct setae. **Legs.** Protibiae with cleaning spur present, but fine and short (Fig. 141); males with adhesive hairs rudimentary (two rows, weakly present near apex) on first three mesotarsomeres. **Male genitalia** with median lobe of aedeagus stout, apex slightly bent to right side in dorsal view, right margin not curved before apical lamella; apical lamella placed on right side, triangular, length equal to its basal width, apex not sharp; internal sac without setose area near middle; secondary flagellum reaching apical orifice, apex simple; trumpet-form expansion with ventral margin not expanded (Fig. 84). **Female genitalia.** Spermatheca straight, tubiform, moderately long; apical half slightly widened, apical third with distinct ring-sculpture; spermathecal gland inserted near middle of spermatheca, not branched, fine, base not distinctly thickened, much longer than spermatheca (Fig. 134). Apical segment of ovipositor very short, slightly longer than width; outer margin straight; inner margin curved, with fine setae on apical half; membranous extension fine, placed on the outer side of apex (Fig. 119).

##### Distribution

(Map 6). China (Yunnan, Guangdong, Hainan), Vietnam, Laos, Thailand.

##### Etymology.

The name “*bicolor*” is a Latin noun, referring to the sharp contrast between the orange head and pronotum and the metallic blue elytra.

##### Geographical variation.

Two female paratypes coming from Guangdong are slightly different from the holotype from Yunnan in pronotal shape. Their pronotum hind angles are sharper and more projected, and lateral margins are more strongly sinuate before hind angles (Fig. 156) than in the holotype (Fig. 157). But the other characters are the same, and we consider they are just geographical variations.

##### Remarks.

The new species is a special lineage in *Allocota*, and could be the most primitive one of the genus, based on some primary characters such as presence of cleaning spur on protibiae. Moreover, the female reproductive system and male secondary characters (labial palpomeres, adhesive hairs on tarsomeres, setae on terminal sternum) are similar to *Physodera eschscholtzii*, but they are totally different in ovipositor. Similarity between these two species may also suggest the affinity between genera *Allocota* and *Physodera*.

#### Genus Lachnoderma

W. J. Macleay, 1873

http://species-id.net/wiki/Lachnoderma

Habitus: Figs 22, 23 male genitalia: Figs 85 106 female genitalia: Figs 120 135

Lachnoderma Macleay 1873: 321; Chaudoir 1877: 212; Jedlička 1963: 302; Habu 1967: 133; Tian and Deuve 2001: 123.

##### Type-species:

*Lachnoderma cinctum* Macleay, 1873, by monotypy.

##### Diagnosis.

Dorsal side densely and evenly pubescent, pubescence long and erect; labrum and mandibles with long accessory setae (as long as primary ones on labrum); mentum tooth bifid; basal foveae of pronotum wide, not forming groove; elytral striae very coarsely punctate; males with adhesive hairs absent on all tarsomeres; aedeagus with main flagellum of internal sac projected out from apical orifice. This genus is most similar to *Dasiosoma*; their differences are presented in the diagnosis of *Dasiosoma*.

##### Generic characters.

Body length 7–10 mm; dorsal side generally reddish brown to dark brown, elytra blue in some species, elytra unicolored or with two small spots near apex. Dorsal side densely and evenly pubescent, pubescence coarse, long and erect, microsculpture indistinct. **Head** densely pubescent, pubescence rather long, occiput nearly glabrous. Eyes hemispherical, strongly prominent; tempora half length of eyes, gradually narrowed behind eyes; vertex slightly tumid. Antennae extended to about basal one-fourth of elytra. Labrum slightly widened to apex, apical margin usually slightly curved, sometimes with indistinct longitudinal ridge in middle, labrum with faint microsculpture, with long accessory setae, accessory setae as long as six primary setae; mandibles distinctly widened, outer margins rounded, surface with long accessory setae; terminal maxillary palpomeres slightly expanded in both sexes; terminal labial palpomeres strongly expanded in both sexes, more strongly securiform and truncate apically in males, slightly less widened in females; ligula with apex slightly projected, with several long setae; paraglossae membranous, not longer than ligula, adnate; mentum tooth bifid, with several long setae on middle area of mentum, sometimes with a few additional short setae; submentum with two long setae; genae with long setae beneath eyes; gula nearly glabrous except anterior part. **Pronotum** wider than head; disc, lateral explanate areas densely pubescent; lateral margins setose along full length; mid-lateral primary setae present, hardly distinguishable from long secondary setae on lateral margins; basal foveae wide, not forming groove; pronotal base strongly lobed; lateral margins completely rounded in middle, strongly sinuate before hind angles; hind angles sharp, rectangular or acute, projected or not. **Elytra** slightly expanded to apex; apex truncate, sutural angles not projected, outer angles completely rounded; basal margination only reaching 4th interval; basal pores distinct; striae not sulcate, coarsely punctate; intervals flat; all intervals densely and evenly pubescent, pubescence long, erect; in some species, intervals rugose or irregularly punctate, striae hardly distinguishable; primary setigerous pores not distinguishable among long pubescence; 7th and 8th intervals slightly tumid near apex. **Ventral side** with long and dense pubescence, sparser on proepisterna; males with apex of terminal sternum moderately emarginate, straight or slightly curved in females; terminal sternum with two to four setae on each side in both sexes. **Legs** short; protibiae with cleaning spur well developed, distant from inner margin; tarsi widened, 4th tarsomere bifid, claws pectinate; males with adhesive hairs absent on all tarsomeres. **Male genitalia** with median lobe of aedeagus stout, not twisted, slightly bent to right side in dorsal view; apical orifice opened apically; apical lamella short and wide; dorsal surface with some fine setae subapically; internal sac with main flagellum slightly thickened, apex projected out from apical orifice, trumpet-form expansion distinct; apical bursa absent; secondary flagellum distinct; an elongate sclerite present near base, subparallel to the trumpet-form expansion (Fig. 85). **Female genitalia**. Spermatheca tubular, apical part with ring-sculpture, inserted at base of common oviduct; spermathecal gland much longer than spermatheca, inserted near middle of spermatheca; spermatheca not distinctly bent (Fig. 135). Apical segment of ovipositor coniform, slightly curved to outer side; apex sharp; apex with membranous extension long and sharp (Fig. 120).

##### Distribution.

Oriental and Australian Region.

##### Monophyly and relationships.

*Dasiosoma* is supposed to be closest to *Lachnoderma* by: (1) surface strongly setose; (2) males with adhesive hairs absent on all tarsomeres.

Monophyly of *Lachnoderma* is suggested by the following apomorphic character states: (1) labrum and mandibles with long accessory setae; (2) mentum tooth bifid; (3) elytral striae strongly punctate; (4) median lobe of aedeagus with main flagellum pointed out from apical orifice, apical bursa absent.

##### Taxonomic comments.

Jedlička (1963) revised the Oriental species of this genus, totalling four species. Later, Louwerens (1952, 1967) and Kirschenhofer (1996) added four Oriental species. Tian and Deuve (2001) added six new species, and provided a key to the Chinese and Vietnamese species.

So far, 15 available names belonging to *Lachnoderma* have been published, excluding those removed to *Dasiosoma* in the present paper. Some species haven’t been well defined, while others were merely defined using characters which seem intraspecifically variable, such as body color and elytral punctures. So a revision of this genus seems necessary.

We don’t intend to revise this genus in the present paper, because specimens are so scarce in the collections examined. Moreover, we haven’t found sufficient reliable characters between the known species, even in the male genitalia, since the median lobe of the aedeagus shows only trivial specific differences in the genus.

**List of species:**

***Lachnoderma asperum*** Bates, 1883: 285. Type locality: Miyanoshita (Japan). Holotype deposited in NHML.

***Lachnoderma biguttatum*** Bates, 1892: 424. Type locality: Shwegoo (Burma). Holotype deposited in MSNG.

***Lachnoderma rufithorax*** Kirschenhofer, 1996: 760 (as subspecies of *Lachnoderma biguttatum* Bates). Type locality: Kathmandu (Nepal). Syntypes deposited in NMPC and ZSM.

***Lachnoderma chebaling*** Tian & Deuve, 2001: 131. Type locality: Chebaling (Guangdong, China). Holotype deposited in SCAU.

***Lachnoderma cheni*** Tian & Deuve, 2001: 130. Type locality: Huaping (Guangxi, China). Holotype deposited in SCAU.

***Lachnoderma cinctum*** W. J. Macleay, 1873: 321. Type locality: Clarence River (Australia). Syntype deposited in MAMU.

***Lachnoderma confusum*** Tian & Deuve, 2001: 129. Type locality: Kouy-tchéou (Guizhou, China). Holotype deposited in MNHN.

***Lachnoderma foveolatum*** Sloane, 1915: 472. Type locality: Cairns (Australia). Holotype deposited in ANIC.

***Lachnoderma metallicum*** Tian & Deuve, 2001: 129. Type locality: Jinghong (Yunnan, China). Holotype deposited in SCAU.

***Lachnoderma nideki*** Louwerens, 1952: 218. Type locality: Depok (Java). Holotype deposited in NNML.

***Lachnoderma philippinense*** Jedlička, 1934: 120. Type locality: Philippines. Holotype deposited in NMPC.

***Lachnoderma polybothris*** Louwerens, 1967: 207. Type locality: Balabac (Philippines). Holotype deposited in ZMUC.

***Lachnoderma tricolor*** Andrewes, 1926: 289. Type locality: Singapore. Holotype deposited in NHML.

***Lachnoderma vietnamense*** Kirschenhofer, 1996: 759. Type locality: Sapa (Vietnam). Holotype deposited in NHMW.

***Lachnoderma yingdeicum*** Tian & Deuve, 2001: 132. Type locality: Shimentai (Guangdong, China). Holotype deposited in SCAU.

#### Genus Dasiosoma

Britton, 1937

http://species-id.net/wiki/Dasiosoma

Dasiosoma Britton 1937: 233; Basilewsky 1949: 221 (key to species); Basilewsky 1968: 145.
Teradaia Habu 1979a: 65. Type-species: *Teradaia bella* Habu, 1979a, by original designation. **Syn. n.** [Synonym]

##### Type-species:

*Dasiosoma testaceum* Britton, 1937, by original designation.

##### Diagnosis.

Dorsal side evenly pubescent, elytra with accessory setae on all intervals; labrum and outer scrobe of mandibles nearly glabrous; pronotum with basal foveae deep and narrow, forming a pair of deep grooves; elytral striae distinct, finely punctate; aedeagal internal sac with main flagellum developed, apex reaching the apical orifice, not projecting out from it.

Some species of the genus were placed in *Lachnoderma* by mistake (Bates 1883; Tian and Deuve 2001). These two genera may be closely allied, but *Dasiosoma* can be distinguished in having: (1) labrum and outer scrobe of mandibles nearly glabrous, at most labrum with a few very short fine accessory setae, but in *Lachnoderma*, labrum and mandibles always with long accessory setae, accessory setae on labrum as long as the six primary setae near apex; (2) mentum tooth simple, but bifid in *Lachnoderma*; (3) pronotum basal foveae much deeper and groove-like, but shallower and wide in *Lachnoderma*; (4) punctures in striae much finer than in *Lachnoderma*; (5) median lobe of aedeagus slender, apical bursa present, main flagellum not projected out from apical orifice, but *Lachnoderma* with median lobe of aedeagus stout, apical bursa absent, main flagellum projected out from apical orifice.

##### Generic characters.

Dorsal side yellow, brown or piceous, elytra unicolored or bicolored, usually with weak metallic luster. **Head** densely pubescent, occiput and central area of clypeus nearly glabrous; eyes hemispherical, strongly prominent; tempora shorter or slightly longer than half length of eyes, abruptly or gradually narrowed behind eyes; vertex slightly or strongly tumid. Antennae extended to basal one-fifth of elytra; 1st antennomere slightly curved, 3rd slightly longer than 4th. Labrum slightly widened to apex, anterior margin straight or slightly curved, with faint microsculpture, glabrous or with a few very fine and short secondary setae; mandibles distinctly widened, outer margins rounded, glabrous on outer scrobe, with some setae arranged along dorsal ridge; terminal maxillary palpomeres fusiform in both sexes; terminal labial palpomeres strongly securiform, truncate apically in males, less widened in females; ligula with apex slightly projected, with four long setae and some short setae; paraglossae membranous, not longer than ligula, narrow, adnate; mentum tooth simple, short and rounded, with two long setae near base, sometimes with a few additional short setae; submentum with two long setae; genae with long setae beneath eyes; gula glabrous except apex. **Pronotum** wider than head; disc, lateral explanate areas and lateral margins densely and evenly pubescent; mid-lateral primary setae present, distinctly longer than accessory ones; basal foveae deep, forming a pair of deep grooves, extended anteriorly or anteriomedially; median line deep, reaching apical and basal margins; pronotal base more or less lobed; lateral margins rounded or narrowed in middle, more or less sinuate before hind angles; hind angles sharp, rectangular or acute, projected or not. **Elytra** slightly narrow, lateral margins parallel or expanded to apex; apex truncate, sutural angles not projected, outer angles completely rounded; basal margination reaching 3rd interval; basal pores large; striae shallow, distinct, finely punctate; intervals slightly convex; all intervals evenly pubescent, pubescence fine and long, erect; primary setigerous pores small, indistinct, with setae longer than erect pubescence, three or four pores on 3rd interval, one or two pores on base of 5th; 7th and 8th intervals slightly tumid near apex. **Ventral side** with long dense pubescence except posterior area of proepisterna, sparser on mesosternum and central area of metasternum; males with apex of terminal sternum moderately emarginate, with two pairs of setae, females straight or slightly emarginate, with two pairs of setae. **Legs** short; protibiae with cleaning spur well developed, distant from inner margin; tarsi widened, 4th tarsomere bifid, claws pectinate; males with adhesive hairs absent on all tarsomeres. **Male genitalia** with median lobe of aedeagus not twisted, strongly expanded and bent at base; strongly bent to right side in dorsal view; apical orifice opened apically; dorsal surface with a few fine setae subapically; internal sac with main flagellum slender, apex nearly reaching apical orifice, not projected out from it, trumpet-form expansion small; apical bursa present; secondary flagellum distinct. **Female genitalia**. Spermatheca tubular, with ring-sculpture, inserted on bursa copulatrix; spermathecal gland very short or longer than spermatheca, inserted near middle of spermatheca; spermatheca not bent. Apical segment of ovipositor scimitar-shaped, curved to outer side; apex sharp; apex with membranous extension short but sharp.

##### Distribution.

South China, Indo-China Peninsula, South Asia, African mainland. Not discovered in the Philippines, Malay Archipelago or Madagascar.

##### Monophyly and relationships.

Relationship between *Dasiosoma* and *Lachnoderma* is discussed under *Lachnoderma*. Monophyly of *Dasiosoma* is suggested by the following apomorphic character states: (1) basal foveae of pronotum deep and narrow; (2) median lobe of aedeagus strongly expanded and bent at base.

##### Taxonomic comments.

Habu (1979a) described the genus *Teradaia* for his new species *Teradaia bella*, and indicated that it is allied with *Lachnoderma*. But the Palaearctic catalogue (Löbl and Smetana 2003) included *Teradaia* in Pericalina without explanation. We examined types of all four species from Africa belonging to *Dasiosoma* in the former concept, and dissected a paratype of *Dasiosoma ivorense*. The male genitalia (Fig. 86) are not significantly different from those of *Teradaia bella* and its allied species (Figs 87–90), and the small differences of external morphological characters between species from the two continents (see the key, first item) are not enough to be considered of genericlevel difference. So we herein synonymize *Teradaia* with *Dasiosoma*, and include *Dasiosoma* in Physoderina, not Pericalina.

Moreover, two Asian species (*Singilis hirsutus* Bates and *Lachnoderma maindroni* Tian & Deuve) that used to be placed in *Lachnoderma*, and also *Diamella indica* Kirschenhofer, are closer to *Teradaia bella* Habu than the other species of *Lachnoderma* or *Diamella*. So we combine these three species with *Dasiosoma* and describe a new species from Asia in the present paper. Thus the current concept of *Dasiosoma* includes nine species, with four of them from Africa, and the other five from Asia. The definitive characters of *Dasiosoma* are in the diagnosis.

The four Africa species (*Dasiosoma testaceum* Britton, 1937, *Dasiosoma basilewskyi* Shi & Liang nom. n., *Dasiosoma sudanicum* Basilewsky, 1949 and *Dasiosoma ivorense* Basilewsky, 1968) are very similar to each other. Two of them were described based each on a single female. Basilewsky (1949, 1968) provided a key and diagnosis to these four species, but when we examined types of all four of these species, it is still difficult for us to clearly distinguish them all. So we leave this part of review work for future study when more material becomes available. In the present paper, we just revise the Asian fauna of *Dasiosoma*.

##### Key to species of *Dasiosoma* Britton

**Table:** 

1	Hind angles of pronotum acute, strongly projected outward; pronotal base strongly lobed; elytra with 3rd to 5th intervals distinctly depressed subapically	African species (*Dasiosoma testaceum*, *Dasiosoma basilewskyi*, *Dasiosoma sudanicum*, *Dasiosoma ivorense*)
–	Hind angles nearly rectangular, not strongly projected outward; pronotal base briefly lobed; elytral disc not depressed (Oriental species)	2
2	Basal foveae of pronotum straight, subparallel with median line, disc with distinct elongate depression on each side; body slender, ratio EL/EW more than 1.55; elytra dark blue, usually with a large reddish yellow patch occupying inner 3–4 intervals	*Dasiosoma bellum* (Habu)
–	Basal foveae of pronotum curved anteromedially, disc without or with barely visible depression; body stouter, ratio EL/EW less than 1.50; elytra reddish brown to dark brown, without distinct large patch on the center	3
3	Vertex strongly tumid; pronotum wider, lateral margins strongly expanded and completely rounded in middle; elytral color much darker than head and pronotum (Figs 57, 58)	4
–	Vertex slightly tumid; pronotum narrower, lateral margins slightly expanded and less rounded in middle; elytral color close to (only slightly darker than) head and pronotum (Figs 59, 61)	5
4	Elytra dark brown, each side with a yellowish spot subapically, spots nearly joining at elytral suture; tempora gradually narrowed behind eyes; elytra wider, ratio EL/EW 1.30; body length 6.4 mm; species from India (Fig. 57)	*Dasiosoma indicum* (Kirschenhofer)
–	Elytra uniformly dark brown, without spots; tempora abruptly narrowed behind eyes; elytra narrower, ratio EL/EW more than 1.35; body longer than 7.0 mm; species from Tonkin (Fig. 58)	*Dasiosoma maindroni* (Tian & Deuve)
5	Pronotum quadrate, lateral margins nearly straight, slightly sinuate before hind angles; elytral striae shallower, with finer punctures, intervals slightly convex; head and pronotum brown to dark brown, elytra piceous, sometimes disc with a large indistinct brownish patch; Yunnan, Laos	*Dasiosoma quadraticolle* sp. n.
–	Pronotum weakly cordiform, lateral margins slightly arcuate, strongly sinuate before hind angles; elytral striae deeper, with coarser punctures, intervals distinctly convex; dorsal side uniformly reddish brown; Hong Kong	*Dasiosoma hirsutum* (Bates)

#### Dasiosoma testaceum

Britton, 1937

http://species-id.net/wiki/Dasiosoma_testaceum

Habitus: Fig. 53

Dasiosoma testaceum Britton 1937: 234 (original: *Dasiosoma*; type locality: N. W. Rhodesia; syntypes deposited in NHML); Basilewsky 1949: 222 (*Dasiosoma*).

##### Type examined.

**Syntypes** of *Dasiosoma testaceum* Britton: 1 male (NHML), “*Holo-*Type” [round label with red circle]; “N. W. Rhodesia: / Lukanga. / l.v.1915. / H. C. Dollman”; “H. C. Dollman / Coll. 1919-79”; “*Dasiosoma / testacea g. sp. n. / Holotype /* E. B. Britton. / det. *28.vii*.193*7*”[Fig. 53]. 25 ex. (NHML), the same collection data but labeled as paratype.

##### Notes on types.

The original description mentioned a total of 30 specimens of *Dasiosoma testaceum*, but no holotype was originally fixed. So, all specimens we found in NHML are syntypes, although the first one was labeled as “holotype” by Britton himself.

#### Dasiosoma basilewskyi

Shi & Liang nom. n.

Habitus: Fig. 54

Dasiosoma hirsutum Basilewsky 1949: 222 (type locality: Congo; holotype deposited in MRAC). Junior secondary homonym of *Dasiosoma hirsutum* (Bates, 1873) [Synonym]

##### Type examined.

**Holotype** of *Dasiosoma hirsutum* Basilewsky, monotypy (MRAC): female, body length = 6.9 mm, board mounted, “Holotype / *hirsutum / Basil*.” [red label]; “Musee Du Congo / Ituri: / La Moto: Madyu / L. Burgeon”; “*Dasiosoma / sp. n. /* E. B. Britton. / det. *5.v*.193*8*”; “*Dasiosoma / hirsutum, sp. n*. / P. Basilewsky det., 19*48*”[Fig. 54].

##### Etymology.

We propose this name in honor of P. Basilewsky, a well known specialist on African Carabidae, who first described this species.

##### Remarks.

In moving *Singilis hirsutus* Bates to *Dasiosoma*, the name *Dasiosoma hirsutum* Basilewsky becomes a junior secondary homonym. So we give a new name for this species herein.

#### Dasiosoma sudanicum

Basilewsky, 1949

http://species-id.net/wiki/Dasiosoma_sudanicum

Habitus: Fig. 55

Dasiosoma sudanicum Basilewsky 1949: 222 (original: *Dasiosoma*; type locality: Congo; holotype deposited in MRAC).

##### Type examined.

**Holotype** of *Dasiosoma sudanicum* Basilewsky, by monotypy (MRAC): female, body length = 5.3 mm, micropin mounted, “Holotype / *sudanicum / Basil*.” [red label]; “Musee Du Congo / Soudan: *mongalla a*/ *Shanile -1-vi-1977* / L. Burgeon”; “R. Det. / *JJ / 3418*”; “*Dasiosoma / sp. n. /* E. B. Britton. / det. *5.v*.193*8*”; “HOLO / TYPE”[red label]; “R. DET. / *H. H*. / 5341”; “*Dasiosoma / sudanicum, sp. n*. / P. Basilewsky det., 19*48*”[Fig. 55].

##### Non-type material examined.

1 male (CCA): “Congo Belge, P.N.G. Miss. H. De Saeger II/fd/15, 9.X.1951, Réc. H. De Saeger. 2578”, “Dasiosoma sudanicum Basilew. P. Basilewsky det., 1959”.

#### Dasiosoma ivorense

Basilewsky, 1968

http://species-id.net/wiki/Dasiosoma_ivorense

Habitus: Fig. 56 male genitalia: Figs 86 107

Dasiosoma ivorense Basilewsky 1968: 145 (original: *Dasiosoma*; type locality: Lamto; holotype deposited in MNHN).

##### Type examined.

**Holotype** of *Dasiosoma ivorense* Basilewsky, by original designation (MNHN): male, body length = 5.9 mm, board mounted, “LAMTO (Toumodi) / Côte d’Ivoire / *B47* / *10 XII 63*”; “GENIT. ♂ / *67.314.1*”; “*99*”; “TYPE” [red label]; “*Dasiosoma / ivorense. sp. n*. / P. Basilewsky det., 19*67*”[Fig. 56]. **Paratypes** of *ivorense* Basilewsky: 51 ex. (MNHN), the same locality but different collecting dates and labeled as paratypes [Figs 86, 107].

#### Dasiosoma bellum

(Habu, 1979a) comb. n.

http://species-id.net/wiki/Dasiosoma_bellum

**Figures 25–30.**
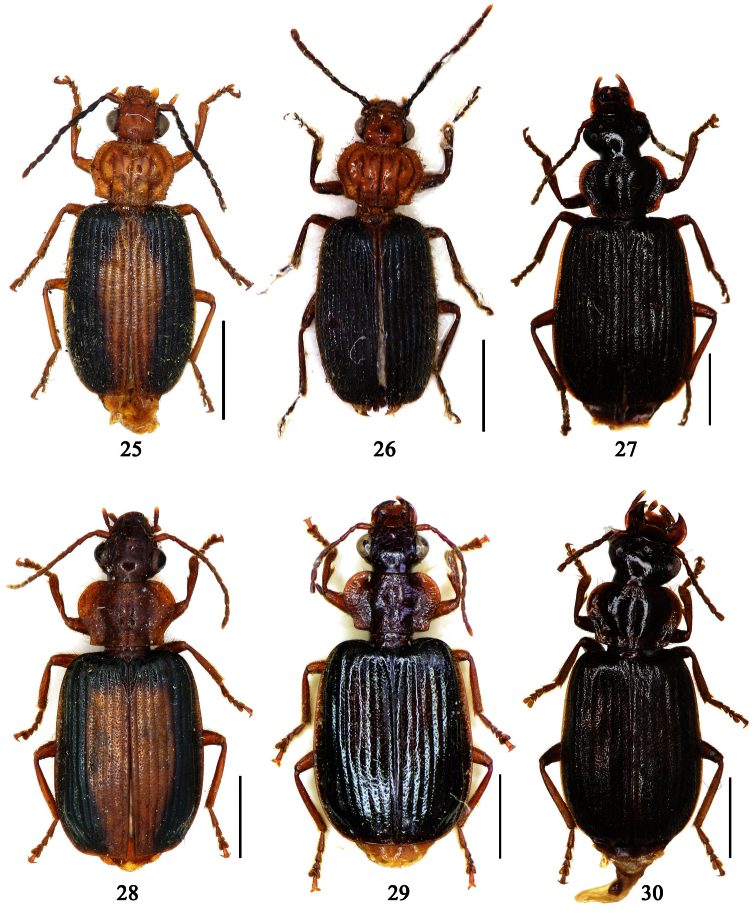
Habitus, scale bars = 2.0 mm: **25** *Dasiosoma bellum* (Habu), a specimen from Guangdong **26** *Dasiosoma bellum* (Habu), a specimen from Sri Lanka **27** *Orionella lewisii* (Bates), a specimen from Japan **28** *Orionella discoidalis* (Bates), a specimen from Yunnan **29** *Orionella kathmanduensis* (Kirschenhofer), a specimen from Nepal **30** *Endynomena pradieri* (Fairmaire), a specimen from Fujian.

Habitus: Figs 25, 26 male genitalia: Fig. 87 female genitalia: Fig. 121

Dasiosoma bellum Habu 1979a: 67, Figs (original: *Teradaia*; type locality: Kukuan (Taiwan); holotype deposited in NIAES).

##### Notes on types.

***Teradaia bella*** Habu: Photograph of holotype is available on the website of National Institute for Agro-Environmental Sciences, Japan (Yoshitake and Kurihara). We didn’t examine the holotype, but it is easy to recognize this remarkable species from literature and photos.

##### Non-type material examined

(Total 6 specimens). 1 male, 3 females (CRS), “S. Vietnam / Nam Cat Tien Nat. Park / 1.–15.5.1994 / Pacholatko & Dembicky”[Fig. 87]. 1 female (SCAU), “Guangdong, Xinfeng, 1983.5, Huang Minlian leg.”[Figs 25, 121]. 1 male (NHML), “Ceylon”; “Thwaites, 67 25”; “*genus ?* / *not far from* / *Lachnoderma*”[Fig. 26].

##### Diagnosis.

Head and pronotum orange yellow, elytra dark blue, disc usually with a large elongate reddish yellow spot; 1st antennomere brown, 2nd and 3rd antennomeres black, then gradually paler from 4th to 7th antennomeres; pronotal lateral margins completely rounded in middle; basal foveae of pronotum straight, subparallel with median line, disc with elongate distinct depression on each side; vertex strongly tumid; tempora abruptly narrowed behind eyes; body slender, ratio EL/EW more than 1.55; apical lamella of aedeagus rather wide.

The unique color, slender body and elongate depression on pronotum readily distinguish this species from all others of the genus.

##### Description.

**Male genitalia** with median lobe of aedeagus strongly bent, ventral and dorsal margins nearly straight before apex in lateral view; strongly bent to right side in dorsal view; apical lamella wide, short, slightly triangular; base of median lobe strongly bent and expanded, basal orifice about 90° relative to preapical shaft. Internal sac with main flagellum long and slender, slightly sinuous, curved to right side; trumpet-form expansion small, short, slightly bent ventrally; secondary flagellum and apical bursa present; membrane adjacent to trumpet-form expansion finely scaled (Fig. 87). **Female genitalia**. Apical segment of ovipositor scimitar-form, inner margin not angulate; length approximately three times basal width; inner margin setose in apical half; apex sharp, with membranous extension long and slender (Fig. 121). Internal reproductive system not studied.

Detailed description of external characters has been provided by Habu (1979a).

##### Distribution

**Map 7.**
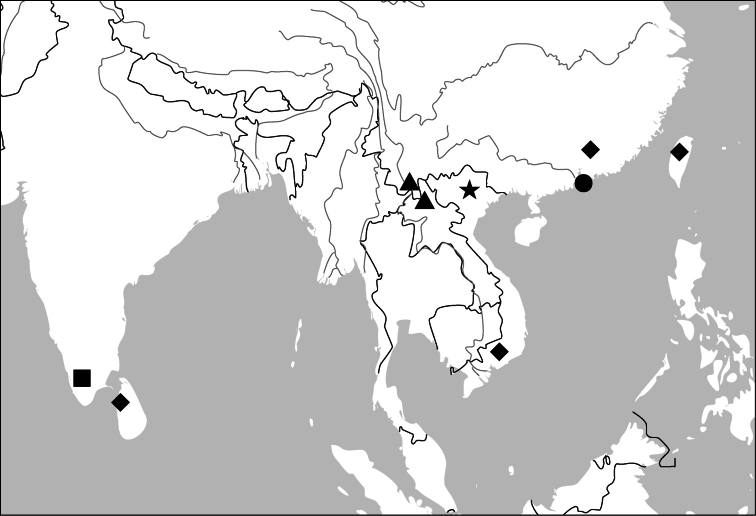
Known distribution of Oriental species of genus *Dasiosoma*: ● *Dasiosoma hirsutum* (Bates) ★ *Dasiosoma maindroni* (Tian & Deuve) ▲ *Dasiosoma quadraticolle* sp. n. ◆ *Dasiosoma bellum* (Habu) ■ *Dasiosoma indicum* (Kirschenhofer).

(Map 7). China (Taiwan, Guangdong); Vietnam; Sri Lanka.

##### Geographical variation.

This rare but widely distributed species is known from four different localities, and their elytral patterns vary from locality to locality. Four specimens from Vietnam have the widest reddish patch, occupying the inner five intervals; a specimen from Guangdong has the patch occupying the inner four intervals (Fig. 25); the holotype from Taiwan has the patch barely reaching the third interval; a specimen from Sri Lanka is nearly without the patch (Fig. 26). Furthermore, the holotype from Taiwan has the hind angles of pronotum slightly acute, while the others have the hind angles subrectangular. We studied male genitalia from two localities. They are very similar in the apical part of median lobe of the aedeagus, but the specimen from Sri Lanka has the median lobe with base less bent and expanded than the one from Vietnam.

##### Remarks.

This species could be most closely related to *Dasiosoma maindroni* (Tian & Deuve). These two species share following characters: (1) vertex strongly tumid, tempora abruptly narrowed behind eyes; (2) aedeagus with base of median lobe strongly bent and expanded, basal orifice about 90° relative to preapical shaft.

#### Dasiosoma indicum

(Kirschenhofer, 2011) comb. n.

http://species-id.net/wiki/Dasiosoma_indicum

Habitus: Fig. 57 male genitalia: Fig. 88

Dasiosoma indicum Kirschenhofer 2011: 68 (original: *Diamella*; type locality: Kerala (India); holotype deposited in CDW).

##### Type examined.

**Holotype** of *Diamella indica* Kirschenhofer, by original designation (CDW): male, body length = 6.4 mm, board mounted, “*2.IX.1989. S-INDIA* / *Kerala: Thekkudy* / *(Peryar-W. L. S.)* / *leg. Riedel*”; “Holotypus / Diamella / indica sp. n. / des. Kirschenhofer 2010” [red label]; “COLL. WRASE / BERLIN” [green label][Figs 57, 88].

##### Diagnosis.

Head and pronotum reddish yellow; elytra dark brown, with two yellowish spots behind middle; pronotal lateral margins completely rounded in middle; basal foveae of pronotum distinctly curved anteromedially, disc without elongate depression; vertex strongly tumid; tempora gradually narrowed behind eyes; body stout, ratio EL/EW 1.30; aedeagus with median lobe moderately expanded at base, apical lamella short and wide. The unique color on elytra, strongly tumid vertex, and tempora gradually narrowed behind eyes readily distinguish this species from all others of the genus.

##### Description.

Body length 6.4 mm; head and pronotum reddish yellow, pronotum with disc somewhat darker; antennae uniform reddish yellow, mouthparts reddish yellow, apices of terminal palpomeres paler, apices of mandibles brown; elytra with background dark brown, slightly cyano-violaceous, each elytron with a large yellow spot behind middle, spot occupying 1st to 5th intervals, spot on each elytron joining at elytral suture; elytral lateral margins, apical margin, apical half of sutural margins, and epipleura reddish yellow; ventral side yellowish. Dorsal side evenly and densely pubescent, pubescence golden; microsculpture indistinct. **Head** with vertex strongly tumid; tempora slightly longer than half length of eyes, gradually narrowed behind eyes; labrum slightly widened to apex, apical margin nearly straight. **Pronotum** wider than head, cordiform, widest slightly before middle; ratio PW/PL 1.55; pronotal base briefly but distinctly lobed; front angles wide; lateral margins strongly expanded in middle, completely rounded, distinctly sinuate before hind angles; hind angles subrectangular, distinct, not projected; disc slightly convex; lateral explanate areas wide and even; basal foveae deep and short, forming a groove, strongly curved anteromedially; disc without elongate depression; median line deep, not reaching apical or basal margins; disc not rugose. **Elytra** wider than pronotum, distinctly widened to apex, ratio EL/EW 1.30; lateral margins slightly depressed at basal one-third, discal depressions indistinct; striae shallowly sulcate, with moderately coarse punctures along them; intervals slightly convex, densely pubescent, primary setigerous pores indistinct; umbilical series of 9th interval indistinct. **Male genitalia** with median lobe of aedeagus strongly bent, ventral and dorsal margins nearly straight before apex in lateral view; strongly bent to right side in dorsal view; apical lamella wide and short, wider than long, apex rounded; base of median lobe moderately bent and expanded, basal orifice about 45° relative to preapical shaft; internal sac with main flagellum long and slender, slightly sinuous, curved to right side; trumpet-form expansion small and short, slightly bent ventrally; secondary flagellum and apical bursa present; membrane adjacent to trumpet-form expansion finely scaled (Fig. 88). **Female genitalia** unknown.

##### Distribution

(Map 7). This species is only known from the type locality: Kerala (India).

##### Remarks.

This species was originally combined with *Diamella* Jedlička, but it is remarkably different from *Diamella kaszabi* in having: (1) elytra evenly and densely pubescent; (2) vertex strongly tumid but posterior supraorbital setae near eyes, insertions not forming a hump; (3) males with two pairs of setae on terminal sternum; (4) median lobe of aedeagus slender, main flagellum long and slender, trumpet-form expansion very small. These characters accord with *Dasiosoma* Britton in the present concept, so we propose a new combination herein.

#### Dasiosoma maindroni

(Tian & Deuve, 2001) comb. n.

http://species-id.net/wiki/Dasiosoma_maindroni

Habitus: Fig. 58 male genitalia: Fig. 89 female genitalia: Figs 122 136

Dasiosoma maindroni Tian & Deuve 2001: 126, Figs 4, 13. (original: *Lachnoderma*; type locality: Tonkin; holotype deposited in MNHN).

##### Type examined.

**Holotype** of *Lachnoderma maindroni* Tian & Deuve, by original designation (MNHN): male, board mounted, “TYPE” [red label]; “TONKIN / Cap. Fouquet”; “MUSEUM PARIS / Ex Coll. M. MAINDRON / Coll. G. BABAULT 1930”; “*Lachnoderma/ bicolor/ m*.”; “*Lachnoderma/ maindroni*”. **Paratype** of *Lachnoderma maindroni* Tian & Deuve: 1 male (SCAU), “PARATYPE”[red label]; “TONKIN / Cap. Fouquet”; “MUSEUM PARIS / Ex Coll. M. MAINDRON / Coll. G. BABAULT 1930”, “*Lachnoderma / maindroni / Tian et Deuve*“[Fig. 58].

##### Non-type material examined

(Total 4 specimens). 1 male (IZAS), “Tonkin *III* / Hoa-Binh *1937*/ leg. A. De Cooman” [pale red label][Fig. 89]. 1 female (IZAS), “*VII* Tonkin *39*/ Hoa-Binh / leg. A. De Cooman” [pale red label][Figs 122, 136]. 2 females (MNHN), “*Hoah binh/ Tonkin*”; “Museum Paris/ 1988/ Coll. J. Negre”.

##### Diagnosis.

Head and pronotum reddish yellow; elytra uniform dark brown; pronotal lateral margins completely rounded in middle; basal foveae of pronotum distinctly curved anteromedially, disc without elongate depressions; vertex strongly tumid; tempora abruptly narrowed behind eyes; body not distinctly slender, ratio EL/EW 1.35–1.40; aedeagus with median lobe strongly expanded at base, apical lamella narrow. The unique color, strongly tumid vertex, and tempora abruptly narrowed behind eyes readily distinguished this species from all others of the genus.

##### Description.

**Male genitalia** with median lobe of aedeagus strongly bent, ventral and dorsal margins nearly straight before apex in lateral view; strongly bent to right side in dorsal view; apical lamella narrow, triangular, slightly longer than basal width; base of median lobe strongly bent and expanded, basal orifice about 90° relative to preapical shaft. Internal sac with main flagellum long and slender, slightly sinuous, curved to right side; trumpet-form expansion very small and short, strongly bent ventrally; secondary flagellum and apical bursa present; membrane adjacent to trumpet-form expansion finely scaled (Fig. 89). **Female genitalia**. Spermatheca simple, moderately long; spermathecal gland inserted at apical two-fifths of spermatheca, not branched, slightly longer than spermatheca; spermatheca slightly expanded and with ring-sculpture at apical one-fourth, basal part of spermatheca without sculpture (Fig. 136). Apical segment of ovipositor scimitar-form, inner margin gradually curved; length four times basal width; inner margin setose in apical half, outer margin setose in apical two-thirds; apex slightly sharp, with membranous extension slightly widened (Fig. 122).

Detailed description of external characters has been provided by Tian and Deuve (2001).

**Distribution** (Map 7). All specimen of this species were collected from Tonkin (North Vietnam).

##### Remarks.

This species was originally combined with genus *Lachnoderma*, but it is closer to *Dasiosoma* than *Lachnoderma* in having: (1) labrum and outer scrobe of mandibles nearly glabrous; (2) mentum tooth simple;(3) pronotal basal foveae deep and groove-like; (4) main flagellum of male genitalia not pointed from apical orifice. So we combine this species with *Dasiosoma* Britton herein.

#### Dasiosoma hirsutum

(Bates, 1873) comb. n.

http://species-id.net/wiki/Dasiosoma_hirsutum

Habitus: Fig. 59 female genitalia: Fig. 137

Dasiosoma hirsutum Bates 1873: 333 (original: *Singilis*; type locality: Hong Kong; lectotype deposited in MNHN); Bates 1883: 285 (*Lachnoderma*); Andrewes 1930d: 188 (*Lachnoderma*; catalogue); Jedlička 1963: 304 (*Lachnoderma*); Tian and Deuve 2001: 126, Figs (*Lachnoderma*; invalid lectotype designation).

##### Type examined.

**Lectotype** of *Singilis hirsutus* Bates, designated herein (MNHN): female, body length = 7.6 mm, board mounted, female genitalia dissected and deposited in microvial pinned under specimen “*Hong/ kong*”; “*Singilis/ hirsutus/ Bates*”; “Ex. Musaeo/ H. W. Bates/ 1892”; “Museum Paris/ 1952/ Coll. R. Oberthür”; “*hirsuta/ Bates*”; “Lectotype/ Singilis hirsutus/ Bates 1873/ Des. SHI H. L. 2011” [red label][Figs 59, 137]

##### Notes on type. 

Tian and Deuve (2001) designated a lectotype for this species. Their lectotype (Fig. 60) was collected in Ceylon in 1892, namely 19 years after the original publication. Obviously it was not examined by Bates when the species was described in 1873. In the original literature, Bates mentioned only one locality, Hong Kong, but did not indicate the number of specimens. The lectotype from Ceylon designated by Tian and Deuve (2001) does not belong to the type series, and their designation of lectotype is invalid. There is a single female in MNHN from Bates’ collection, bearing his determination label, and a label “Hongkong”. It was mentioned by Tian and Deuve (2001) as a paralectotype, but it is the true type and seems to be the unique syntype. We therefore herein designate this specimen as lectotype (Fig. 59) for taxonomic purpose of fixing the name to unique name-bearing type.

##### Diagnosis.

Dorsal side uniform reddish brown; pronotal lateral margins slightly expanded in middle; basal foveae of pronotum distinctly curved anteromedially, disc without elongate depressions; vertex slightly tumid; tempora gradually narrowed behind eyes; body slightly stout, ratio EL/EW 1.44; aedeagus with median lobe moderately expanded at base, apical lamella short and wide. This species is most closely allied with *Dasiosoma quadraticolle* sp. n. Their differences are presented in the diagnosis part of *Dasiosoma quadraticolle*.

##### Description.

Body length 7.6 mm; dorsal side uniform reddish brown, lateral explanate areas of pronotum paler; antennae uniform reddish brown, mouthparts yellowish brown, apices of terminal palpomeres yellow; legs reddish brown; ventral side reddish brown. Dorsal side evenly and densely pubescent, pubescence golden; microsculpture indistinct. **Head** with vertex slightly tumid; tempora subequal to half length of eyes, gradually narrowed behind eyes; labrum slightly widened to apex, apical margin nearly straight. **Pronotum** slightly wider than head, widest at middle; ratio PW/PL 1.43; pronotal base briefly but distinctly lobed; front angles wide; lateral margins slightly expanded in middle, weakly rounded, strongly sinuate before hind angles; hind angles acute, sharp, slightly projected; disc slightly convex; lateral explanate areas wide and even; basal foveae deep and short, groove-like, strongly curved anteromedially; disc without elongate depressions; median line deep, not reaching basal or apical margins; disc not rugose. **Elytra** muchwider than pronotum, slightly widened to apex, ratio EL/EW 1.44; lateral margins slightly depressed at basal one-third, discal depressions indistinct; striae deep, forming distinct grooves, with moderately coarse punctures; intervals convex, densely pubescent, primary setigerous pores indistinct; umbilical series of 9th interval indistinct. **Male genitalia** unknown. **Female genitalia**. Spermatheca straight, claviform, moderately long; apical third slightly widened, with very faint ring-sculpture; spermathecal gland inserted at basal two-fifths of spermatheca, not branched, very fine (Fig. 137). [Lectotype has the apical part of the spermathecal gland damaged, but from the very fine remnant part we speculate that it may be very short, not exceeding the length of the spermatheca.] Apical segment of ovipositor short, triangular, slightly curved outward, inner margin slightly angulate in middle; length two times basal width; inner margin setose in apical half, outer margin in apical two-thirds; apex sharp, with membranous extension narrow.

##### Distribution

(Map 7). This species is only known from the type locality: Hong Kong.

##### Remarks.

The male specimen from Ceylon which was incorrectly designated by Tian & Deuve as lectotype is not identical with the true lectotype from Hong Kong. Compared with the lectotype, the specimen from Ceylon (Fig. 60) differs in having: (1) vertex more tumid; (2) pronotal disc with distinct elongate depression on each side; (3) pronotum wider, lateral margins strongly rounded in middle; (4) striae shallower; (5) head and pronotum with color distinctly paler than elytra. These characters suggest this specimen may be closer to *Dasiosoma bellum* than *Dasiosoma hirsutum*, but with color different from and body stouter than in *Dasiosoma bellum*. This specimen may represent an undescribed species but it is necessary to find more specimens from Sri Lanka or Hong Kong to compare their male or female genital characters and confirm its status. The data for this specimen are as follows: male (MNHN), “*Ceylan / Kandy 1892 / F. Simon*”; “Museum Paris / Ex Coll. M. Maindron / Coll. G. Babault 1930”; “*Lachnoder / hirsutum / Bates*” [probably handwritten by Maindron]; “*Lectotype*”[red label]; “*not type series!* / *Lectotype designation / invalid /* Det. SHI H. L., 2011”[Fig. 60].

#### Dasiosoma quadraticolle

Shi & Liang sp. n.

urn:lsid:zoobank.org:act:00668FEC-546D-47D4-A098-DCA0DE8531E1

http://species-id.net/wiki/Dasiosoma_quadraticolle

Habitus: Figs 24 61 male genitalia: Figs 90 108 female genitalia: Figs 123 138

##### Type material.

**Holotype** (IZAS): male, body length = 6.3 mm, board mounted, genitalia dissected and deposited in microvial pinned under specimen, “China, Yunnan Prov. / Menglun, Botanical / Garden vegetation / N21.91015, E101.28118”; “633m, 2009.11.14 D / TANG Guo Coll. / Institute of Zoology / 西双版纳勐仑植物园”; “HOLOTYPE ♂/ Dasiosoma quadraticolle / new species / Des. SHI H. L. 2011” [red label][Figs 61, 90, 108]. **Paratypes** (22 males, 18 females): **Yunnan**: 1 male (IZAS), “China, Yunnan Prov. / Menglun, Botanical / Garden vegetation / N21.91015, E101.28118”; “633m, 2009.11.14 D / TANG Guo Coll. / Institute of Zoology / 西双版纳勐仑植物园”; “PARATYPE ♂/ Dasiosoma quadraticolle / new species / Des. SHI H. L. 2011” [red label]. 2 females, “云南省西双版纳 / 勐仑镇绿石林公园 / 630m 2006.V.5 / 杨秀帅 采” [Yunnan Prov., Xishuangbanna, Menglun, Lvshilin Park, 630m, 2006.V.5, YANG Xiushuai leg.][Figs 24, 123, 138]; “PARATYPE ♀/ Dasiosoma quadraticolle / new species / Des. SHI H. L. 2011”[red label]. 1 female (IZAS), “云南西双版纳勐仑 / 600米 / 中国科学院” [Yunnna, Xishuangbanna, Menglun, 600m, Chinese Academy of Science]; “1994.IV.24 / 雨林 / 采集者：徐环李” [1994.IV.24, Rain forest / leg. By XU Huanli]; “PARATYPE ♀/ Dasiosoma quadraticolle / new species / Des. SHI H. L. 2011” [red label]. 3 males, 5 females (IZAS), “CHINA, Yunnan Prov., / Xishuangbanna, Menglun, / Botanical Garden; canopy / fogging; 2007.VIII.10; / ZHENG Guo leg.”; “PARATYPE ♂ (or ♀)/ Dasiosoma quadraticolle / new species / Des. SHI H. L. 2011” [red label]. 1 male (IZAS), “Yunnan, Xishuangbanna, / Mengla, Bubeng village; / N21.60599, E101.57072 / 706m, 2012.IV.17, / REN Li leg.”; “PARATYPE ♂ / Dasiosoma quadraticolle / new species / Des. SHI H. L. 2012” [red label]. 3 males, 1 female (IZAS) [preserved in 100% alcohol], “*2009.7.1* 版纳 / 雨林谷560m/ 唐果，姚志远” [2009.VII.01, Yunnan, Xishuangbanna, Yulingu, 560m, Tang G., Yao Z. Y. leg.]; “*Dasiosoma* / *quadraticolle* / *sp.n. 3*♂,* 1*♀ / Det. Shi H.L. 2012”; “*paratype*” [red label]. 3 males, 2 females (IZAS), “CHINA: Yunnan, Xishuan- / gbanna, Menglun Botany / Garden; valley rain forest; / 2009.XII.1; 560m; / Tang G. & Yao Z. Y. leg.”; [the same collecting data but in Chinese]; “PARATYPE / Dasiosoma quadraticolle / new species / Des. SHI H. L. 2011”. 2 females (IZAS), “CHINA: Yunnan, Xishuan- / gbanna, Menglun G213 / arboreal forest; / 2009.XI.24; 590m; / Tang G. & Yao Z. Y. leg.”; [the same collecting data but in Chinese]; “PARATYPE / Dasiosoma quadraticolle / new species / Des. SHI H. L. 2011”. 1 male, 2 females (IZAS), “CHINA: Yunnan, Xishuan- / gbanna, Menglun Botany / Garden; / 2009.XII.1; 560m; / Tang G. leg.”; [the same collecting data but in Chinese]; “PARATYPE / Dasiosoma quadraticolle / new species / Des. SHI H. L. 2011”. 1 male (IZAS), “CHINA: Yunnan, Xishuan- / gbanna, Menglun G213 / bamboo forest; / 2009.XI.21; 620m; / Tang G. & Yao Z. Y. leg.”; [the same collecting data but in Chinese]; “PARATYPE / Dasiosoma quadraticolle / new species / Des. SHI H. L. 2011”. 3 males, 1 female (IZAS), “CHINA: Yunnan, Xishuan- / gbanna, Menglun Botany / Garden, Lyushilin; / 2009.XI.16; 652m; / Tang G. & Yao Z. Y. leg.”; [the same collecting data but in Chinese]; “PARATYPE / Dasiosoma quadraticolle / new species / Des. SHI H. L. 2011”. 5 males, 1 female (IZAS), “CHINA: Yunnan, Xishuan- / gbanna, Menglun Botany / Garden, Lyushilin; / 2009.XI.17; 643m; / Tang G. & Yao Z. Y. leg.”; [the same collecting data but in Chinese]; “PARATYPE / Dasiosoma quadraticolle / new species / Des. SHI H. L. 2011”. **Laos**: 1 male (NHML), “LAOS / *Muong Sai* / le. *19.III*.191*8* / R. Vitalis de Salvaza”; “Bought from / Vitalis de / Salvaza 1928”; “H. E. Andrewes Coll. / B. M. 1945–97.”; “PARATYPE ♂/ Dasiosoma quadraticolle / new species / Des. SHI H. L. 2011” [red label]. 1 female (NHML), “Luang Prabang, / Muong Sai. / 19..III.1918 / R. V. de Salvaza”; “*? genus near* / *Holcoderus*”; “Brit. Mus. / 1921–89”; “PARATYPE ♀/ Dasiosoma quadraticolle / new species / Des. SHI H. L. 2011” [red label].

##### Diagnosis.

Elytra piceous, sometimes disc with an indistinct brownish patch, head and pronotum slightly paler than elytra; pronotum quadrate, lateral margins nearly straight, slightly sinuate before hind angles; basal foveae of pronotum distinctly curved anteromedially, disc without elongate depressions; vertex slightly tumid; tempora gradually narrowed behind eyes; body slightly stout, ratio EL/EW 1.45; aedeagus with median lobe moderately expanded at base, apical lamella narrow.

The dark elytral color, slightly tumid vertex, and pronotal basal foveae distinguish this new species from all other Oriental species of this genus. The new species resembles *Dasiosoma hirsutum* (Bates), but can be distinguished from the latter in having: (1) pronotum nearly quadrate, lateral margins barely expanded, only slightly sinuate before hind angles, hind angles subrectangular, not so sharp or projected; but in *Dasiosoma hirsutum* pronotal lateral margins more expanded, distinctly sinuate before hind angles, hind angles slightly acute, sharp and projected; (2) elytral striae shallower, with finer punctures, intervals slightly convex; but in *Dasiosoma hirsutum* elytral striae deep, forming distinct grooves, with punctures slightly coarser, intervals more convex; (3) dorsal side nearly piceous, but reddish brown in *Dasiosoma hirsutum*; (4) spermatheca with strong ring-sculpture near apex, spermathecal gland inserted at apical two-fifths of spermatheca, but *Dasiosoma hirsutum* with spermatheca apical part only with very faint ring-sculpture, spermathecal gland inserted at basal two-fifths of spermatheca; (5) apical segment of ovipositor with inner margin strongly angulate, but only weakly angulate in *Dasiosoma hirsutum*.

##### Description.

Body length 6.1–7.8 mm, males usually smaller; head and pronotum brown to dark brown, posterior area of vertex and lateral explanate areas of pronotum paler; elytra piceous, usually with a large brownish patch in middle, outline of patch indistinct; antennae uniform reddish brown; mouthparts reddish brown, palpi dark colored, apical half of terminal palpomeres yellow; legs reddish brown to dark brown; ventral side reddish yellow to reddish brown. Dorsal side evenly and densely pubescent, pubescence yellowish; microsculpture indistinct. **Head** with vertex slightly tumid; tempora slightly longer than half length of eyes, gradually narrowed behind eyes; labrum slightly widened to apex, apical margin nearly straight. **Pronotum** slightly wider than head, subquadrate, ratio PW/PL 1.30–1.45; pronotal base briefly but distinctly lobed; front angles wide; lateral margins barely expanded in middle, subparallel, so widest point of pronotum not obvious; lateral margins slightly sinuate before hind angles; hind angles subrectangular, not projected; disc slightly convex; lateral explanate areas slightly widened; basal foveae deep and short, groove-like, strongly curved anteromedially; disc sometimes with very faint elongate depressions; median line deep, nearly reaching apical and basal margins; disc not rugose. **Elytra** muchwider than pronotum, slightly widened to apex, ratio EL/EW 1.45; lateral margins slightly depressed at basal one-third, discal depressions indistinct; striae shallow, not forming distinct grooves, with fine punctures; intervals slightly convex, densely pubescent, primary setigerous pores indistinct; umbilical series of 9th interval indistinct. **Male genitalia** with median lobe of aedeagus strongly bent, ventral and dorsal margins nearly straight before apex in lateral view; strongly bent to right side in dorsal view; apical lamella narrow, triangular, slightly longer than basal width, apex rounded; base of median lobe moderately bent and expanded, basal orifice about 45° relative to preapical shaft. Internal sac with main flagellum long and slender, slightly sinuous, curved to right side; trumpet-form expansion small, slightly elongate, not bent ventrally; secondary flagellum and apical bursa present; membrane adjacent to trumpet-form expansion finely scaled (Fig. 90). **Female genitalia**. Spermatheca straight, slightly claviform, moderately long; apical fourth slightly widened, with distinct ring-sculpture; spermathecal gland inserted at apical two-fifths of spermatheca, not branched, fine, much shorter than spermatheca (Fig. 138). Apical segment of ovipositor short, subtriangular, outer margin nearly straight, inner margin strongly angulate in middle; length two times basal width; inner margin setose in apical part behind angulation, outer margin in apical two-thirds; apex sharp, with membranous extension narrow (Fig. 123).

##### Distribution 

(Map 7). China (Yunnan), Laos.

##### Etymology.

The name “*quadraticolle*” is from the Latin “*quadrat-*” meaning quadrate and “*coll*” meaning neck, referring to the pronotum. This species is named for its unique pronotal shape within the genus.

#### Genus Orionella

Jedlička, 1963

http://species-id.net/wiki/Orionella

Orionella Jedlička 1963: 307; Habu 1979b: 64.
Endynomena auctt. [not Chaudoir 1872]: Habu 1967: 131. [Synonym]

##### Type-species:

*Orionella obenbergeri* Jedlička, 1963 [= *Endynomena lewisii* Bates], by original designation.

##### Diagnosis.

Dorsal side evenly pubescent, elytra with accessory setae on all intervals; mandibles strongly widened; labrum and outer scrobe of mandibles nearly glabrous; pronotum with basal foveae wide and shallow; elytral intervals slightly convex; median lobe of aedeagus slender and straight, not setose around apical orifice, apical lamella more or less bent backwards.

This genus strongly resembles *Endynomena*, but is not closely allied with it given the differences in the male genitalia and male secondary sexual characters. Beyond the remarkable differences in male genitalia (Figs 91–94), *Orionella* is distinguishable from *Endynomena* in having the following characters: (1) striae deeper, intervals slightly convex, striae distinct in apical half, but in *Endynomena* striae shallow, intervals barely convex, striae nearly effaced in apical half; (2) 7th interval without several long setae, but in *Endynomena* with such setae; (3) pronotal base weakly but distinctly lobed, but in *Endynomena* pronotal base hardly lobed; (4) tempora less swollen than in *Endynomena*; (5) apex of mentum tooth truncate, but always rounded in *Endynomena*; (6) males with adhesive hairs only present on 1st and 2nd protarsomeres, but present on 1st to 3rd protarsomeres in *Endynomena*.

##### Generic characters.

Body length 8.3–9.2 mm. Dorsal side reddish brown to dark brown, elytra unicolored or bicolored, without metallic luster; body rather flat; dorsal side densely pubescent, pubescence short, not erect. **Head.** eyes hemispherical, strongly prominent; tempora short, half length of eyes, slightly tumid, gradually narrowed behind eyes; vertex not distinctly tumid. Antennae barely extended to elytral base. Labrum widened to apex, apical margin distinctly emarginate, with faint isodiametric microsculpture, glabrous or with a few very fine short secondary setae; mandibles distinctly widened, outer margins rounded, glabrous on outer scrobes, with some setae arranged along dorsal ridge; terminal maxillary palpomeres slender in both sexes, apices slightly truncate; terminal labial palpomeres slightly widened in both sexes, males somewhat wider, but not typical securiform, apices slightly truncate; ligula with apex slightly projected, with four setae; paraglossae membranous, not longer than ligula, slightly widened, adnate; mentum tooth widened, trapezoid, apex truncate, with two long setae at base, sometimes with a few additional short setae; submentum with two long setae; genae with long setae beneath eyes; gula nearly glabrous except apical part. **Pronotum** transverse, surface densely pubescent; lateral margins with setae along full length, a few long setae present at front angles, four long setae at the greatest width, primary setae on hind angles slightly longer than others; basal foveae wide and shallow; pronotal base weakly but distinctly lobed; lateral margins completely rounded in middle, more or less sinuate before hind angles; hind angles sharp or not. **Elytra** wide, slightly expanded to apex; apex truncate, sutural angles not projected, outer angles completely rounded; basal margination only reaching 3rd interval; basal pores large; striae shallow but distinct, indistinctly punctate; intervals slightly convex; all intervals evenly pubescent, pubescence fine and longish, not erect; primary setigerous pores small, 3rd interval with four pores, 5th interval with one pore near base; 3rd to 6th intervals slightly depressed near middle, 7th and 8th intervals slightly tumid near apex. **Ventral side** with dense pubescence; males with apex of terminal sternum moderately emarginate, with two pairs of setae; females with apex of terminal sternum straight, with two pairs of setae. **Legs** short; protibiae with cleaning spur well developed, distant from inner margin; tarsi widened, 4th tarsomere bifid, claws pectinate. Males with adhesive hairs well developed (two whole rows) on 1st and 2nd protarsomeres and apical half of 1st mesotarsomere; rudimentary (very few near apex, sometimes absent) on 2nd mesotarsomere. **Male genitalia** with median lobe of aedeagus slender, not twisted, nearly straight in lateral view, slightly bent to right side in dorsal view; apical orifice opened apically; apical lamella small, rounded, more or less bent backward; right surface with some fine setae subapically; internal sac with main flagellum slightly thickened, apex distant from apical orifice, trumpet-form expansion small; an elongate scaled area subparallel to main flagellum; apical bursa present; secondary flagellum present. **Female genitalia.** Spermatheca tubular, with ring-sculpture, inserted on bursa copulatrix; spermathecal gland much longer than spermatheca, inserted near base of spermatheca; spermatheca not distinctly bent. Apical segment of ovipositor slender and straight, apex sharp, base gradually widened, apex with membranous extension short and rounded.

##### Distribution

**Map 8.**
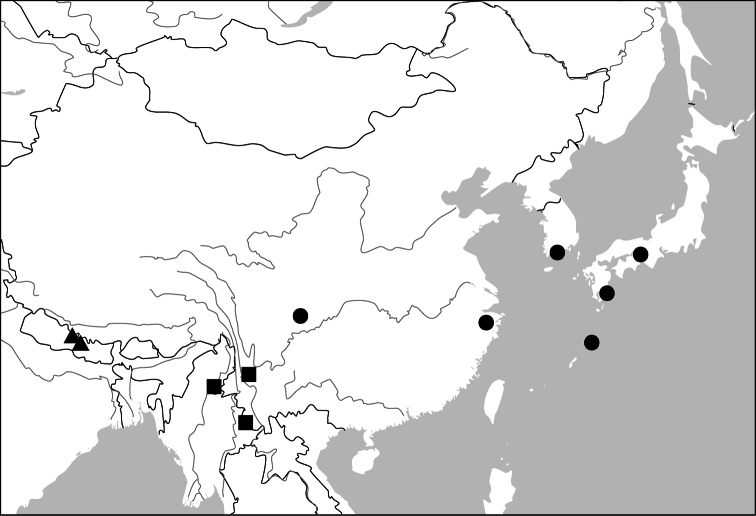
Known distribution of genus *Orionella*: ● *Orionella lewisii* (Bates) ■ *Orionella discoidalis* (Bates) ▲ *Orionella kathmanduensis* (Kirschenhofer).

(Map 8). Japan, Korea, China, Burma, Nepal.

**Monophyly and relationships**. From general appearance, this genus could be allied with *Lachnoderma* or *Dasiosoma*, but genital characters suggest that it could be rather isolated within Physoderina. The monophyly of *Orionella* can be inferred from the following apomorphic character states: (1) median lobe of aedeagus tubular, not laterally compressed; (2) internal sac with main flagellum abruptly terminated before apical orifice; (3) apical lamella bent backward; (4) apical segment of ovipositor straight, not curved outward.

**Key to species of *Orionella* Jedlička**

**Table:** 

1	Elytra reddish brown, with base and lateral margins broadly darkened forming distinct band (Fig. 28); pronotum with lateral margins strongly sinuate before hind angles, hind angles very sharp, forming an acute angle; Yunnan and Myanmar	*Orionella discoidalis* (Bates)
–	Elytra unicolored (Fig. 27) or disc with indistinct reddish patch (Fig. 29), without distinct marginal band; pronotum with lateral margins nearly straight or slightly sinuate before hind angles, hind angles not so sharp	2
2	Head and pronotum reddish brown, elytra dark brown, disc with indistinct reddish patch; pronotal hind angles slightly sharp, subrectangular; internal sac of aedeagus with scaled area less than one-fourth of median lobe; Nepal	*Orionella kathmanduensis* Kirschenhofer
–	Dorsal side uniformly brownish; pronotal hind angles nearly completely rounded; internal sac of aedeagus with scaled area half length of median lobe; Japan, Korea, East China	*Orionella lewisii* (Bates)

#### Orionella lewisii

(Bates, 1873)

http://species-id.net/wiki/Orionella_lewisii

Habitus: Figs 27 62, 63 male genitalia: Figs 91 109 female genitalia: Figs 124 139

Orionella lewisii Bates 1873: 311 (original: *Endynomena*; type locality: Nagasaki (Japan); lectotype deposited in NHML); Bates 1876: 5, Fig. 4 (*Endynomena*); Csiki 1932: 1457 (*Endynomena*; catalogue, misspelled as *lewisi*); Jedlička 1963: 309 (*Endynomena*; misspelled as *lewisi*); Habu 1967: 132, Figs (*Endynomena*); Habu 1979b: 65 (*Orionella*); Habu 1982: 104 (*Orionella*); Uéno et al. 1985: 169, pl. 31 Fig. 11 (*Orionella*); Park et al. 1998: 277 (*Orionella*, Korea)
Orionella obenbergeri Jedlička 1963: 308 Fig. 41 (Type locality: Mt. Minoo (Japan); holotype deposited in NMPC); Habu 1979b: 65 (*Orionella*, synonymized with *lewisii* Bates). [Synonym]

##### Type examined.

**Lectotype** of*Endynomena lewisii* Bates, designated herein (NHML): male, body length = 8.4 mm, board mounted, “345”; “Type” [round label with red circle]; “Japan. / G. Lewis. / 1910-320”; “*Lewisii* / *Bates*”; “LECTOTYPE ♂ / Endynomena lewisii / Bates, 1873 / des. SHI H. L. 2011” [red label][Fig. 62]. **Paralectotype** of *Endynomena* *lewisii* Bates, 1 female, “Japan. / G. Lewis. / 1910-320”; “Ex coll. Brit. Mus.”; “Co-type” [round label with green circle]; “*Endynomena* / *Lewisi* / *Bates*”; “H. E. Andrewes Coll. / B. M. 1945-97”; “PARALECTOTYPE ♀ / Endynomena lewisii / Bates, 1873 / det. SHI H. L. 2011” [red label]. **Holotype** of *Orionella obenbergeri* Jedlička, by original designation (NMPC): male, body length = 8.6 mm, board mounted, “*Mt. Minoo / (OSAKA)*”; “*26-X-1944 / leg. S. Uéno*”; “TYPUS” [red label]; “*Orionella n. g. / Obenbergeri / sp. n. /* det. ING. JEDLIČKA” [pink label][Fig. 63].

##### Notes on types.

***Endynomena lewisii*** Bates: Bates (1873) indicated that this species was described from several specimens from Japan collected by Lewis. In the collection of NHML, we found two specimens bearing Bates’ determination label. These are certainly syntypes and have been labeled as type and cotype by a later worker. There are two other specimens also collected by Lewis from Japan, but from different localities and without Bates’ determination labels. It is better to exclude these two specimens from the type series. We herein designate the male with “type” label in NHML as lectotype (Fig. 62) for the purpose of fixing the name to unique name-bearing type.

***Orionella obenbergeri***Jedlička: The original literature clearly indicated this species was described based on single specimen cited as “type”. So the male in NMPC, the collection of Jedlička, is the holotype (Fig. 63).

##### Non-type material examined

(Total 14 specimens). **Japan**: 1 female (NHML), “Kawachi”; “Japan. /G. Lewis. / 1910-320”. 1 female (NHML), “Hiogo”; “Japan. /G. Lewis. / 1910-320”. 1 male, 2 females (OMNH), “NARA-PREF., Tomio, 1989.VII.18, leg. O. Tominaga”[Fig. 27]. 1 male (OMNH), “OSAKA-PREF, Toyono, 1980.VIII.9, leg. G. Nakata”. **China**:3 males (IZAS), “Zikawei, 1924-3-17”. 1 male (IZAS), “ZHEJIANG, W. Tianmushan, Houshanmen, 500m, 1998.VII.25, Wu Hong leg.”[Fig. 91]. 1 female (IZAS), “ZHEJIANG, W. Tianmushan, Xianrending, 1500m, 1998.VII.29, Wu Hong leg.”[Fig. 139]. 1 female (IZAS), “ZHEJIANG, Tianmushan, 350m, 1999.6.5, Gao Mingyuan leg.”. 1 female (SNUM), “ZHEJIANG, Lin’an, W. Tianmushan, 2008.VI. Huang Hao leg.”[Fig. 124]. 1 female (IZAS), “SICHUAN, Emeishan, 710m, 1979.VI.20, Gao Ping leg.”.

##### Diagnosis.

This species can be distinguished from other species of *Orionella* by: elytra unicolored, brown to dark brown; pronotum with lateral margins straight or slightly sinuate before hind angles; hind angles not distinct, almost completely rounded. This species may be also confused with *Endynomena pradieri* (Fairmaire). The differences are presented in the diagnosis characters of these two genera.

##### Description.

**Male genitalia** with median lobe of aedeagus tubular, not laterally compressed, slightly bent; slightly bent to right side in dorsal view; apical lamella small, placed on right side of apex, strongly bent backward, not exceeding apical orifice. Internal sac with main flagellum slightly thick, sinuous, curved to right side, abruptly terminated before apical orifice; trumpet-form expansion indistinct, very small and narrow; secondary flagellum weakly sclerotized, half length of median lobe; internal sac with an elongate area strongly and coarsely scaled near base, subparallel to main flagellum, half length of median lobe (Fig. 91). **Female genitalia**. Spermatheca straight, slightly claviform, moderately long; apical three-fifths slightly widened, apical two-fifths with distinct ring-sculpture, basal part with finer and indistinct ring-sculpture; spermathecal gland inserted at basal one-fourth of spermatheca, not branched, fine and long, much longer than spermatheca, apical part strongly expanded (Fig. 139). Apical segment of ovipositor straight, not curved outward; length four times basal width; inner margin setose in apical two-thirds, outer margin setose in apical half; gradually narrowed to apex, apex slightly sharp, with membranous extension short, not narrowed (Fig. 124).

Detailed description of external characters has been provided by Habu (1967, 1979b).

##### Distribution

(Map 8). Japan, Korea, China (Zhejiang, Sichuan).

##### Notes on synonym.

Habu (1979b) synonymized *Orionella obenbergeri* Jedlička with *Orionella lewisii* (Bates). We examined types of both species, and confirmed the synonymy.

#### Orionella discoidalis

(Bates, 1892) comb. n.

http://species-id.net/wiki/Orionella_discoidalis

Habitus: Figs 28 64 male genitalia: Fig. 92

Orionella discoidalis Bates 1892: 423 (original: *Endynomena*; type locality: Carin Cheba (Burma); holotype deposited in MSNG); Andrewes 1930d: 163 (*Endynomena*; catalogue); Jedlička 1963: 309 (*Endynomena*); Louwerens 1967: 209 (*Anchista*, doubtful record from Philippines); Kirschenhofer 1994: 1006 (*Endynomena*); Lorenz 1998: 465 (*Anchista*, catalogue).

##### Type examined.

**Holotype** of *Endynomena discoidalis* Bates, by monotypy (MSNG): female, body length = 8.5 mm, board mounted, “Carin Chebà / 900-1100 m. / L/Fea V XII-88”; “TYPUS” [red letter]; “*discoidalis / Bates*”; “*Endynomena / discoidalis / Bates*”; “*Endynomena / discoidalis / typus ! Bates*” [yellow label]; “HOLOTYPUS / *Endynomena / discoidalis / Bates, 1892*” [red label]; “Museo Civico / di Genova”[Fig. 64].

##### Notes on types.

Bates (1892) indicated that this species was described based on a single specimen, so the specimen in MSNG collected by Fea, bearing Bates’ determination label, is the holotype (Fig. 64).

##### Non-type material examined

(Total 2 specimens). **China**: 1 female (HBUM), “Yunnan, Yangbi County, Shunbi, 2004.V.21, Yang Xiujuan, Liu Yushuang leg.”. 1 male (HBUM), “Yunnan, Menglian County, Mengma, 2009.VII.16, Xu Jishan, Zhang Liuxiang etc. leg.”; “22°09'17.6"N, 099°24'32.2"E, 1470m”[Figs 28, 92].

##### Diagnosis.

Elytra reddish brown, with base and lateral margins darkened forming distinct marginal band; pronotum with lateral margins strongly sinuate before hind angles; hind angles distinct, forming acute angles. This species is most closely allied with *Orionella kathmanduensis*, but can be distinguished by the much sharper pronotal hind angles and different elytral color.

##### Description.

Body length 8.5–9.0 mm; head and pronotum reddish brown, lateral explanate areas of pronotum paler; elytral disc reddish brown, with base and lateral margins darkened on 6th to 9th intervals, forming distinct piceous marginal bands conjoined at base, dark area then gradually widened and terminated at 3rd stria near elytral apex; extreme lateral margin of elytra yellowish brown; mouthparts, antennae and legs reddish brown, apices of terminal palpomeres yellow; ventral side uniform reddish brown; dorsal side evenly and densely pubescent, pubescence yellowish, pubescence sparse on vertex and clypeus; microsculpture indistinct. **Head** with vertex nearly flat; tempora slightly longer than half length of eyes, slightly tumid behind eyes; labrum widened to apex, apical margin distinctly bilobed. **Pronotum** wider than head, cordiform, ratio PW/PL 1.50–1.60; pronotal base weakly lobed; front angles widened; lateral margins completely rounded and strongly expanded in middle, widest slightly before middle, lateral margins strongly sinuate before hind angles; hind angles acute, sharp and distinctly projected; disc slightly convex; lateral explanate areas wide; basal foveae wide and shallow; median line fine, nearly reaching basal and apical margins; disc not rugose. **Elytra** slightly widened to apex; striae distinct, not distinctly punctate; 3rd interval with four primary setigerous pores, basal three pores placed in middle of interval, hardly visible, apical one adjacent to 2nd stria, rather distinct; 5th interval with two setigerous pores near base, basal one large and distinct, adjacent to 5th stria, apical one hardly visible; discal depression very shallow; umbilical series of 9th interval composed of 16 pores. **Legs**. Protibiae with cleaning spur finer than in *Orionella lewisii*. **Male genitalia** with median lobe of aedeagus tubular, not laterally compressed, slightly bent; in dorsal view, slightly bent to right side; apical lamella small, placed at right side of apex, distinctly pointed forward, apex only slightly bent backward. Internal sac with main flagellum slightly thick, sinuous, curved to right side, abruptly terminated before apical orifice; trumpet-form expansion small but distinct, slightly widened; secondary flagellum indistinct; internal sac with an elongate area finely scaled near base, subparallel to main flagellum, half length of median lobe (Fig. 92). **Female genitalia**. Apical segment of ovipositor straight, not curved outward; length five times basal width; inner margin setose in apical two-thirds, outer margin setose in apical half; gradually narrowed to apex, apex slightly sharp, with membranous extension slender. Internal reproductive system not studied.

##### Distribution

(Map 8). Myanmar, China (Yunnan).

##### Remarks.

We only studied male genitalia from a teneral specimen of this species from Yunnan. Based on the internal sac of the aedeagus, this species is quite different from *Orionella kathmanduensis*, although very closely allied. In *Orionella discoidalis*, the internal sac has a very long area (half length of median lobe) finely scaled, and trumpet-form expansion wider, more distinct; but in *Orionella kathmanduensis* this area is much shorter (one-fourth length of median lobe) and strongly scaled, and trumpet-form expansion is narrower and less distinct (Figs 92, 93).

#### Orionella kathmanduensis

(Kirschenhofer, 1994) comb. n.

http://species-id.net/wiki/Orionella_kathmanduensis

Habitus: Fig. 29 male genitalia: Fig. 93 109

Orionella kathmanduensis Kirschenhofer 1994: 1012 (Original: *Lachnoderma*; type locality: Kathmandu, Nepal; holotype deposited in NHMW).

##### Non-type material examined

(1 specimen). **Nepal**: 1 male (CRS), “W Nepal, Gorkha Distrikt, Tharpu-Kali Sundhara Baz., 2000–1300m, 10.6.1993, Leg. J. & J. Probst”[Figs 29, 93, 109].

##### Diagnosis.

Elytra dark brown, disc with an indistinct reddish patch; pronotum with lateral margins slightly sinuate before hind angles; hind angles slightly sharp, subrectangular; internal sac of aedeagus with scaled area shorter than one-fourth length of median lobe. This species can be distinguished from other species of the genus by differences in pronotal hind angles and elytral pattern. Male genitalia differ from *Orionella lewisii* in having: (1) scaled area on internal sac very short, less than one-fourth length of median lobe; (2) apical lamella only slightly bent backwards.

##### Description.

Body length 8.3 mm; head and pronotum reddish brown, lateral explanate areas of pronotum paler, vertex somewhat deeper; elytra mostly dark brown, disc with an indistinct large reddish patch ranging from basal one-fifth to three-fifths, and occupying the inner five intervals; elytral lateral margins yellowish brown; mouthparts, antennae and legs reddish brown, apices of terminal palpomeres yellow; ventral side uniform reddish brown. Dorsal side evenly and densely pubescent, pubescence yellowish; microsculpture indistinct. **Head** with vertex nearly flat; tempora slightly longer than half length of eyes, slightly tumid behind eyes; labrum widened to apex, apical margin distinctly bilobed. **Pronotum** wider than head, cordiform, ratio PW/PL 1.65; pronotal base weakly lobed; front angles widened; lateral margins completely rounded and strongly expanded in middle; widest slightly before middle; lateral margins slightly sinuate before hind angles; hind angles subrectangular, slightly sharp; disc slightly convex; lateral explanate areas wide; basal foveae wide and shallow; median line fine, nearly reaching basal and apical margins; disc not rugose. **Elytra** slightly widened to apex; striae distinct, finely punctate; 3rd interval with four primary setigerous pores, basal three hardly visible, placed in middle of interval, apical one distinct, adjacent to 2nd stria; 5th interval with one large setigerous pore near base, adjacent to 5th stria, the remainder nearly invisible; discal depression very shallow; umbilical series of 9th interval composed of 16 pores. **Male genitalia** with median lobe of aedeagus tubular, not laterally compressed, slightly bent to right side in dorsal view; apical lamella small, placed at right side of apex, distinctly pointed forward, apex only slightly bent backward. Internal sac with main flagellum slightly thickened, slightly sinuous, curved to right side, abruptly terminated before apical orifice; trumpet-form expansion small, slightly widened; secondary flagellum weakly sclerotized, half length of median lobe; internal sac with a small area strongly and coarsely scaled near middle, shorter than one-fourth length of median lobe (Fig. 93). **Female genitalia** not studied.

##### Distribution

(Map 8). Nepal.

##### Remarks.

We didn’t examine the holotype of *Lachnoderma kathmanduense* Kirschenhofer deposited in NHMW, but we studied a male from Nepal fitting well with the original description. This species is very similar to *Orionella lewisii* (Bates) both by external and male genital characters, so we move this species to *Orionella* Jedlička herein.

#### Genus Endynomena

Chaudoir, 1872

http://species-id.net/wiki/Endynomena

Endynomena Chaudoir 1872: 186; Jedlička, 1963: 308; Habu, 1979b: 61.
Saronychium Blackburn 1877: 142. Type-species: *Saronychium inconspicuum* Blackburn, 1877 [= *Plochionus pradieri* Fairmaire], by monotypy; Andrewes 1919: 483 (synonymized with *Endynomena* Chaudoir). [Synonym]

##### Type-species:

*Plochionus pradieri* Fairmaire, 1849a, by monotypy.

##### Diagnosis.

Dorsal side evenly pubescent, elytra with accessory setae on all intervals; mandibles strongly widened; labrum and outer scrobe of mandibles nearly glabrous; pronotum with basal foveae wide and shallow; elytral intervals hardly convex; median lobe of aedeagus setose around apical orifice, apical orifice opened dorsally. This genus strongly resembles *Orionella*; distinguishing characters are presented in the diagnosis of *Orionella*.

##### Generic characters.

Dorsal side uniform brownish; body rather flat; dorsal side densely pubescent, pubescence somewhat short, not erect. **Head.** Eyes hemispherical, strongly prominent; tempora short, half length of eyes, distinctly tumid, gradually narrowed behind eyes; vertex not distinctly tumid. Antennae not extended to elytral base. Labrum widened to apex, apical margin distinctly bilobed, with faint isodiametric microsculpture, glabrous or with a few very fine and short secondary setae; mandibles distinctly widened, outer margins rounded, glabrous on outer scrobe, with some setae along dorsal ridge; terminal maxillary palpomeres slender in both sexes, apex slightly truncate in males; terminal labial palpomeres slightly widened in both sexes, wider in males, narrowly securiform, apex slightly truncate; ligula with apex slightly projected, with four setae; paraglossae membranous, not longer than ligula, slightly widened, adnate; mentum tooth short and wide, apex nearly rounded, with two long setae near base; submentum with two long setae; genae with long setae beneath eyes; gula nearly glabrous except apical part. **Pronotum** transverse, surface densely pubescent; lateral margins with setae along full length, a few long setae present at front angles, three or four long setae at the greatest width, primary setae on hind angles slightly longer than others; basal foveae wide and shallow; pronotal base hardly lobed; lateral margins completely rounded in middle, slightly sinuate before hind angles; hind angles not sharp. **Elytra** wide, slightly expanded to apex; apex truncate, sutural angles not projected, outer angles completely rounded; basal margination only reaching 3rd interval; basal pores large; striae indistinct, not distinctly punctate; intervals flat; all intervals evenly pubescent, pubescence fine, not erect; primary setigerous pores small, 3rd interval with four pores, 5th interval with one pore near base, 7th interval with several long setae inserted in large pores; 3rd to 6th intervals slightly depressed near middle, 7th and 8th intervals slightly tumid near apex. **Ventral side** with dense pubescence; males with apex of terminal sternum moderately emarginate, with two pairs of setae; females with apex of terminal sternum straight, with two pairs of setae. **Legs** short; protibiae with cleaning spur well developed, distant from inner margin; tarsi widened, 4th tarsomere bifid, claws pectinate. Males with adhesive hairs well developed (two whole rows) on 1st and 2nd protarsomeres and 1st and 2nd mesotarsomeres; rudimentary (very weak single row) on 3rd protarsomere, apex of 3rd mesotarsomere, and apex of 1st metatarsomere. **Male genitalia** with median lobe of aedeagus slightly stout, not twisted, slightly bent in lateral view, strongly bent to right side in dorsal view; apical orifice large, placed dorsally; apical lamella very small, apex rounded; apical orifice with basal two-thirds surrounded by long setae; internal sac with main flagellum very short, a wide scaled area near middle, apical bursa absent, secondary flagellum present. Left paramere completely rounded, right paramere typical for the subtribe. **Female genitalia.** Apical segment of ovipositor slightly widened, apex slightly rounded, base not widened, apex with membranous extension fine and long. Inner structure of reproductive system not studied.

##### Distribution.

The single species of the genus is widely distributed along the Asia-Pacific range, reaching southern Japan in the north, and southern India in the west, and in many Pacific islands, east to Tahiti and Hawaii, and south to Tonga.

##### Monophyly and relationships.

This genus strongly resembles *Orionella* in external characters, but genital characters show other relationships. The setae around the apical orifice and rudimentary adhesive hairs on the 1st metatarsomere suggest that *Endynomena* is closely related to *Paraphaea* or *Anchista*. The half-reduced main flagellum of the internal sac shows an intermediate status between *Paraphaea* (main flagellum complete) and *Anchista* (main flagellum absent), but the strongly widened mandibles in *Endynomena* contradict this affinity. The special left paramere shape of *Endynomena* may support its monophyly (Fig. 110).

#### Endynomena pradieri

(Fairmaire, 1849a)

http://species-id.net/wiki/Endynomena_pradieri

Habitus: Figs 30 65 66 male genitalia: Figs 94 110 female genitalia: Fig. 125

Endynomena pradieri Fairmaire 1849a: 34 (original: *Plochionus*; type locality: Tahiti Is. (Polynesia); syntype deposited in MNHN); Fairmaire 1849b: 281 (*Plochionus*); Chaudoir 1872: 186 (*Endynomena*, Marquises, Pondichéry); Bates 1889: 283 (*Endynomena*, Saïgon); Andrewes 1919: 483 (*Endynomena*); Andrewes 1927a: 12, Fig. 9 (*Endynomena*, Samoa); Andrewes 1929: 314 (*Endynomena*, Sumatra); Andrewes 1930d: 163 (*Endynomena*; catalogue); Andrewes 1937: 38 (*Endynomena*, Bali); Britton 1938: 109 (*Endynomena*, distribution review); Jedlička 1963: 308, Fig. 42 (*Endynomena*); Darlington 1968: 140 (*Endynomena*, New Guinea); Darlington 1970: 44, Fig. 8-a (*Endynomena*, Micronesia); Shibata 1978: 102 (*Endynomena*, Japan); Habu 1979b: 63, Figs 1–4 (*Endynomena*); Habu 1982: 99 (*Endynomena*); Uéno et al. Sato 1985: 169, pl. 31, Fig. 10 (*Endynomena*).
Saronychium inconspicuum Blackburn 1877: 142 (type locality: Hawaiian Is.; syntype deposited in BMNH); Andrewes 1919: 483 (*Endynomena*, synonymized with *pradieri* Fairmaire). [Synonym]
Endynomena huebneri Fairmaire 1878: 286, (type locality: Tonga; syntype deposited in Museum Hamburg (according to Jedlička 1963)); Andrewes 1927a: 12 (*Endynomena*, synonymized with *pradieri* Fairmaire). [Synonym]
Thyreopterus paroecus Csiki 1915: 164 (type locality: Samoa; syntype deposited in NMW); Andrewes 1927a: 12 (*Endynomena*, synonymized with *pradieri* Fairmaire). [Synonym]

##### Type examined.

**Lectotype** of *Plochionus pradieri* Fairmaire, designated herein (MNHN): male, pin mounted, “TYPE” [red label]; “*Taïti*.” [pink label]; “*Plochionus / Dej. / Pradieri / (type) Fairm / Taïti*”; “Ex Musaeo / L. Fairmaire / 1896”; “LECTOTYPE ♂ / Plochionus pradieri / Fairmaire, 1849 / des. SHI H. L. 2012” [red label][Fig. 65]. **Lectotype** of *Saronychium* *inconspicuum* Blackburn, designated herein (NHML): male, body length = 7.7 mm, board mounted, “Type” [round label with red circle]; “Hawaiian Is. / Rev. T. Blackburn. / 1888-30”; “LECTOTYPE ♂ / Saronychium inconspicuum / Blackburn, 1877 / des. SHI H. L. 2011” [red label][Fig. 66].

##### Notes on types.

***Plochionus pradieri*** **Fairmaire**. The original publication didn’t indicate this species was described based on single specimen, and only mentioned the type locality Tahiti in the title. In the collection of MNHN, we found a male from the collection of Fairmaire bearing his determination label and a “type” label. We herein designate this specimen as lectotype (Fig. 65) for taxonomic purpose of fixing the name to unique name-bearing type.

***Saronychium inconspicuum*** **Blackburn**. Blackburn (1877) didn’t indicate this species was described based on single specimen, and only mentioned the locality “On the plains near Honolulu”. In the collection of NHML, we found a male fitting well with the original description and labeled as type. We herein designate this specimen as lectotype (Fig. 66) for taxonomic purpose of fixing the name to unique name-bearing type.

***Endynomena huebneri* Fairmaire and *Thyreopterus paroecus* Csiki**. We did not find syntypes nor designate lectotypes for these two synonyms.

##### Non-type material examined

(Total 4 specimens). **China**:1 male (CCCC), “Fujian, Jinmen, Gugang, 1995.VII.19, Chen Changchin leg.”[Figs 30, 94, 110]. 1 female (CCCC), “Taiwan, Hualien, Taroko National Park, 1994.IV.5, Lo Chinchi leg.”[Fig. 125]. **India**: 1 female (NHML), “Nevinson Coll. 1918-14”, “Ex coll. Brit. Mus.”, “*Endynomena pradieri*, Compared with type H. E. A.”. **Vanuatu**: 1 male (NHML), “NEW HEBRIDES: Malekula”, “iii.1930. Miss L. E. Cheesman”, “B. M. 1930-395.”, “*Endynomena pradieri*, H. E. Andrewes det”.

##### Diagnosis.

Dorsal side uniform brownish; pronotal base hardly lobed; elytral striae indistinct, intervals not convex; 7th interval with several setigerous pores. This species is similar to *Orionella lewisii*. The differences are presented in the diagnosis part of the genus *Orionella*.

##### Description.

**Male genitalia** with median lobe of aedeagus tubular, laterally compressed, slightly bent; in dorsal view, strongly bent to right side; apical orifice with basal two-thirds surrounded by long setae; apical lamella very small, placed at right side of apex, apex rounded. Internal sac with main flagellum very short, only reaching basal one-third of median lobe; trumpet-form expansion indistinct, very small and narrow; secondary flagellum weakly sclerotized, half length of median lobe; internal sac with two scaled areas, one near base, adjacent to trumpet-form expansion, the other one near middle, just after the termination of main flagellum (Fig. 94). **Female genitalia.** Apical segment of ovipositor slightly widened, length three times basal width; apex slightly rounded; sides nearly parallel, base nearly as wide as apex; apical one-third finely setose; membranous extension very narrow, placed on the outer side of apex (Fig. 125). Inner structure of reproductive system not studied.

Detailed description of external characters has been provided by Habu (1979b).

##### Distribution:

India, Sri Lanka, China (Fujian, Taiwan), Japan, Indo-China Peninsula, Malay Peninsula, the Philippines, Sumatra, Bali, New Guinea, Micronesia, Samoa, Cocos Islands, New Hebrides, Ellice Islands, Hawaii, Tonga, Fiji, Maquesas, Polynesia.

## Plates

**Figures 31–36.**
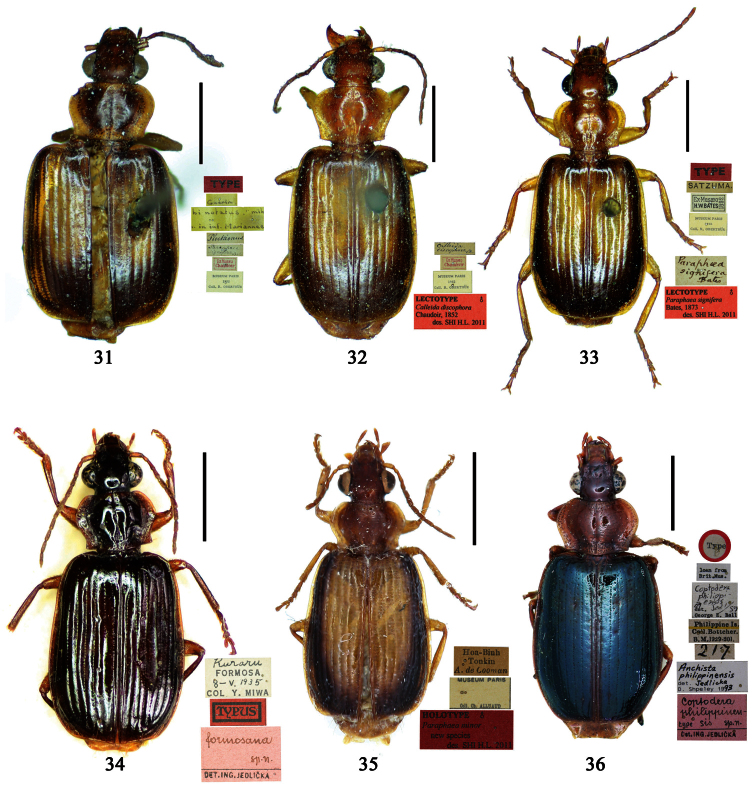
Type materials, scale bars = 2.0 mm: **31** Lectotype of *Plochionus binotatus* Dejean [= *Paraphaea binotata* (Dejean)] **32** Lectotype of *Calleida discophora* Chaudoir [= *Paraphaea binotata* (Dejean)] **33** Lectotype of *Paraphaea signifera* Bates [= *Paraphaea binotata* (Dejean)] **34** Holotype of *Parena formosana* Jedlička [= *Paraphaea formosana* (Jedlička)] **35** Holotype of *Paraphaea minor* sp. n. **36** Holotype of *Coptodera philippinensis* Jedlička [= *Paraphaea philippinensis* (Jedlička)].

**Figures 37–42.**
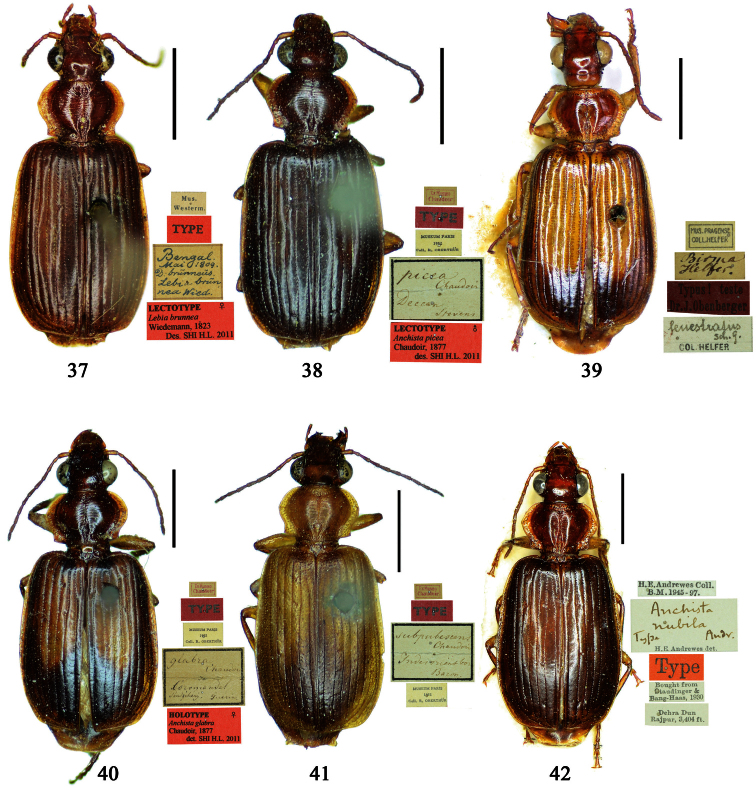
Type materials, scale bars = 2.0 mm: **37** Lectotype of *Lebia brunnea* Wiedemann [= *Anchista brunnea* (Wiedemann)] **38** Lectotype of *Anchista picea* Chaudoir [= *Anchista brunnea* (Wiedemann)] **39** Holotype of *Plochionus fenestratus* Schmidt-Göbel [= *Anchista fenestrata fenestrata* (Schmidt-Göbel)] **40** Holotype of *Anchista glabra* Chaudoir [= *Anchista fenestrata fenestrata* (Schmidt-Göbel)] **41** Lectotype of *Anchista subpubescens* Chaudoir [= *Anchista fenestrata subpubescens* Chaudoir] **42** Lectotype of *Anchista nubila* Andrewes.

**Figures 43–48.**
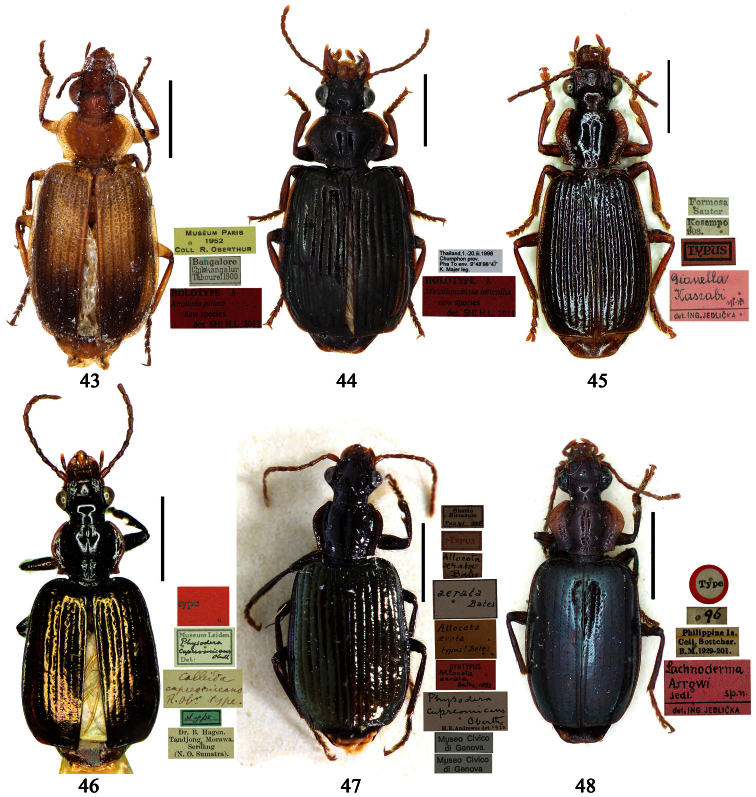
Type materials, scale bars = 2.0 mm: **43** Holotype of *Anchista pilosa* sp. n. **44** Holotype of *Metallanchista laticollis* sp. n. **45** Holotype of *Diamella kaszabi* Jedlička [= *Diamella kaszabi* (Jedlička)] **46** Lectotype of *Calleida cupreomicans* Oberthür [= *Diamella cupreomicans* (Oberthür)] **47** Lectotype of *Allocota aerata* Bates [= *Diamella cupreomicans* (Oberthür)] **48** Holotype of *Lachnoderma arrowi* Jedlička [= *Diamella arrowi* (Jedlička)].

**Figures 49–54.**
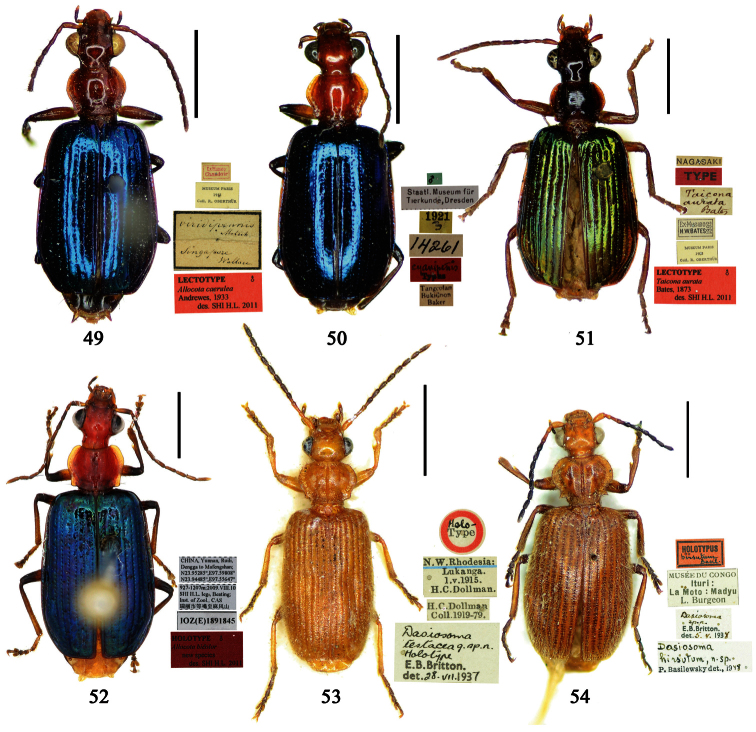
Type materials, scale bars = 2.0 mm: **49** Lectotype of *Allocota caerulea* Andrewes [= *Allocota viridipennis* Motschulsky] **50** Holotype of *Allocota cyanipennis* Heller **51** Lectotype of *Taicona aurata* Bates [= *Allocota aurata* (Bates)] **52** Holotype of *Allocota bicolor* sp. n. **53** Syntype of *Dasiosoma testaceum* Britton **54** Holotype of *Dasiosoma hirsutum* Basilewsky [= *Dasiosoma basilewskyi* nom. n.].

**Figures 55–60.**
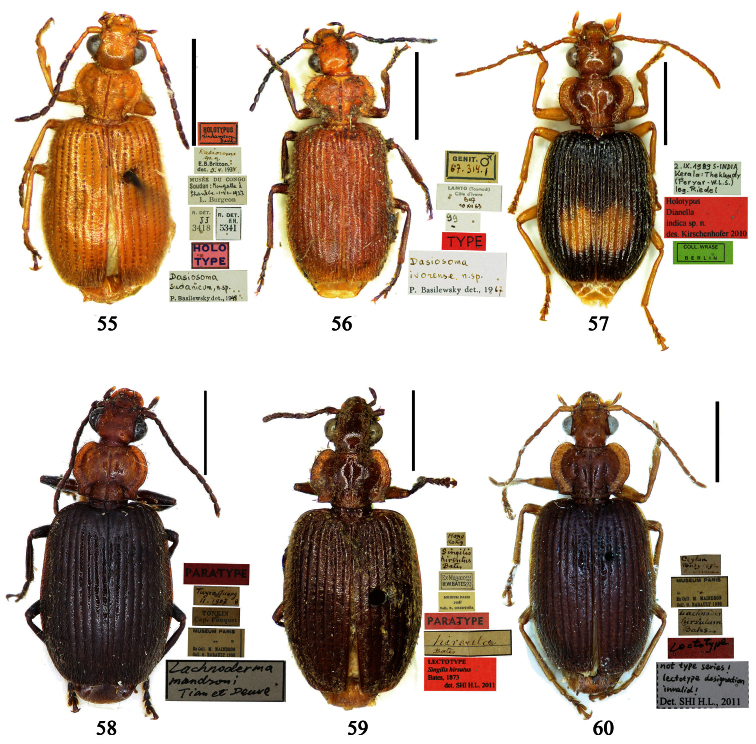
Type materials, scale bars = 2.0 mm: **55** Holotype of *Dasiosoma sudanicum* Basilewsky **56** Holotype of *Dasiosoma ivorense* Basilewsky 57 Holotype of *Diamella indica* Kirschenhofer [= *Dasiosoma indicum* (Kirschenhofer)] **58** Paratype of *Lachnoderma maindroni* Tian & Deuve [= *Dasiosoma maindroni* (Tian & Deuve)] **59** Lectotype of *Singlis hirsutus* Bates [= *Dasiosoma hirsutum* (Bates)] **60** The “lectotype” designated by Tian & Deuve, 2001 for *Singlis hirsutus* Bates.

**Figures 61–66.**
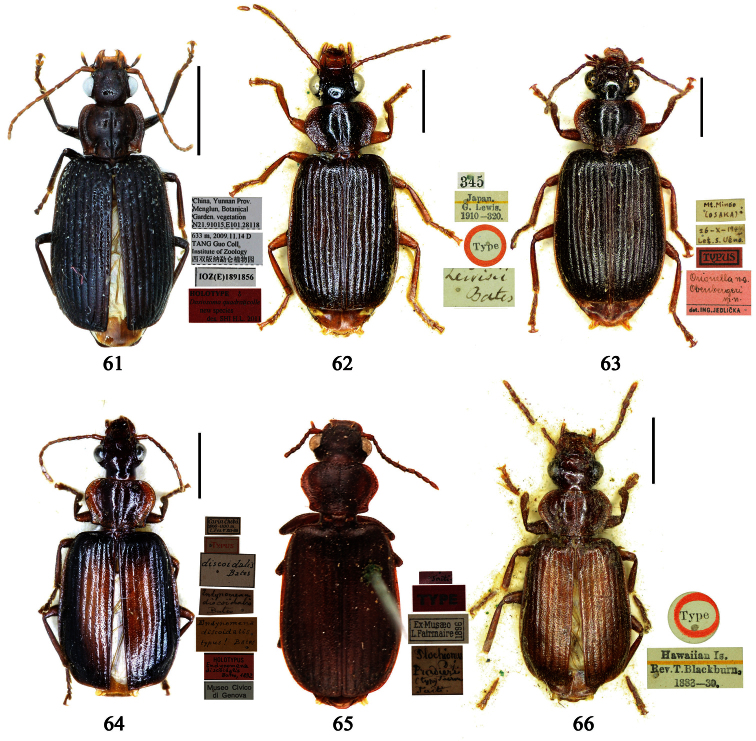
Type materials, scale bars = 2.0 mm: **61** Holotype of *Dasiosoma quadraticolle* sp. n. **62** Lectotype of *Endynomena lewisii* Bates [= *Orionella lewisii* (Bates)] **63** Holotype of *Orionella obenbergi* Jedlička [= *Orionella lewisii* (Bates)] **64** Holotype of *Endynomena discoidalis* Bates [= *Orionella discoidalis* (Bates)] **65** Lectotype of *Plochionus pradieri* Fairmaire [= *Endynomena pradieri* (Fairmaire)] **66** Lectotype of *Saronychium inconspicuum* Blackburn [= *Endynomena pradieri* (Fairmaire)].

**Figure 67.**
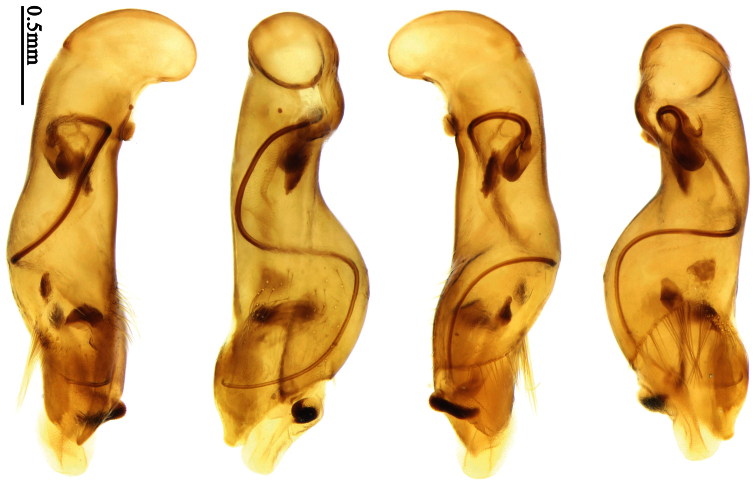
Median lobe of male genitalia, right-lateral, ventral, left-lateral, dorsal views of *Paraphaea binotata* (Dejean), a specimen from Guangxi.

**Figure 68.**
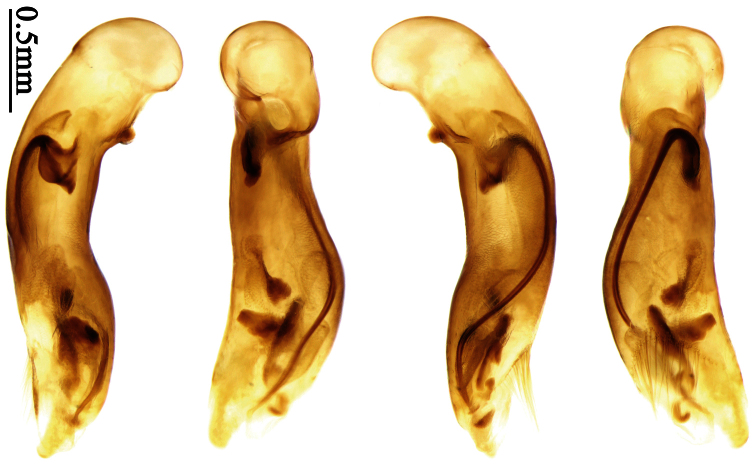
Median lobe of male genitalia, right-lateral, ventral, left-lateral, dorsal views of *Paraphaea formosana* (Jedlička), a specimen from Taiwan.

**Figure 69.**
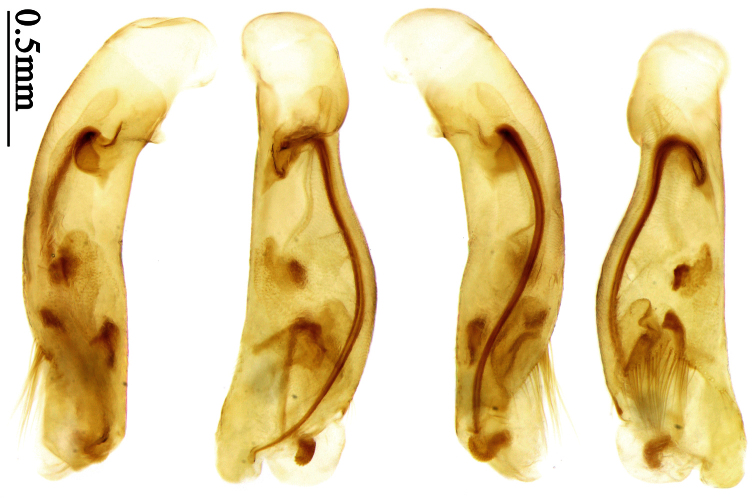
Median lobe of male genitalia, right-lateral, ventral, left-lateral, dorsal views of *Paraphaea minor* sp. n., holotype.

**Figure 70.**
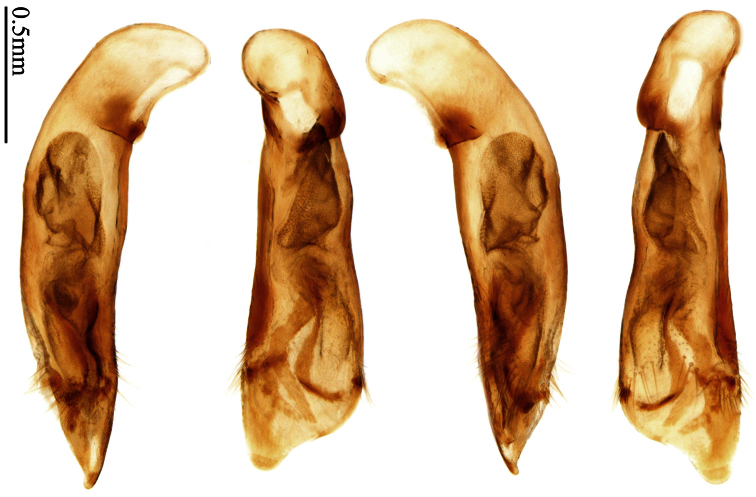
Median lobe of male genitalia, right-lateral, ventral, left-lateral, dorsal views of *Anchista brunnea* (Wiedemann), a paralectotype of *picea* Chaudoir.

**Figure 71.**
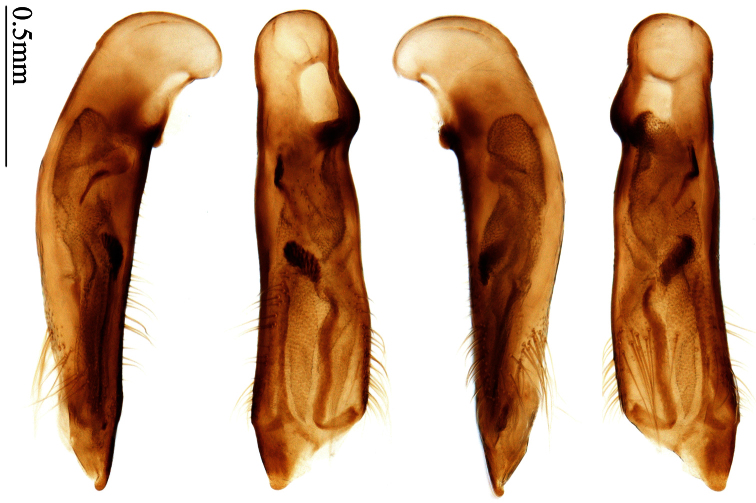
Median lobe of male genitalia, right-lateral, ventral, left-lateral, dorsal views of *Anchista fenestrata fenestrata* (Schmidt-Göbel), a specimen from Chota Nagpore.

**Figure 72.**
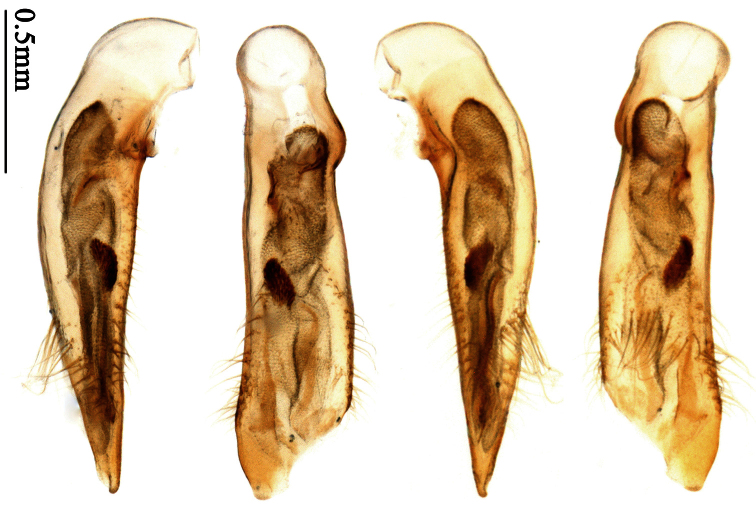
Median lobe of male genitalia, right-lateral, ventral, left-lateral, dorsal views of *Anchista fenestrata subpubescens* Chaudoir, a specimen from Pakistan.

**Figure 73.**
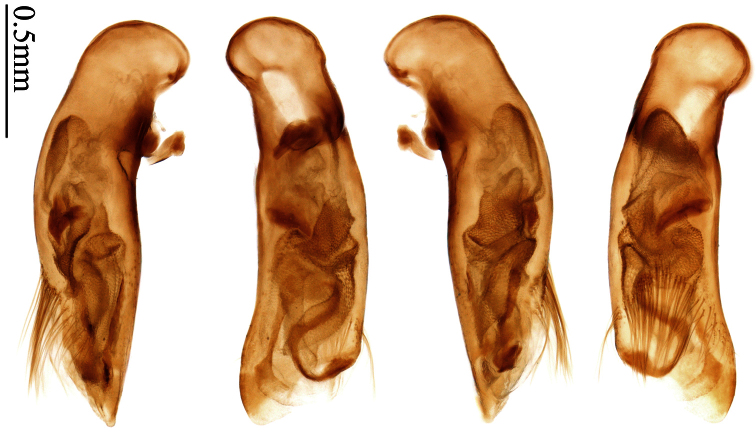
Median lobe of male genitalia, right-lateral, ventral, left-lateral, dorsal views of *Anchista nubila* Andrewes, a specimen from Chota Nagpore.

**Figure 74.**
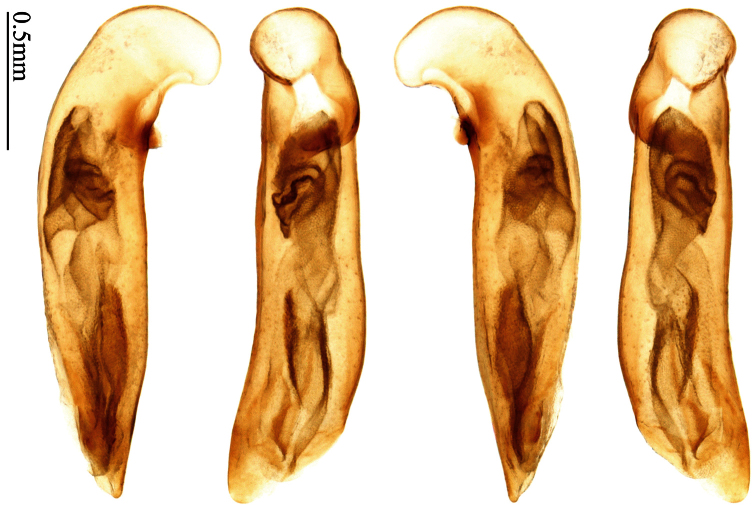
Median lobe of male genitalia, right-lateral, ventral, left-lateral, dorsal views of *Anchista pilosa* sp. n., holotype.

**Figure 75.**
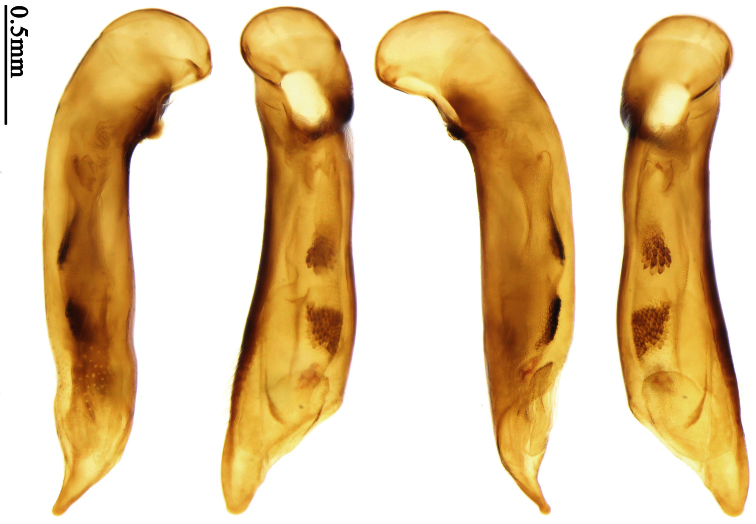
Median lobe of male genitalia, right-lateral, ventral, left-lateral, dorsal views of *Metallanchista laticollis* sp. n., holotype.

**Figure 76.**
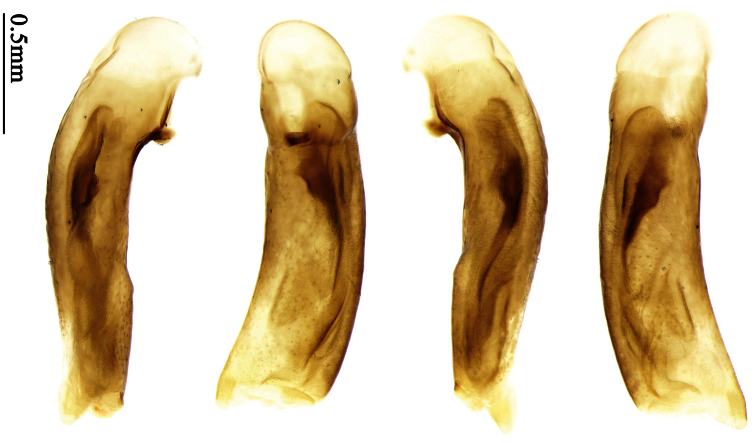
Median lobe of male genitalia, right-lateral, ventral, left-lateral, dorsal views of *Physodera dejeani* Eschscholtz, a specimen from Yunnan.

**Figure 77.**
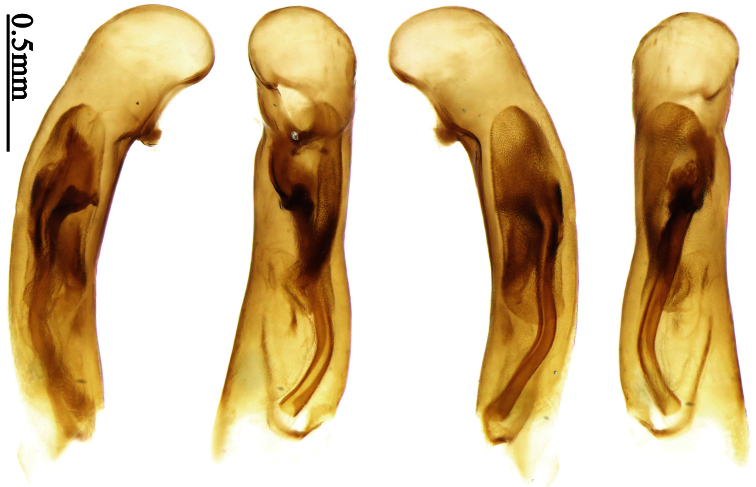
Median lobe of male genitalia, right-lateral, ventral, left-lateral, dorsal views of *Diamella cupreomicans* (Oberthür), a specimen from Hainan.

**Figure 78.**
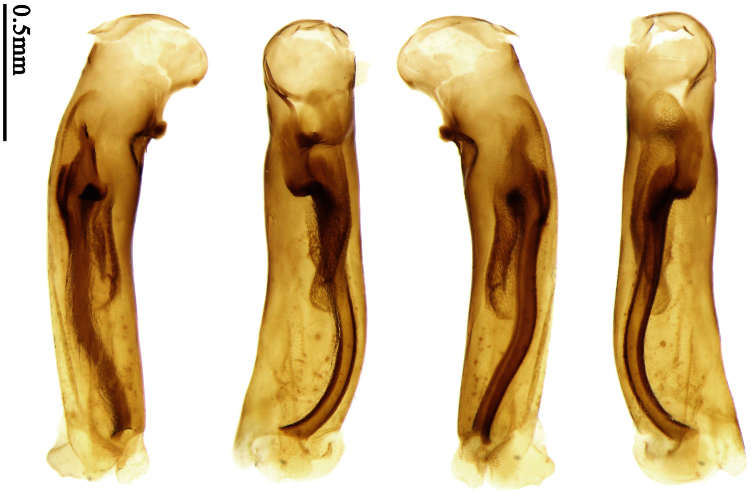
Median lobe of male genitalia, right-lateral, ventral, left-lateral, dorsal views of *Diamella arrowi* (Jedlička), holotype.

**Figure 79.**
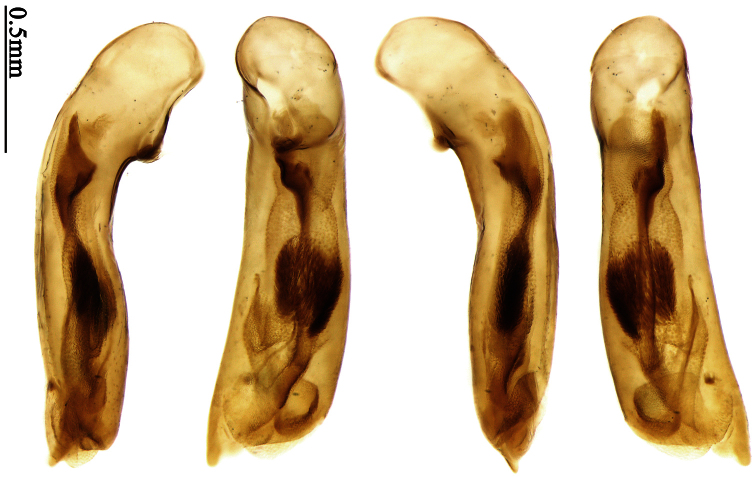
Median lobe of male genitalia, right-lateral, ventral, left-lateral, dorsal views of *Allocota viridipennis* Motschulsky, a specimen from Java.

**Figure 80.**
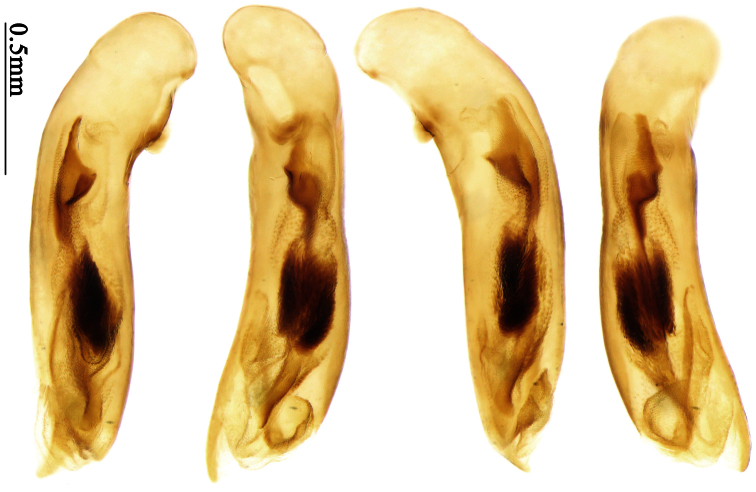
Median lobe of male genitalia, right-lateral, ventral, left-lateral, dorsal views of *Allocota viridipennis* Motschulsky, a specimen from Singapore.

**Figure 81.**
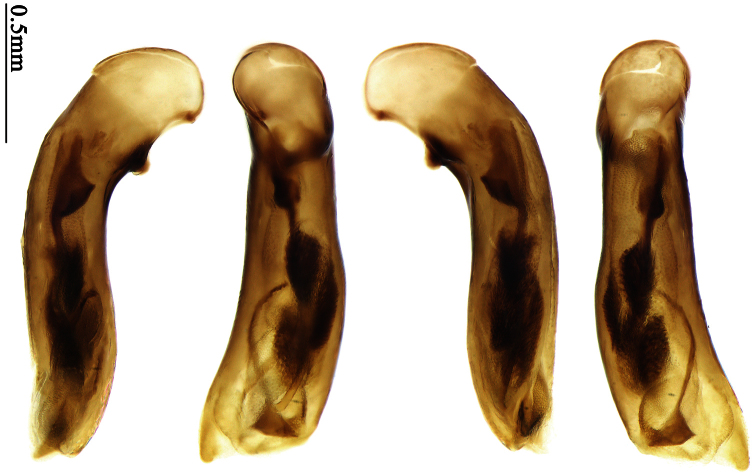
Median lobe of male genitalia, right-lateral, ventral, left-lateral, dorsal views of *Allocota cyanipennis* Heller, a specimen from Philippines.

**Figure 82.**
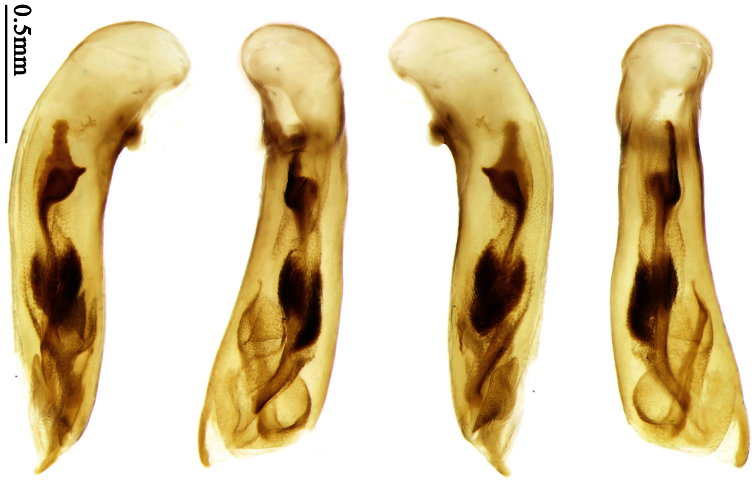
Median lobe of male genitalia, right-lateral, ventral, left-lateral, dorsal views of *Allocota aurata* (Bates), a specimen from Japan.

**Figure 83.**
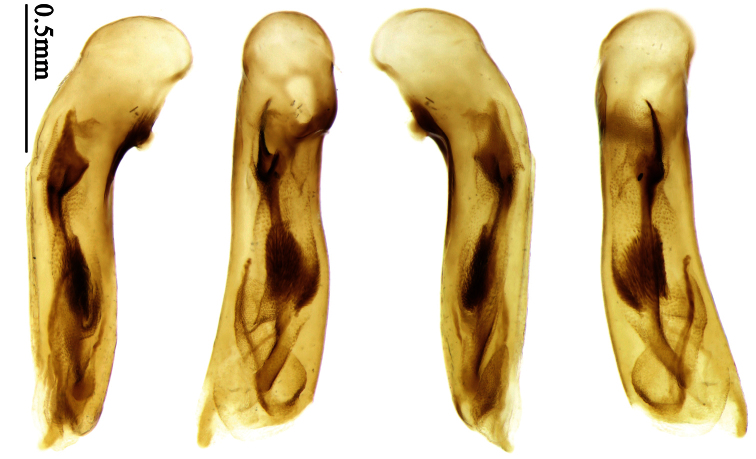
Median lobe of male genitalia, right-lateral, ventral, left-lateral, dorsal views of *Allocota aurata* (Bates), a specimen from Tonkin.

**Figure 84.**
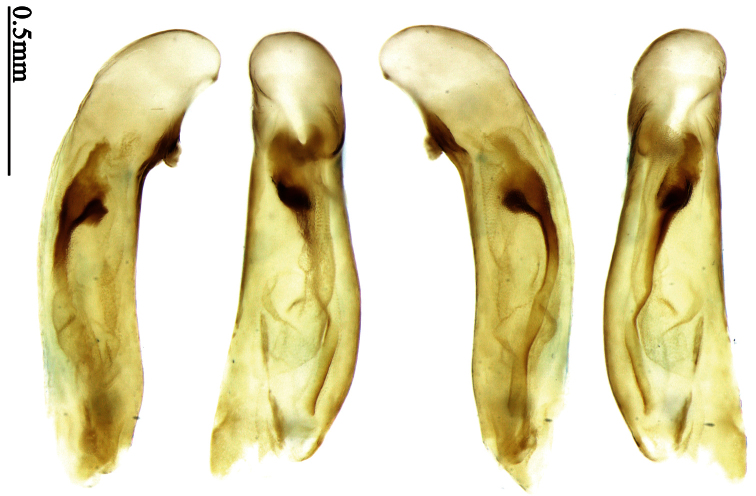
Median lobe of male genitalia, right-lateral, ventral, left-lateral, dorsal views of *Allocota bicolor* sp. n., holotype.

**Figure 85.**
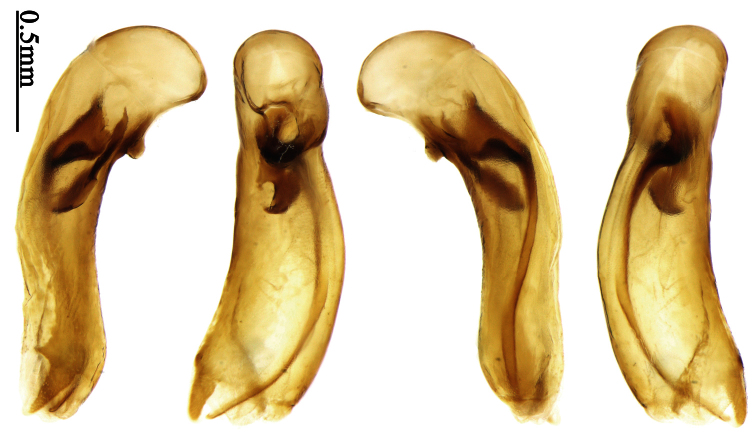
Median lobe of male genitalia, right-lateral, ventral, left-lateral, dorsal views of *Lachnoderma asperum* Bates, a specimen from Taiwan.

**Figure 86.**
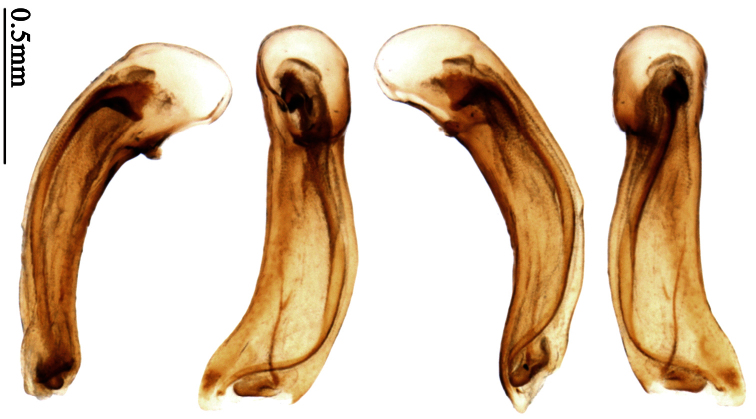
Median lobe of male genitalia, right-lateral, ventral, left-lateral, dorsal views of *Dasiosoma ivorense* Basilewsky, a paratype from Cote d’Ivoire.

**Figure 87.**
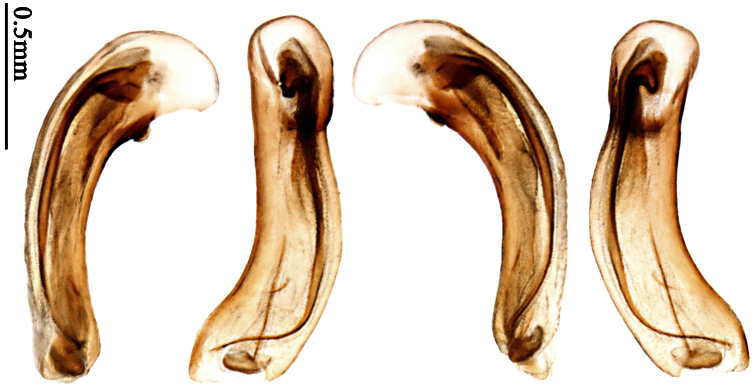
Median lobe of male genitalia, right-lateral, ventral, left-lateral, dorsal views of *Dasiosoma bellum* (Habu), a specimen from Vietnam.

**Figure 88.**
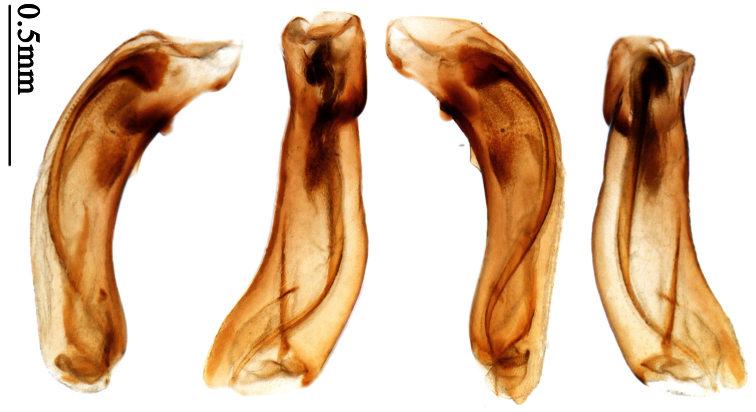
Median lobe of male genitalia, right-lateral, ventral, left-lateral, dorsal views of *Dasiosoma indicum* (Kirschenhofer), holotype.

**Figure 89.**
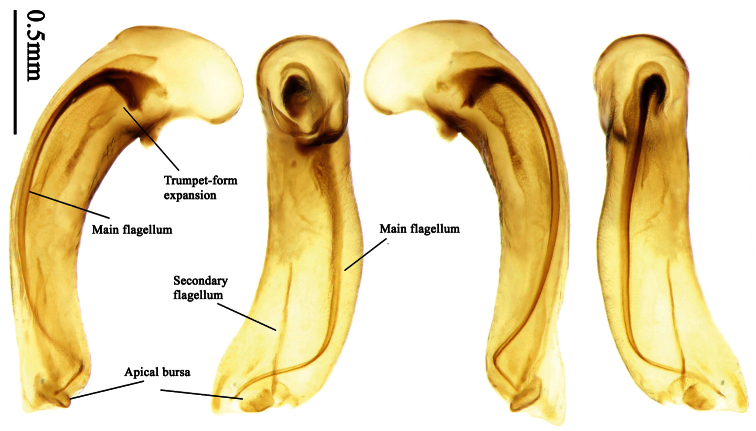
Median lobe of male genitalia, right-lateral, ventral, left-lateral, dorsal views of *Dasiosoma maindroni* (Tian & Deuve), a specimen from Tonkin.

**Figure 90.**
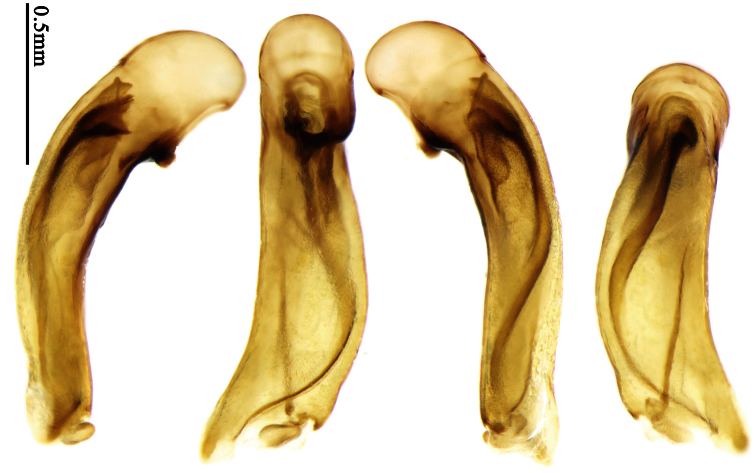
Median lobe of male genitalia, right-lateral, ventral, left-lateral, dorsal views of *Dasiosoma quadraticolle* sp. n., holotype.

**Figure 91.**
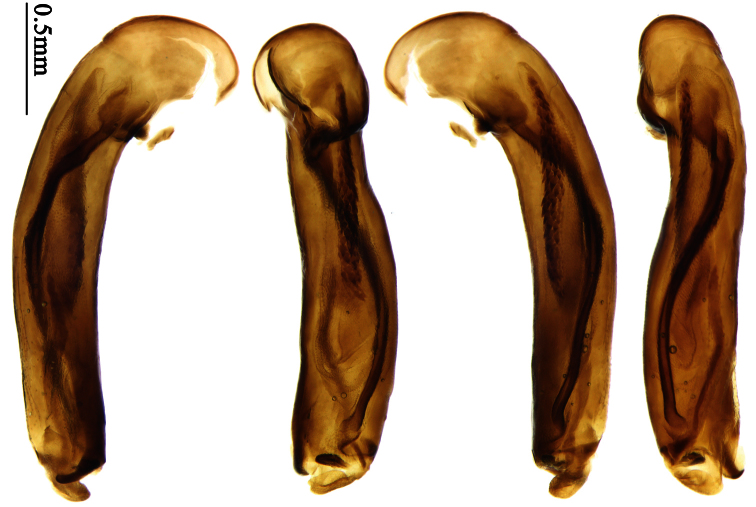
Median lobe of male genitalia, right-lateral, ventral, left-lateral, dorsal views of *Orionella lewisii* (Bates), a specimen from Zhejiang.

**Figure 92.**
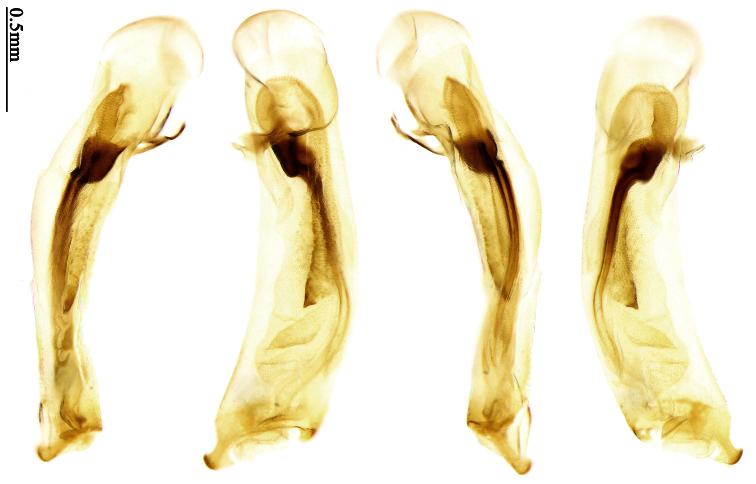
Median lobe of male genitalia, right-lateral, ventral, left-lateral, dorsal views of *Orionella discoidalis* (Bates), a specimen from Yunnan.

**Figure 93.**
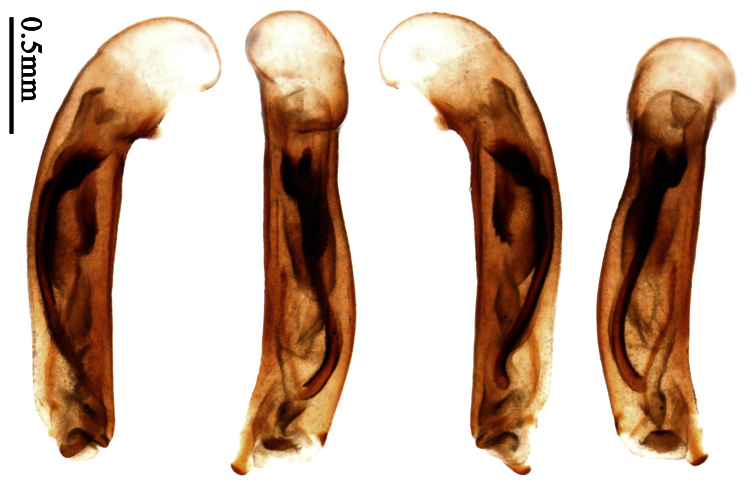
Median lobe of male genitalia, right-lateral, ventral, left-lateral, dorsal views of *Orionella kathmanduensis* (Kirschenhofer), a specimen from Nepal.

**Figure 94.**
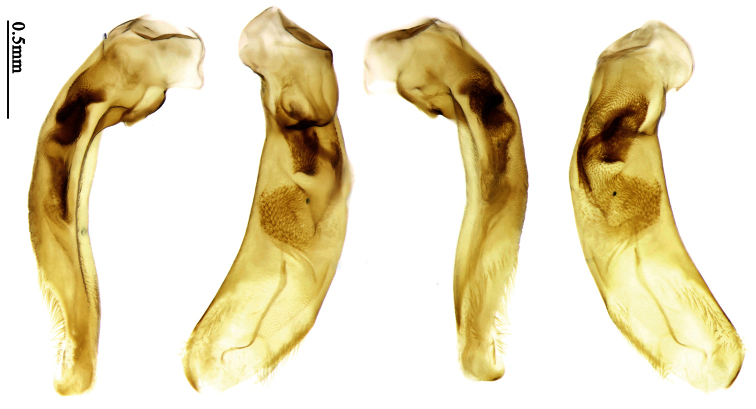
Median lobe of male genitalia, right-lateral, ventral, left-lateral, dorsal views of *Endynomena pradieri* (Fairmaire), a specimen from Fujian.

**Figures 95–110.**
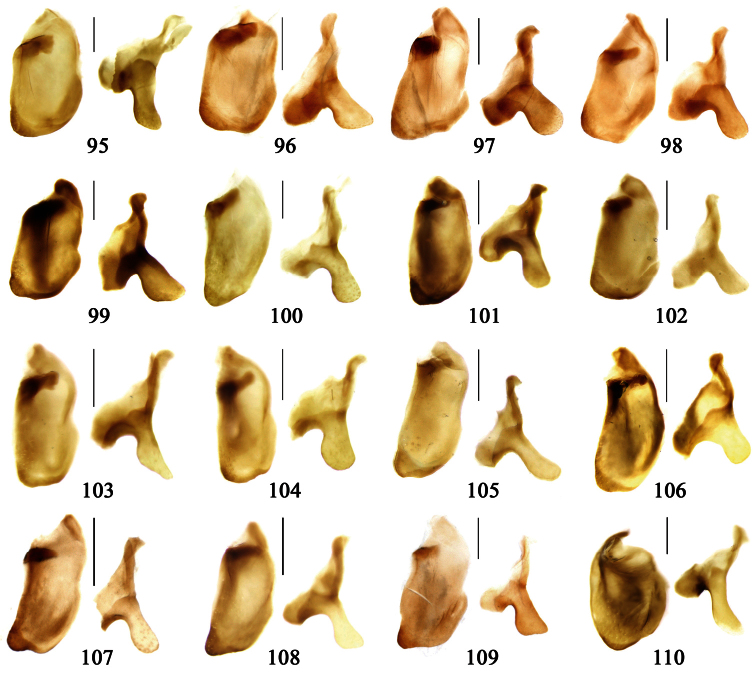
Left and right parameres of male genitalia, scale bars = 0.2 mm: **95** *Paraphaea binotata* (Dejean), a specimen from Guangxi **96** *Paraphaea minor* sp. n., holotype **97** *Anchista brunnea* (Wiedemann), a paralectotype of *picea* Chaudoir **98** *Anchista pilosa* sp. n., holotype **99** *Metallanchista laticollis* sp. n., holotype **100** *Physodera amplicollis* van de Poll, a specimen from Taiwan **101** *Diamella cupreomicans* (Oberthür), a specimen from Yunnan **102** *Diamella arrowi* (Jedlička), holotype **103** *Allocota aurata* (Bates), a specimen from Tonkin **104** *Allocota aurata* (Bates), a specimen from Shaanxi **105** *Allocota bicolor* sp. n., holotype **106** *Lachnoderma asperum* Bates, a specimen from Taiwan **107** *Dasiosoma ivorense* Basilewsky, a paratype from Cote d’Ivoire **108** *Dasiosoma quadraticolle* sp. n., holotype **109** *Orionella kathmanduensis* (Kirschenhofer), a specimen from Nepal **110** *Endynomena pradieri* (Fairmaire), a specimen from Fujian.

**Figures 111–125.**
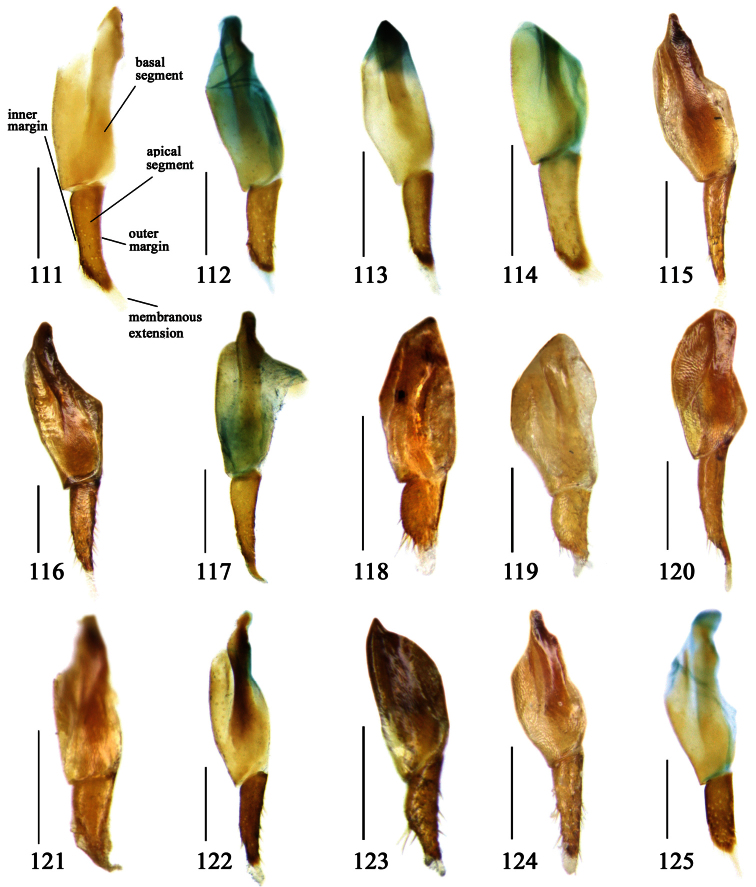
Left ovipositors of females, ventral view, scale bars = 0.2 mm: **111** *Paraphaea binotata* (Dejean), a specimen from Guangxi **112** *Paraphaea minor* sp. n., a paratype from Hainan **113** *Paraphaea philippinensis* (Jedlička), holotype **114** *Anchista fenestrata fenestrata* (Schmidt-Göbel), a specimen from Nepal **115** *Physodera dejeani* Eschscholtz, a specimen from Yunnan **116** *Physodera eschscholtzii* Parry, a specimen from Yunnan **117** *Diamella cupreomicans* (Oberthür), a specimen from Yunnan **118** *Allocota aurata* (Bates), a specimen from Shaanxi **119** *Allocota bicolor* sp. n., a paratype from Guangdong **120** *Lachnoderma asperum* Bates, a specimen from Zhejiang **121** *Dasiosoma bellum* (Habu), a specimen from Guangdong **122** *Dasiosoma maindroni* (Tian & Deuve), a specimen from Tonkin **123** *Dasiosoma quadraticolle* sp. n., a paratype from Yunnan **124** *Orionella lewisii* (Bates), a specimen from Zhejiang **125** *Endynomena pradieri* (Fairmaire), a specimen from Taiwan.

**Figures 126–131.**
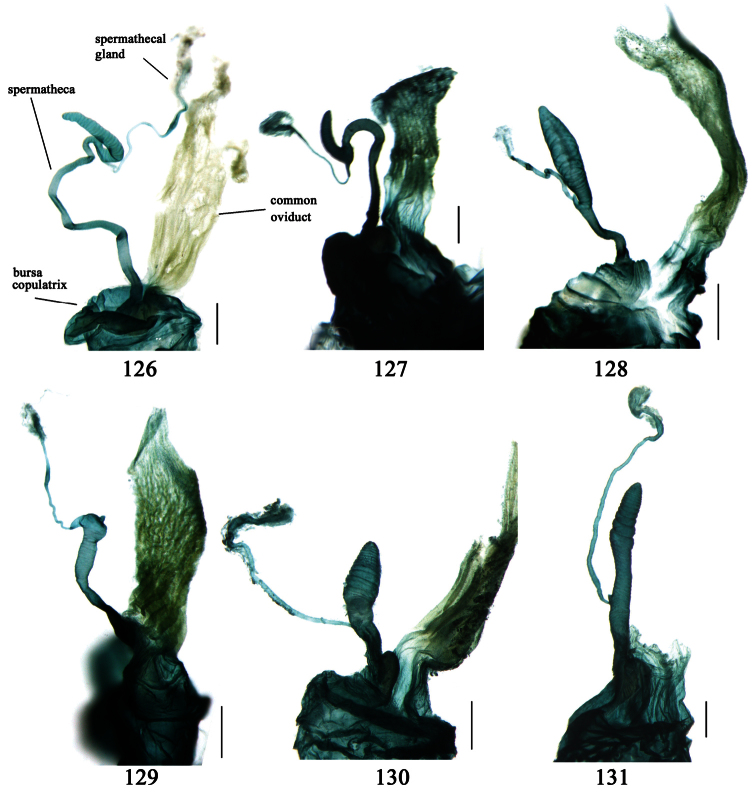
Internal reproductive system of females, scale bars = 0.2 mm: **126** *Paraphaea binotata* (Dejean), a specimen from Guangxi **127** *Paraphaea minor* sp. n., a paratype from Hainan **128** *Paraphaea philippinensis* (Jedlička), holotype **129** *Anchista fenestrata fenestrata* (Schmidt-Göbel), a specimen from Nepal **130** *Physodera dejeani* Eschscholtz, a specimen from Yunnan **131** *Physodera eschscholtzii* Parry, a specimen from Hainan.

**Figures 132–137.**
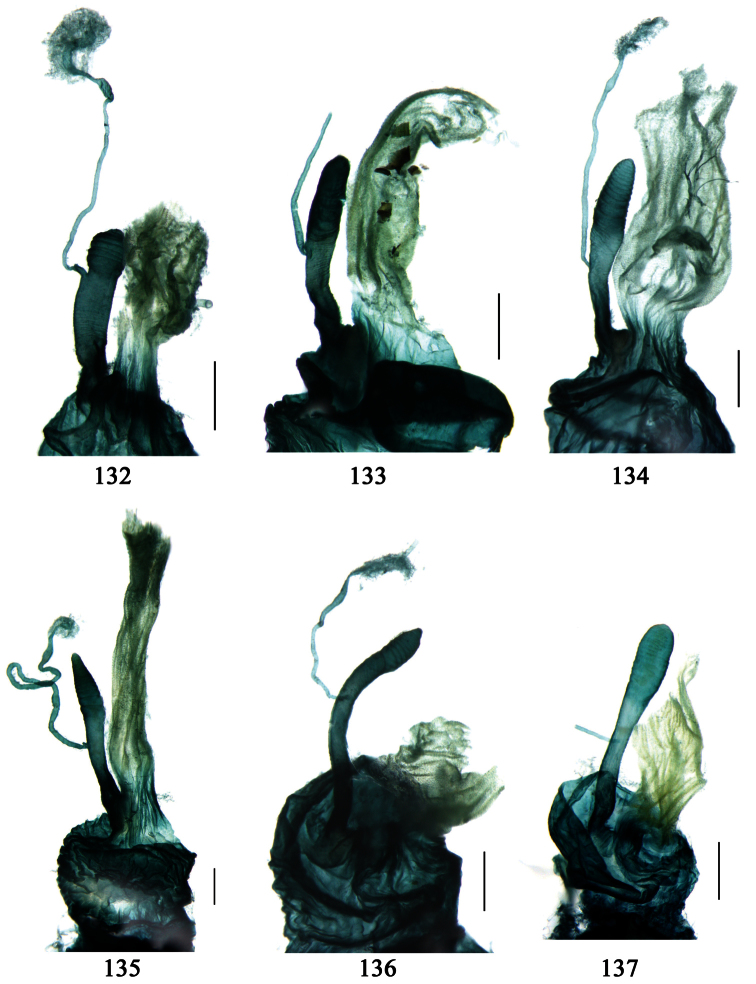
Internal reproductive system of females, scale bars = 0.2 mm: **132** *Diamella cupreomicans* (Oberthür), a specimen from Yunnan **133** *Allocota aurata* (Bates), a specimen from Shaanxi **134** *Allocota bicolor* sp. n., a paratype from Yunnan **135** *Lachnoderma asperum* Bates, a specimen from Zhejiang **136** *Dasiosoma maindroni* (Tian & Deuve), a specimen from Tonkin **137** *Dasiosoma hirsutum* (Bates), lectotype.

**Figures 138–157.**
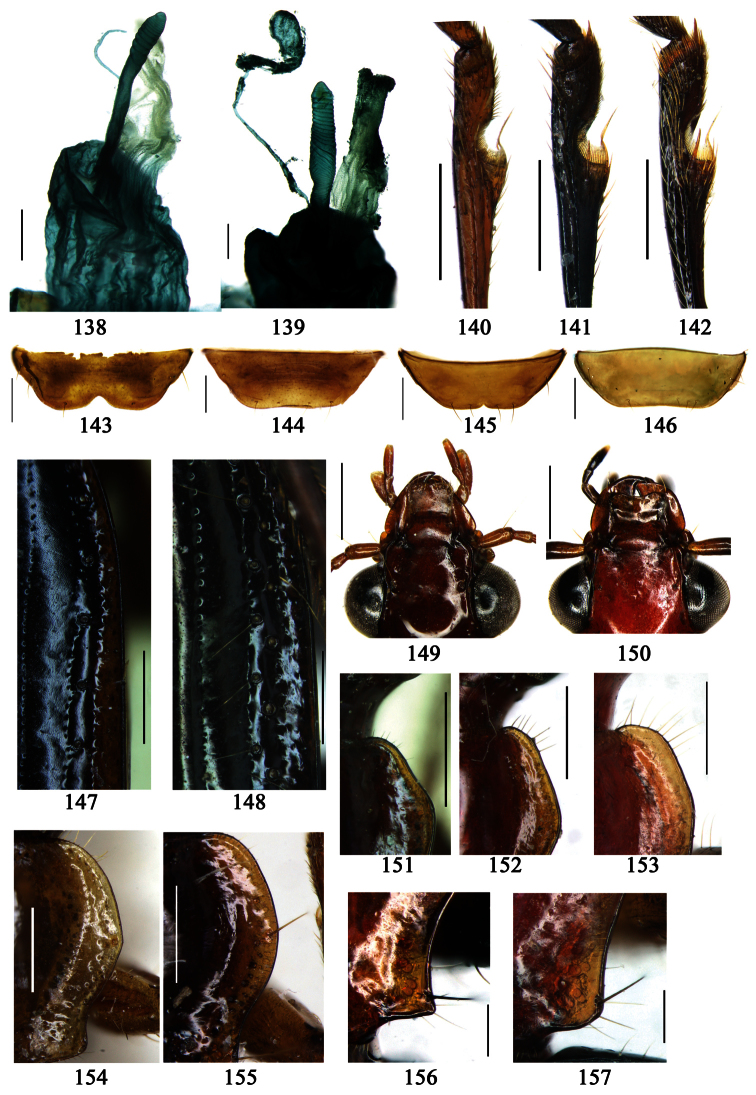
Characters of Physoderina. **138–139** Internal reproductive system of females, scale bars = 0.2 mm: **138** *Dasiosoma quadraticolle* sp. n., a paratype from Yunnan **139** *Orionella lewisii* (Bates), a specimen from Zhejiang. **140–142** Right protibia, ventral view, showing the reduction of cleaning spur, scale bars = 0.5 mm: **140** *Allocota aurata* (Bates) (cleaning spur absent) **141** *Allocota bicolor* sp. n. (cleaning spur fine) **142** *Diamella cupreomicans* (Oberthür) (cleaning spur well developed). **143–146** Terminal sternum, showing the setae and male emargination, scale bars = 0.5 mm: **143** *Paraphaea binotata* (Dejean), male (deeply emarginate, one seta on each side) **144** *Paraphaea binotata* (Dejean) (straight, two setae on each side), female **145** *Allocota aurata* (Bates), male (moderately emarginate, two setae on each side) **146** *Allocota aurata* (Bates), female (straight, two setae on each side, right side unusually with an additional seta). **147–148** Umbilical series of 9th interval, right elytron, scale bars = 0.5 mm: **147** *Paraphaea binotata* (Dejean) (umbilical series placed in one row) **148** *Metallanchista laticollis* sp. n. (umbilical series placed in two rows). **149–150** Head, showing the different shape of mandibles, scale bars = 0.5 mm: **149** *Paraphaea formosana* (Jedlička) (mandibles moderately widen) **150** *Allocota bicolor* sp. n. (mandibles strongly widen). **151–153** Pronotum, showing the different length of setae on front angle, scale bars = 0.5 mm: **151** *Allocota viridipennis* Motschulsky, (setae short and fine) **152** *Allocota cyanipennis* Heller, (setae relative long) **153** *Allocota bicolor* sp. n., (setae very long). **154–155** Pronotum, showing the difference on lateral margin, scale bars = 0.5 mm: **154** *Anchista fenestrata fenestrata* (Schmidt-Göbel), (lateral margin slightly angulated in middle) **155** *Paraphaea formosana* (Jedlička), (lateral margin completely rounded in middle). **156–157** Pronotal hind angle, showing the geographical variation in *Allocota bicolor* sp. n., scale bars = 0.2 mm: **156** *Allocota bicolor* sp. n., a specimen from Guangdong, (hind angle sharp) **157** *Allocota bicolor* sp. n., holotype from Yunnan, (hind angle slightly rounded).

## Supplementary Material

XML Treatment for Physoderina

XML Treatment for Paraphaea

XML Treatment for Paraphaea binotata

XML Treatment for Paraphaea formosana

XML Treatment for Paraphaea minor

XML Treatment for Paraphaea philippinensis

XML Treatment for Anchista

XML Treatment for Anchista brunnea

XML Treatment for Anchista fenestrata fenestrata

XML Treatment for Anchista fenestrata subpubescens

XML Treatment for Anchista nubila

XML Treatment for Anchista pilosa

XML Treatment for Metallanchista

XML Treatment for Metallanchista laticollis

XML Treatment for Metallanchista perlaeta

XML Treatment for Physodera

XML Treatment for Diamella

XML Treatment for Diamella kaszabi

XML Treatment for Diamella cupreomicans

XML Treatment for Diamella arrowi

XML Treatment for Allocota

XML Treatment for Allocota viridipennis

XML Treatment for Allocota cyanipennis

XML Treatment for Allocota aurata

XML Treatment for Allocota bicolor

XML Treatment for Lachnoderma

XML Treatment for Dasiosoma

XML Treatment for Dasiosoma testaceum

XML Treatment for Dasiosoma basilewskyi

XML Treatment for Dasiosoma sudanicum

XML Treatment for Dasiosoma ivorense

XML Treatment for Dasiosoma bellum

XML Treatment for Dasiosoma indicum

XML Treatment for Dasiosoma maindroni

XML Treatment for Dasiosoma hirsutum

XML Treatment for Dasiosoma quadraticolle

XML Treatment for Orionella

XML Treatment for Orionella lewisii

XML Treatment for Orionella discoidalis

XML Treatment for Orionella kathmanduensis

XML Treatment for Endynomena

XML Treatment for Endynomena pradieri
